# Total synthesis and cytotoxicity of the marine natural product malevamide D and a photoreactive analog

**DOI:** 10.3762/bjoc.10.29

**Published:** 2014-02-03

**Authors:** Werner Telle, Gerhard Kelter, Heinz-Herbert Fiebig, Peter G Jones, Thomas Lindel

**Affiliations:** 1 Institute of Organic Chemistry, TU Braunschweig, Hagenring 30, 38106 Braunschweig, Germany; 2 Oncotest Institute for Experimental Oncology GmbH, Am Flughafen 12–14, 79108 Freiburg, Germany; 3 Institute of Inorganic and Analytical Chemistry, TU Braunschweig, Hagenring 30, 38106 Braunschweig, Germany

**Keywords:** cytotoxicity, diazirines, dolastatin analogs, marine natural products, peptides, total synthesis

## Abstract

The marine natural product malevamide D from the cyanobacterium *Symploca hydnoides* was synthesized for the first time. The final peptide coupling linked the dolaisoleuine and dolaproine subunits. The phenyl group of malevamide D was also functionalized with a photoreactive diazirine moiety, which was carried through seven reaction steps. Comprehensive assessment of the cytotoxicity in a panel of 42 human cancer cell lines revealed a geomean IC_70_ value of 1.5 nM (IC_50_ 0.7 nM) for malevamide D, whereas the photoreactive derivative proved to be less active by a factor of at least 200. COMPARE analysis indicated tubulin interaction as likely mode of action of malevamide D.

## Introduction

**Figure 1:**
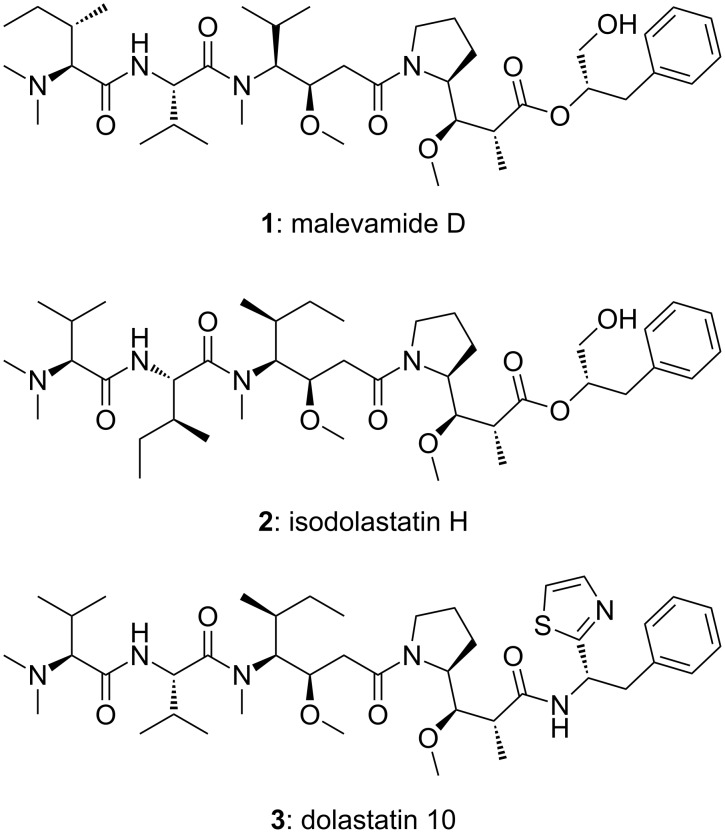
Structures of the strongly cytotoxic marine natural products malevamide D (**1**), isodolastatin H (**2**), and dolastatin 10 (**3**).

The depsipeptide malevamide D (**1**, Figure 1) belongs to the dolastatin class of marine natural products and has been isolated from the cyanobacterium *Symploca hydnoides* by Scheuer and co-workers in 2002 (7.5 mg, 0.014% of the dry weight) [1]. Malevamide D (**1**) was reported to exhibit in vitro cytotoxicity in the subnanomolar range (IC_50_ values about 0.7 nM). Closely related to malevamide D (**1**) is isodolastatin H (**2**), which is also active in vivo, but slightly weaker than dolastatin 10 (**3**) [2]. Although there has been no study regarding the mode of action, it can be assumed that malevamide D (**1**) acts similarly to dolastatin 10 (**3**) by inhibiting the formation of microtubuli from tubulin and, thereby, cell division. Dolastatin-type natural products continue to be tested in clinical trials for cancer therapy [3]. This includes an antibody conjugate of monomethylauristatin E (MMAE), which has been approved for the treatment of Hodgkin lymphoma (Brentuximab vedotin) [4].

In this paper, we report the first total synthesis of malevamide D (**1**) and an assessment of its in vitro cytotoxicity in a panel of 42 cell lines. We also synthesized a diazirinylated analog of **1**, which, by photocrosslinking, might contribute to the understanding of the binding of **1** to tubulin.

## Results and Discussion

### Total synthesis of malevamide D

The natural products malevamide D (**1**), isodolastatin H (**2**), and dolastatin 10 (**3**) share two β-methoxy-γ-amino acid building blocks. The closest structural analog of malevamide D (**1**) is isodolastatin H (**2**) with an identical ester section composed of dolaproine and (2*S*)-3-phenylpropan-1,2-diol. On the N-terminal side, the isopropyl and two *sec*-butyl side chains of isodolastatin H (**2**) are replaced by one *sec*-butyl and two isopropyl side chains, respectively, in the case of malevamide D (**1**).

**Scheme 1:**
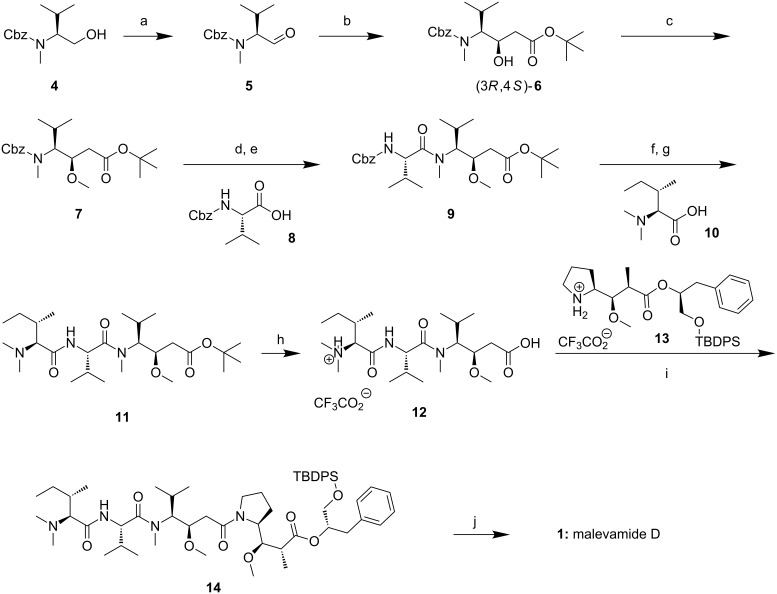
Total synthesis of malevamide D (**1**). a) DMSO (16 equiv), NEt_3_ (5 equiv), pyridine·SO_3_ (5 equiv), 0 °C, 1 h, 91%. b) LDA (2.5 equiv), *t*-BuOAc (1.3 equiv), THF, −78 °C, 1 h; separation of diastereomers, (3*R*,4*S*)-**6**: 47%. c) 1,8-bis(dimethylamino)naphthalene (2.5 equiv), Me_3_OBF_4_ (2.6 equiv), 4 Å MS, 1,2-DCE, 0 °C, 2 h, rt, 16 h, 87%. d) H_2_, Pd/C (5%), MeOH, rt, 1 h. e) **8** (3 equiv), NEt_3_ (4 equiv), DEPCl (2 equiv), DCM, 0 °C, 2 h, rt, 16 h, 80% from **7**. f) H_2_, Pd/C (5%), MeOH, rt, 90 min. g) **10** (3 equiv), NEt_3_ (4 equiv), DEPC (1.5 equiv), DCM, 0 °C, 2 h, rt, 16 h, 83% from **9**. h) TFA, DCM, 0 °C, 90 min, quant. i) **13** (1 equiv), NEt_3_ (6 equiv), DEPC (1.3 equiv), DCM, 0 °C to rt, 16 h, 68%. j) TBAF·3H_2_O (5 equiv), THF/H_2_O 19:1, rt, 3 h, 71%. DEPCl: diethyl phosphorochloridate; DEPC: diethyl phosphorocyanidate.

The total synthesis of **1** adopts the strategy by Yamada and co-workers in their total synthesis of **2**, which sets the first retro cut at the tertiary amide bond between dolaisoleuine and dolaproine [2]. The *tert*-butyl ester **7** of Cbz-protected 3-methoxy-5-methyl-4-(methylamino)hexanoic acid (MMMAH) was obtained in multigram quantities within five steps from Cbz-valine (**8**), adapting a convenient sequence developed for dolaisoleuine by Pettit and co-workers [5]. Cbz-protected *N*-methylvalinol (**4**) [6] was oxidized to the aldehyde **5** under Parikh–Doering conditions, followed by aldol addition of the lithium enolate of *t*-BuOAc (Scheme 1). β-Hydroxyhexanoate **6** was obtained as a mixture of diastereomers (5:4), which was separated by column chromatography (silica) on a multigram scale. Treatment of (3*R*,4*S*)-**6** with Meerwein's salt in presence of proton sponge afforded Cbz-protected MMMAH *tert*-butyl ester **7** in 87% yield. There are also other, enantioselective syntheses of **7** [7] and Boc-protected versions of MMMAH [8–9], but we intended to also have the (3*S*)-epimer of **7** at our disposal.

After hydrogenation of **7**, the resulting secondary amine was coupled with Cbz-valine (**8**) affording dipeptide **9** (DEPCl, diethyl phosphorochloridate), 80% over two steps, Scheme 1). *N*,*N*-Dimethylisoleucine (**10**), obtained by reductive dimethylation of isoleucine with aqueous formaldehyde and H_2_ on Pd/C [10–11], was appended at the N-terminus (DEPC, diethyl phosphorocyanidate) affording tripeptide *tert*-butyl ester **11**. Treatment with TFA liberated the free acid **12**, which was coupled with the trifluoroacetate of dolaproine ester **13**, liberated from the *N*-Boc-protected analog [2].

With the subsequent coupling step affording TBDPS-protected malevamide D (**14**), we initially had difficulties. It proved to be crucial to remove excess of TFA from both coupling partners **12** and **13** completely, by repeated evaporation of DCM solutions. Finally, a coupling yield of 68% was reached following the DEPC protocol. Desilylation (TBAF in THF/H_2_O) afforded **1** (HRESIMS found 755.49257, calcd. for C_40_H_68_NaN_4_O_8_ 755.49294) in 12% yield after 10 steps from valinol **4** (longest linear sequence). The optical rotation of the synthesized material ([α]_D_^28^ −37, *c* 0.09, MeOH) is a little smaller than reported for the isolated natural product ([α]_D_^26^ −55, *c* 0.10, MeOH) [1]. The ^1^H and ^13^C NMR spectra of **1** shows two sets of signals in a ratio of ca. 1:1. Clearly separated in the ^13^C NMR spectrum are, for instance, the ester carbonyl signals of the conformers (174.0 and 173.3 ppm), the carbonyl signals of the pyrrolidine amide (170.5 and 170.6 ppm), and all phenyl carbon signals. Of the eastern section, only three of the four pyrrolidine carbon signals are broad. Most of the carbon signals of the western tripeptide of malevamide D are broadened (see Supporting Information File 1). The ^1^H NMR spectrum shows many broad and overlapping signals, the assignment of which was only possible by careful interpretation of the 2D NMR data set. The only isolated signals belonging to different signal sets are located at δ_H_ 3.84 (one of the diastereotopic OCH_2_ signals) and 5.21 ppm (acylated carbinol). The two sets of signals are probably caused by the presence of both conformers of the acyl pyrrolidine. An assignment of the data sets to either of the two conformers was not possible. The occurrence of conformers has been mentioned by Scheuer and co-workers and is also known for dolastatin 10 [12] and symplostatin 1 [13].

**Scheme 2:**
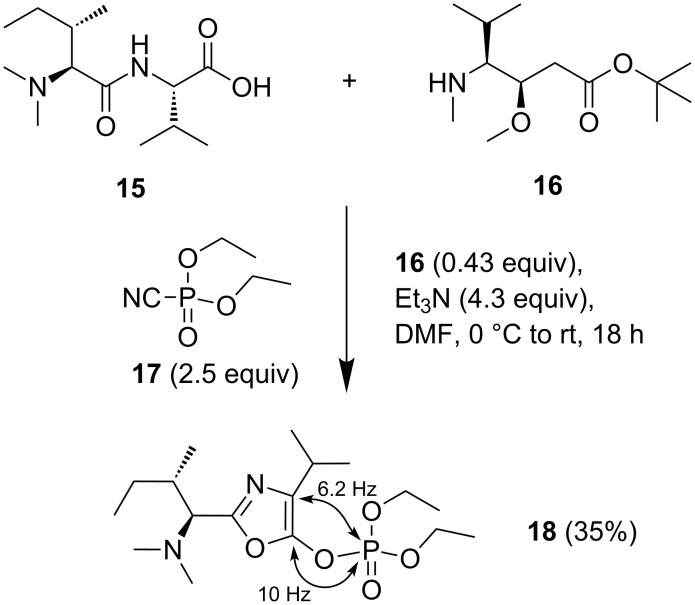
Formation of oxazolylphosphate **18** on attempted DEPC-mediated coupling of dipeptide **15**.

As an alternative route to the N-terminal tripeptide **11**, we also investigated the coupling of the N-terminal dipeptide **15** with MMMAH *tert*-butyl ester (**16**, Scheme 2). However, after treatment of dipeptide **15** with secondary amine **16** (0.43 equiv) and DEPC (**17**, 2.5 equiv), the only isolable product was oxazolylphosphate **18** (35%) without any amide formation occurring. The structure elucidation of **18** required C–P coupling constant analysis, which showed doublet ^13^C NMR signals of oxazole C-5 (10 Hz) and C-4 (6.2 Hz). To the best of our knowledge, this is only the second time that a peptide-derived oxazol-5-ylphosphate has been characterized. Earlier, Boyd and co-workers had converted an oxazolone to an oxazolyl phosphate by treatment with excess diphenyl chlorophosphate [14]. In our case, a similar pathway is to be assumed with the oxazolone being formed first. The secondary amine **16** appears to react too slowly to be able to compete with oxazolone formation.

### Diazirinyl-substituted malevamide D

Encouraged by the strong cytotoxicity of **1**, we addressed the synthesis of a photoactivatable analog. The question was whether such a derivative would still be cytotoxic. The phenyl ring of **1** was chosen as location of a trifluoromethyldiazirinyl substituent. Pettit and co-workers have shown that introduction of a phosphate moiety at the *p*-position of the phenyl ring of auristatin PE did not lead to loss of cytotoxicity [3]. Recently, we have synthesized a hemiasterlin derivative with a diazirinylated indole moiety by incorporation of a thermally stable L-phototryptophan carrying a 1-azi-2,2,2-trifluoroethyl unit [15].

**Scheme 3:**
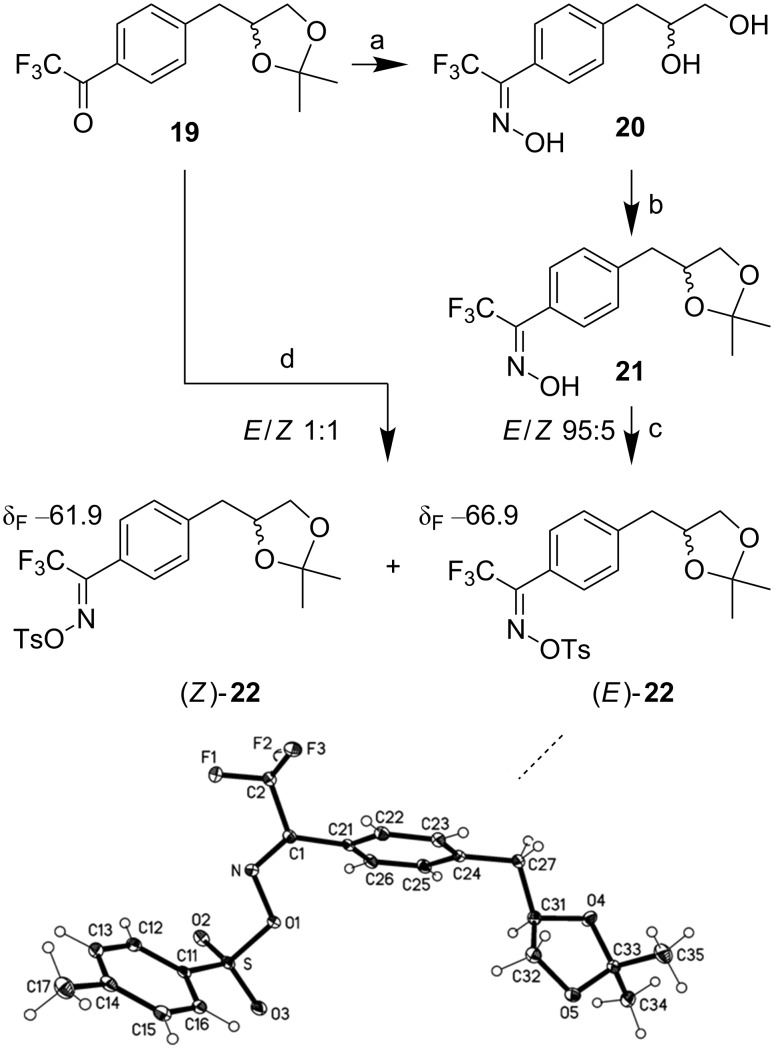
Synthesis of tosyloximes (*Z*)-**22** and (*E*)-**22**, X-ray structure of (*E*)-**22**. a) NH_2_OH·HCl (1.5 equiv), pyridine, reflux, 2 h, 80%. b) 2,2-dimethoxypropane (12.4 equiv), *p*-TsOH·H_2_O (5 mol %), DMF, rt, 1 h, 95%. c) *p*-TsCl (1.05 equiv), NEt_3_ (1.3 equiv), DCM, 0 °C to rt, 18 h, 83%. d) (i) NH_2_OH·HCl, NaOH, EtOH, pyridine, reflux (for details see Supporting Information File 1); (ii) *p*-TsCl (1.05 equiv), NEt_3_ (1.3 equiv), DCM, 0 °C to rt, 18 h, 74%.^19^F NMR chemical shifts (ppm) in CDCl_3_.

Racemic photo phenylpropanediol **25** was obtained starting from aryl(trifluoromethyl)ketone **19** (Scheme 3), which itself was synthesized via Grignard monoallylation of 1,4-dibromobenzene, followed by dihydroxylation, dioxolane protection and trifluoroacetylation of the remaining brominated position [16]. Refluxing of ketone **19** with NH_2_OH·HCl in pyridine led to nearly quantitative hydrolysis of the dioxolane affording diol oxime **20** as 95:5 mixture of *E*- and *Z*-isomers. However, if solid NaOH was added to the reaction mixture and heated for several hours, the dioxolane survived, affording a 1:1 mixture of *E*/*Z*-oximes, which were tosylated after short work-up to (*E*)- and (*Z*)-**22**.

Reprotection of the diol unit of **20** and tosylation of **21** afforded tosyloxime (*E*)-**22**, which crystallized overnight from the neat mixture and was analyzed by X-ray crystallography [17]. Thus, it became possible to assign the ^19^F NMR chemical shifts of both isomers ((*E*)-**22**: δ_F_ −66.9; (*Z*)-**22**: δ_F_ −61.9).

**Scheme 4:**
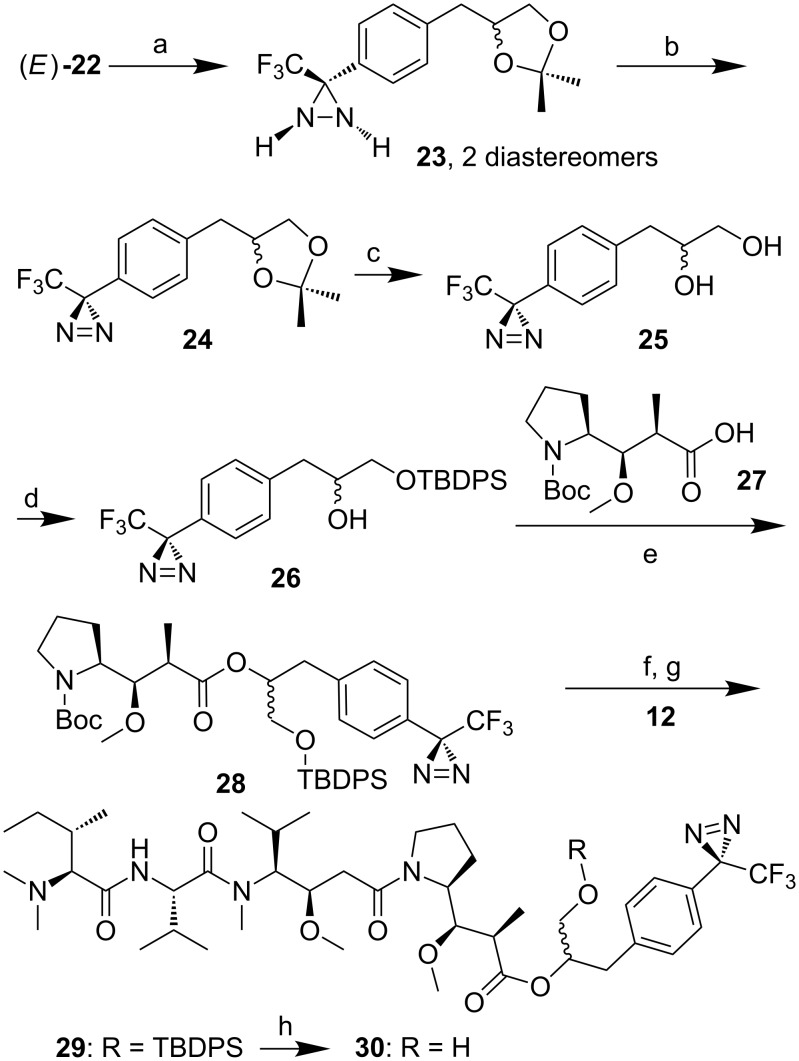
Synthesis of photo malevamide D **30**. a) NH_3_(l), *t*-BuOMe, −40 °C, 2 h, rt, 16 h, quant. b) I_2_ (1.2 equiv), NEt_3_ (2.2 equiv), Et_2_O, 0 °C, 15 min, rt, 1 h, 99%. c) THF, 2 N HCl, rt, 3 h, 97%. d) TBDPSCl (1.1 equiv), imidazole (2.2 equiv), DMF, 0 °C, 1 h, 80%. e) **27** (0.97 equiv), DMAP (0.41 equiv), DCC (1 equiv), CSA (0.23 equiv), 0 °C to rt, 16 h, 69%. f) TFA, DCM, 0 °C, 90 min. g) **12** (1 equiv), NEt_3_ (6 equiv), DEPC (2.14 equiv), DCM, 0 °C, 150 min, 68%. h) TBAF·3H_2_O (5 equiv), THF/H_2_O 19:1, 0 °C, 2 h, 33%.

**Figure 2:**
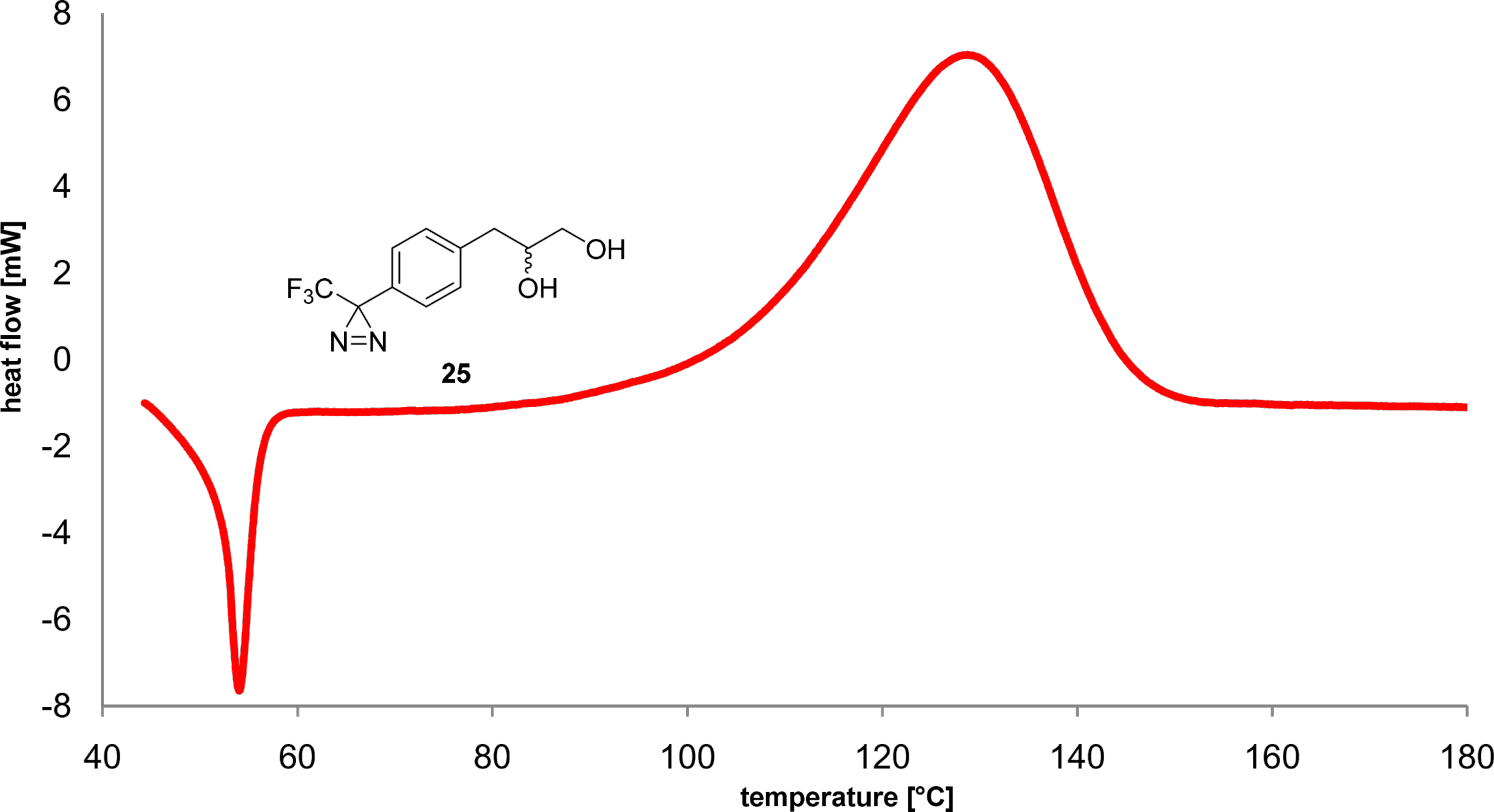
DSC curve of diazirine **25**, heating rate 5 °C/min.

Conversion of (*E*)-**22** to diaziridine **23** (colorless solid, δ_F_ −75.96, −75.93) was performed quantitatively (liquid NH_3_ in *t*-BuOMe, Scheme 4). As expected, the NMR spectra of the colorless solid **23** showed two sets of signals. Oxidation of **23** with I_2_/NEt_3_ to diazirine **24** (δ_F_ −65.7) and acidic deprotection afforded diazirine diol **25**. Figure 2 shows the DSC (differential scanning calorimetry) profile of **25**, which melts at 54 °C and starts to decompose above 80 °C, indicating sufficient thermal stability for biological studies. Monosilylation of the primary hydroxy group of **25** afforded building block **26**. Coupling with Boc-protected dolaproine (**27**, DCC/DMAP/CSA) yielded ester **28** as a mixture of two diastereomers. After deprotection of the pyrrolidine, coupling with tripeptide **12** afforded *O*-silylated diazirinyl-substituted malevamide D (**29**, 68%). Desilylation (TBAF, THF/H_2_O 19:1) led to a product mixture, the separation of which required semipreparative HPLC (RP-18, MeOH/H_2_O 9:1). Diazirinyl-substituted malevamide **30** was obtained in 33% isolated yield as a mixture of two epimers differing at the carbinol center (HRESIMS found 863.48644, calcd. for C_42_H_67_NaF_3_N_6_O_8_, 863.48647). In the NMR spectra, four sets of signals were observed because of additional *cis*/*trans* isomerism of the acyl pyrrolidine moiety. As in the case of **1**, the tripeptidic western half showed mostly broad signals, whereas the signals of the phenyl carbons are resolved better, even if not fully separated.

### Cytotoxicity studies of malevamide D and diazirinyl-substituted malevamide D

The synthesized **1** proved to be potently cytotoxic with a geomean IC_70_ value of 1.5 nM (IC_50_ 0.7 nM) in a panel of 42 human cancer cell lines (see Table 1 and Supporting Information File 1). The cytotoxicity was about 10-fold higher than that of paclitaxel. The breast cancer cell lines MAXF-401 (IC_70_ 0.2 nM) and MCF-7 (0.5 nM), the colon cancer cell lines HT-29 (0.3 nM) and RKO (0.5 nM), the lung cancer cell line H460 (0.7 nM), the liver cancer cell line LIXF-575 (0.6 nM), and the renal cancer cell line RXF-393 (0.4 nM) all proved to be very sensitive. Against 36 of the 42 cell lines, IC_70_ values of below 10 nM were determined. The most resistant cell lines were shown to be RXF-1781 (kidney), CXF-269 (colon), MEXF-276 (melanoma, IC_70_ about 50 nM, each), and in particular LXFA-289 (lung, adenocarcinoma), PAXF-546L (pancreas), and PXF-1118 (pleuramesothelioma, IC_70_ > 100 nM, IC_50_ about 50 nM, each). Diazirinyl-substituted malevamide D **30** was about 200 times less cytotoxic than **1**.

**Table 1:** Cytotoxicities (IC_70_, IC_50_, nM) of malevamide D (**1**) against selected human cancer cell lines.^a^

histotype	cell line	IC_70_	IC_50_

Colon	HT-29	0.3	0.2
Stomach	GXF-251	5.0	0.6
Lung, adeno	LXFA-629	0.7	0.4
Lung, large cell	LXFL-529	0.4	0.3
Breast	MAXF-401	0.2	0.2
Melanoma	MEXF-462	0.6	0.4
Ovary	OVXF-899	1.0	0.7
Pancreas	PAXF-1657	4.0	1.0
Prostate	PRXF-22Rv1	0.8	0.6
Mesothelioma	PXF-1752	0.4	0.3
Kidney	RXF-486L	0.7	0.5
Uterus	UXF-1138L	0.5	0.4

^a^For complete results in the panel of 42 human cancer cell lines and a COMPARE analysis, see Supporting Information File 1.

To obtain clues on the likely mode of action of **1** and **30**, the in vitro activity data were compared with those of 177 reference compounds by COMPARE analysis. Individual IC_50_ values of a test compound were correlated with those of 177 reference compounds as determined for Oncotest's 42 cell line panel and expressed quantitatively as Spearman correlation coefficients (see Supporting Information File 1). Furthermore, **1** and **30** were correlated to each other. For compound **30**, COMPARE analysis revealed the highest similarities towards tubulin interacting compounds like the vinca alkaloids vinfluine, vincristine, vindesine and the taxoid compound docetaxel. Furthermore, good correlations were detected towards PLK-1 inhibitors. Similar results were obtained for **1**. Determining a Spearman coefficient of 0.742 between **1** and **30** suggested that these compounds share the same mode of action, most probably antimitotic activity based on tubulin interaction.

## Conclusion

Our cytotoxicity test results for **1** confirm those obtained by Scheuer and co-workers who determined IC_50_ values of about 0.7 nM against four cell lines (mouse lymphoma P-388, human lung carcinoma A-549, human colon carcinoma HT-29, human melanoma MEL-28) [1]. We had hoped that introduction of a Brunner-type diazirine unit in the *para*-position of the phenyl ring of **1** could lead to an analog that was still active, because it had been shown in several SAR studies that limited variation of the amine or alcohol component at the C-terminal amide or ester moieties of the dolastatin family members can be tolerated without much loss of cytotoxicity. This includes epimerization of the thiazole-carrying stereocenter of **3** [18], replacement of the phenyl by a methyl group, or removal of the thiazole ring [7]. Auristatins, of which some undergo clinical trials, are characterized by lacking the thiazole ring of **3** [19]. Pettit and co-workers prepared auristatin TP, which carries a phosphate unit in the *para*-position of the phenyl ring, and also two aminoquinoline derivatives that were as cytotoxic as dolastatin 10 [3]. Antibody conjugates of monomethylauristatin E functionalized on the C-terminal side have also shown promising pharmacological profiles [20–21]. However, compound **30** is at least 200 times less active than the natural product **1**. Lower cytotoxicity may be associated with solubility problems. We will now search for more water-soluble, photoactivatable analogs of **1**. Nunes and coworkers had synthesized a benzophenone-labelled derivative of the analog HTI-286, which competes with **3**, and were able to identify α-tubulin as binding partner [22].

## Supporting Information

File 1 Procedures of synthesis and biotest, X-ray data and ^1^H, ^13^C NMR spectra of selected compounds.
