# Supplementary material for: Total synthesis and cytotoxicity of the marine natural product malevamide D and a photoreactive analog
Source: Beilstein J Org Chem. 2014 Feb 3;10:316–22. doi: 10.3762/bjoc.10.29 (PMC3943603; doi:10.3762/bjoc.10.29)

Supporting Information

for

Total synthesis and cytotoxicity of the marine natural product malevamide D and a photoreactive analog

Werner Telle[1], Gerhard Kelter[2], Heinz-Herbert Fiebig[2], Peter G. Jones[3] and Thomas Lindel*[1]

Address: [1]Institute of Organic Chemistry, TU Braunschweig, Hagenring 30, 38106 Braunschweig, Germany, [2]Oncotest Institute for Experimental Oncology GmbH, Am Flughafen 12–14, 79108 Freiburg, Germany and [3]Institute of Inorganic and Analytical Chemistry, TU Braunschweig, Hagenring 30, 38106 Braunschweig, Germany

Email: Thomas Lindel - th.lindel@tu-braunschweig.de

*Corresponding author

Procedures of synthesis and biotest, X-ray data and ^{1}H, ^{13}C NMR spectra of selected compounds

1. Synthesis

General: Chemicals were obtained from Aldrich, Merck, Abbott, and ABCR in high quality. NMR spectra were recorded with Bruker DPX-200, AVII 300, AV III-400, DRX-400, and AV II-600 spectrometers. ^{19}F NMR chemical shifts are referenced to Cl_3CF as external standard. Mass spectra were taken on Finnigan MAT 95 XLT or Thermofinnigan MAT95 XL spectrometers (EI) or on an LTQ Orbitrap Velos spectrometer (ESI). GC–MS analyses were performed with an Agilent 6890 gas chromatograph and an Agilent 5975b mass spectrometer. IR spectra were measured with a Bruker Tensor 27 spectrometer equipped with a diamond ATR unit. UV–vis spectra were recorded with a Varian Cary 100 Bio UV–vis spectrometer. Optical rotations were determined with a Dr. Kernchen Propol Automatic polarimeter. Melting points were measured with a Büchi 530 apparatus. A SP Differential calorimeter from Rheometric Scientific was used for DSC measurements.

(*S*)-Benzyl methyl(3-methyl-1-oxobutan-2-yl)carbamate (5). A solution of alcohol **4** (13.310 g, 52.9 mmol, 1.0 equiv) in dry DMSO (60 mL, 845 mmol, 16.0 equiv) was treated under argon with dry NEt_3 (37 mL, 263 mmol, 5.0 equiv) and stirred for 15 min at ambient temperature. After cooling to 0 °C, pyridine·SO_3-complex (41.95 g, 263 mmol, 5.0 equiv) was added and the reddish reaction mixture was stirred for additional 50 min. The reaction was stopped by addition of water (150 mL). The mixture was extracted with TBME (4 × 400 mL). The combined extracts were washed with 25% aqueous citric acid (100 mL), water (100 mL), saturated $NaHCO_3$ solution (100 mL), and brine (100 mL). The Et_2O phase was dried over $MgSO_4$. After filtration and removal of the solvent the product was purified by flash chromatography on silica gel [PE/EtOAc (5:1)] to yield aldehyde **5** (12.26 g, 49.1 mmol, 91%) as a colorless oil. **TLC** [silica gel, PE/EtOAc (5:1)]: R_f = 0.43. $[\alpha]^{30}_D$ –35.5 (*c* 1.45, $CHCl_3$). **^{1}H NMR** (400 MHz, $CDCl_3$, 2 rotamers, ratio 2:1): δ = 9.69 (s, 0.65 H, C*H*O), 9.65 (s, 0.35 H, C*H*O), 7.40-7.27 (m, 5 H, C_6H_5), 5.17 (s, 1.35 H, CH_2), 5.15 (s, 0.65 H, CH_2), 4.16 (d, 0.65 H, ^3J = 9.8 Hz, NC*H*), 3.96 (d, 0.35 H, ^3J = 9.8 Hz, NC*H*), 2.91 (s, 1 H, NCH_3), 2.89 (s, 2 H, NCH_3), 2.34-2.17 (m, 1 H, $CH(CH_3)_2$), 1.13 (d, 2 H, ^3J = 6.6 Hz, $CH(CH_3)_2$), 1.08 (d, 1 H,

^3J = 6.4 Hz, CH(CH_3)$_2$), 0.94 (d, 2 H, ^3J = 6.8 Hz, CH(CH_3)$_2$), 0.90 (d, 1 H, ^3J = 7.0 Hz, CH(CH_3)$_2$). **^{13}C NMR** (100 MHz, $CDCl_3$, major rotamer): δ = 198.9 (1C, *C*HO), 157.0 (1C, N*C*O), 136.4 (1C, PhC_q), 128.4 (2C, *m*Ph-*C*), 128.0 (1C, *p*Ph-*C*), 127.7 (2C, *o*Ph-*C*), 70.8 (1C, N*C*H), 67.5 (1C, *C*H_2), 32.5 (1C, N*C*H_3), 26.5 (1C, *C*H(CH_3)$_2$), 20.0 (1C, CH(*C*H_3)$_2$), 19.4 (1C, CH(*C*H_3)$_2$). **IR** (ATR): $\tilde{\nu}$ = 3034 cm^{-1} (w), 1731 (m), 1693 (s), 1453 (m), 1399 (m), 1367 (m), 1298 (m), 1160 (m), 1131 (m), 769 (m), 736 (m), 697 (s). **UV** (MeOH): λ_{max} (lg ε) = 257 nm (2.36), 207 (3.92). **MS** (EI, 70 eV): *m/z* (%) = 220 $[M\text{-}CHO]^+$ (16), 176 (23), 108 $[BnOH]^+$ (32), 91 $[Bn]^+$ (100), 79 (16), 65 (21), 51 (10). **HRGCMS** (EI, 70 eV): calcd. for $C_{14}H_{19}NO_3$ $[M]^+$ 249.13539; found 249.13711.

(3*S*,4*S*)- and (3*R*,4*S*)-*tert*-Butyl 4-((benzyloxycarbonyl)(methyl)amino)-3-hydroxy-5-methylhexanoate (6). To a solution of LDA, freshly prepared from diisopropylamine (17.5 mL, 123.0 mmol, 2.56 equiv) and *n*-BuLi (2.5 N in *n*-hexane, 48 mL, 120.0 mmol, 2.50 equiv) in dry THF (250 mL) was added at –78 °C *t*-BuOAc (8.5 mL, 62.9 mmol, 1.3 equiv). It was stirred for 2 h, and was allowed to warm to –5 °C. Upon recooling to –78 °C a solution of aldehyde **5** (11.96 g, 48.0 mmol, 1.0 equiv) in dry THF (30 mL) was added slowly. The reaction mixture was stirred at –78 °C for 1 h and quenched with ice cold water (80 mL) followed by water (500 mL). When the ice had melted, the mixture was extracted with Et_2O (2 × 400 mL, 3 × 300 mL). The organic phases were combined and washed with water (100 mL) and brine (100 mL). After drying over $MgSO_4$ and removal of the solvent the residue was purified by double chromatography on silica gel [PE/acetone (20:1)] to yield (3*S*,4*S*)-**6** (6.552 g, 17.93 mmol, 37%) and (3*R*,4*S*)-**6** (8.205 g, 21.9 mmol, 47%) as colorless oils. (3*S*,4*S*)-**6: TLC** [silica gel, PE/acetone (10:1)]: R_f = 0.22. $[\alpha]^{26.8}_D$ –55.2 (*c* 1.45, $CHCl_3$). **^{1}H NMR** (400 MHz, $CDCl_3$, 2 rotamers, ratio 2:1): δ = 7.38-7.27 (m, 5 H, C_6H_5), 5.17 (d, 0.65 H, ^2J = 12.5 Hz, OCH_2), 5.15 (d, 0.35 H, ^2J = 12.3 Hz, OCH_2), 5.13 (d, 0.65 H, ^2J = 12.5 Hz, OCH_2), 5.12 (d, 0.35 H, ^2J = 12.3 Hz, OCH_2), 4.32 (ddd, 0.65 H, ^3J = 3.9 Hz, ^3J = 3.9 Hz, ^3J = 12.4 Hz, C*H*OH), 4.26 (dddd, 0.35 H, ^3J = 3.0 Hz, ^3J = 3.0 Hz, ^3J = 3.0 Hz, ^3J = 9.3 Hz, C*H*OH), 3.80-3.14 (m, 1 H, O*H*), 3.52 (dd, 0.6 H, ^3J = 2.6 Hz, ^3J = 10.6 Hz, NC*H*), 3.34 (d, 0.4

H, ^3J = 2.0 Hz, NC*H*), 2.962 (s, 2 H, NCH_3), 2.958 (s, 1 H, NCH_3), 2.46-2.16 (m, 1 H, C*H*($CH_3)_2$), 2.33 (dd, 1 H, ^3J = 9.6 Hz, ^2J = 17.1 Hz, CH_2COO), 2.25 (dd, 1 H, ^3J = 2.7 Hz, ^2J = 19.7 Hz, CH_2COO), 1.46 (s, 6 H, C(C$H_3)_3$), 1.45 (s, 3 H, C(C$H_3)_3$), 1.06 (d, 2 H, ^3J = 6.5 Hz, CH(C$H_3)_2$), 1.02 (d, 1 H, ^3J = 6.6 Hz, CH(C$H_3)_2$), 0.88 (d, 2 H, ^3J = 6.6 Hz, CH(C$H_3)_2$), 0.83 (d, 1 H, ^3J = 6.7 Hz, CH(C$H_3)_2$). **^{13}C NMR** (100 MHz, $CDCl_3$, major rotamer): δ = 173.0 (1C, CH_2*C*OO), 157.5 (1C, N*C*O), 136.8 (1C, PhC_q), 128.4 (2C, *m*Ph-*C*), 127.8 (1C, *p*Ph-*C*), 127.4 (2C, *o*Ph-*C*), 81.4 (1C, *C*($CH_3)_3$), 67.9 (1C, *C*HOH), 67.1 (1C, OCH_2), 64.3 (1C, N*C*H), 39.4 (1C, *C*H_2COO), 31.0 (1C, NCH_3), 28.0 (3C, C($CH_3)_3$), 25.9 (1C, *C*H($CH_3)_2$), 20.0 (1C, CH($CH_3)_2$), 19.7 (1C, CH($CH_3)_2$). **IR** (ATR): $\tilde{\nu}$ = 3469 cm^{-1} (br, w), 1695 (s), 1678 (s), 1310 (m), 1152 (s), 1111 (m), 751 (m), 735 (m), 697 (m). **UV** (MeOH): λ_{max} (lg ε) = 205 nm (3.98), 257 (2.52). **MS** (EI, 70 eV): *m/z* (%) = 220 [M-CH(OMe)CH_2CO_2*t*Bu]$^+$ (10), 206 (14), 191 (100), 176 (21), 158 (31), 107 (10), 91 [Bn]$^+$ (53), 79 (12), 57 [*t*Bu]$^+$ (41), 43 (13). **MS** (ESI): 388 [M+Na]$^+$ (100), 332 [M-*t*Bu+H+Na]$^+$ (35). **HRMS** (ESI): calcd. for $C_{20}H_{31}NO_5Na$ [M+Na]$^+$ 388.20944; found 388.20869.

(3*R*,4*S*)-**6. TLC** [silica gel, PE/acetone (10:1)]: R_f = 0.18. $[\alpha]^{27}_D$ –5.0 (*c* 1.45, $CHCl_3$). **^{1}H NMR** (400 MHz, $CDCl_3$, 2 rotamers, ratio 3:2): δ = 7.37-7.27 (m, 5 H, C_6H_5), 5.17 (d, 0.4 H, ^2J = 12.4 Hz, OCH_2), 5.16 (d, 0.6 H, ^2J = 12.4 Hz, OCH_2), 5.12 (d, 0.6 H, ^2J = 12.4 Hz, OCH_2), 5.09 (d, 0.4 H, ^2J = 12.4 Hz, OCH_2), 4.30-4.22 (m, 0.6 H, C*H*OH), 4.22-4.11 (m, 0.4 H, C*H*OH), 3.95-3.79 (m, 0.4 H, NC*H*), 3.79-3.60 (m, 0.6 H, NC*H*), 3.51 (brs, 0.6 H, O*H*), 3.32 (d, 0.4 H, ^3J = 3.0 Hz, O*H*), 2.86 (s, 1.8 H, NCH_3), 2.80 (s, 1.2 H, NCH_3), 2.40 (dd, 1 H, ^3J = 2.7 Hz, ^2J = 19.7 Hz, CH_2COO), 2.29 (dd, 1 H, ^3J = 9.5 Hz, ^2J = 16.8 Hz, CH_2COO), 2.25-2.05 (m, 1 H, C*H*($CH_3)_2$), 1.46 (s, 5.4 H, C(C$H_3)_3$), 1.45 (s, 3.6 H, C(C$H_3)_3$), 1.05 (d, 1.8 H, ^3J = 6.7 Hz, CH(C$H_3)_2$), 1.01 (d, 1.2 H, ^3J = 6.8 Hz, CH(C$H_3)_2$), 0.93 (d, 2 H, ^3J = 6.8 Hz, CH(C$H_3)_2$), (d, 1.8 H, ^3J = 6.8 Hz, CH(C$H_3)_2$), 0.92-0.88 (m, 1.2 H, CH(C$H_3)_2$). **^{13}C NMR** (100 MHz, $CDCl_3$, major rotamer): δ = 172.3 (1C, CH_2*C*OO), 157.3 (1C, N*C*O), 136.8 (1C, PhC_q), 128.4 (2C, *m*Ph-*C*), 127.8 (1C, *p*Ph-*C*), 127.5 (2C, *o*Ph-*C*), 81.3 (1C, *C*($CH_3)_3$), 69.4 (1C, *C*HOH), 67.1 (1C, OCH_2), 63.2 (1C, N*C*H), 40.0 (1C, *C*H_2COO), 30.8 (1C, NCH_3), 28.0 (3C,

$C(CH_3)_3$), 27.9 (1C, $CH(CH_3)_2$), 20.5 (1C, $CH(CH_3)_2$), 19.9 (1C, $CH(CH_3)_2$). **IR** (ATR): $\tilde{\nu}$ = 3460 cm^{-1} (br, w), 1305 (m), 1148 (s), 769 (m), 736 (m), 697 (m). **UV** (MeOH): λ_{max} (lg ε) = 205 nm (3.97), 257 (2.40). **MS** (ESI): *m/z* (%) = 753 $[2M+Na]^+$ (49), 388 $[M+Na]^+$ (100). **HRMS** (ESI): calcd. for $C_{20}H_{31}NO_5Na$ $[M+Na]^+$ 388.20944; found 388.20879.

(3*R*,4*S*)-*tert*-Butyl 4-((benzyloxycarbonyl)(methyl)amino)-3-methoxy-5-methyl-hexanoate (7). Under argon, a solution of (3*R*,4*S*)-**6** (1.734 g, 4.74 mmol, 1.0 equiv) in dry 1,2-DCE (60 mL) was treated with powdered MS (4 Å) and stirred for 10 min. 1,8-Bis(dimethylamino)naphthalene (2.541 g, 11.85 mmol, 2.5 equiv) was added. The suspension was cooled to 0 °C and Me_3OBF_4 (1.822 g, 12.32 mmol, 2.6 equiv) was added and the reaction mixture was stirred for 2 h at 0 °C and 16 h at ambient temperature. The suspension was filtered and washed with DCM (2 × 40mL). The solvent was removed in vacuo and the remaining slurry was purified by chromatography on silica gel [PE/EtOAc (4:1)] to yield methyl ether (3*R*,4*S*)-**7** (1.561 g, 4.12 mmol, 87%) as colorless oil. Alcohol (3*R*,4*S*)-**6** (65 mg, 0.18 mmol, 4%) was recovered. **TLC** [silica gel, PE/EtOAc (8:1)]: R_f = 0.58. $[\alpha]^{22}_D$ –17.7 (*c* 1.0, MeOH). **^{1}H NMR** (400 MHz, $CDCl_3$, 2 rotamers, ratio 5:4): δ = 7.37-7.27 (m, 5 H, C_6H_5), 5.17 (d, 0.55 H, ^2J = 12.6 Hz, OCH_2), 5.16 (d, 0.45 H, ^2J = 12.3 Hz, OCH_2), 5.11 (d, 0.45 H, ^2J = 12.3 Hz, OCH_2), 5.10 (d, 0.55 H, ^2J = 12.6 Hz, OCH_2), 4.18-3.78 (m, 2 H, OC*H*C*H*), 3.40 (s, 1.7 H, OCH_3), 3.31 (s, 1.3 H, OCH_3), 2.81 (s, 1.7 H, NCH_3), 2.80 (s, 1.3 H, NCH_3), 2.49-2.30 (m, 2 H, CH_2COO), 2.08-2.01 (m, 1 H, $CH(CH_3)_2$), 1.45 (s, 5 H, $C(CH_3)_3$), 1.44 (s, 4 H, $C(CH_3)_3$), 1.02 (d, 1.7 H, ^3J = 6.7 Hz, $CH(CH_3)_2$), 0.97 (d, 1.3 H, ^3J = 6.7 Hz, $CH(CH_3)_2$), 0.92, (d, 1.7 H, ^3J = 6.8 Hz, $CH(CH_3)_2$), 0.90 (d, 1.3 H, ^3J = 6.7 Hz, $CH(CH_3)_2$). **^{13}C NMR** (100 MHz, $CDCl_3$, major rotamer): δ = 171.2 (1C, CH_2COO), 157.2 (1C, NCO), 137.0 (1C, PhC_q), 128.4 (2C, *m*Ph-*C*), 127.9 (1C, *p*Ph-*C*), 127.5 (2C, *o*Ph-*C*), 80.6 (1C, $C(CH_3)_3$), 78.5 (1C, $CHOCH_3$), 67.0 (1C, OCH_2), 62.1 (1C, NCH), 57.8 (1C, OCH_3), 38.6 (1C, CH_2COO), 28.0 (3C, $C(CH_3)_3$), 27.8 (1C, $CH(CH_3)_2$), 20.2 (1C, $CH(CH_3)_2$), 20.0 (1C, $CH(CH_3)_2$). **IR** (ATR): $\tilde{\nu}$ = 2973 cm^{-1} (w), 1696 (s), 1306 (m), 1148 (s), 1100 (m), 698 (m). **UV** (MeOH): λ_{max} (lg ε) = 205 nm (3.96), 257 (2.34). **GC-MS** (EI, 70

eV): m/z (%) = 323 [M-^{t}Bu+H]$^+$ (1), 306 [M–tBuO]$^+$ (1), 280 (2), 262 (2), 236 (9), 220 [M-CH(OMe)$CH_2CO_2{}^t$Bu]$^+$ (34), 128 (12), 91 [$C_6H_5CH_2$] (100).

(3*R*,4*S*)-*tert*-Butyl 4-((*S*)-2-(benzyloxycarbonylamino)-*N*,3-dimethylbutanamido)-3-methoxy-5-methylhexanoate (9) [1]**.** Solution A: Pd/C (5%, 600 mg) was added to a solution of Cbz-MMMAH-O*t*-Bu (**7**, 605 mg, 1.6 mmol, 1.0 equiv) in MeOH (10 mL). The suspension was stirred under hydrogen atmosphere at ambient temperature for 1 h. The suspension was filtered over a Celite pad followed by washing with MeOH. The washings and the filtrate were combined and the solvent was removed under vacuum. The remaining yellow oil was dissolved in dry DCM (5 mL). Solution B: To a solution of Cbz-Valine (1.205 g, 4.8 mmol, 3.0 equiv) in DCM (10 mL) of NEt_3 (0.9 mL, 4.0 equiv) was added. The solution was cooled to 0 °C and DEPCl (0.46 mL, 2.0 equiv) was added dropwise. Solution A was added. The reaction mixture was stirred for 2 h at 0 °C and 16 h at ambient temperature. The reaction mixture was diluted with DCM and washed with 2 N HCl, water, saturated bicarbonate solution, and brine. After evaporation of the solvent the crude peptide was purified by column chromatography (silica, PE/EtOAc: 10:1) to yield product **9** as a colorless oil (610 mg, 1.28 mmol, 80%). **TLC** [PE/EtOAc (3:1)]: R_f = 0.40. ; $[\alpha]^{24}_D$ –15.0 (c 0.7, $CHCl_3$). (lit. : $[\alpha]^{27}_D$ –32.190 (c 1.0, MeOH). **^{1}H NMR** (400 MHz, $CDCl_3$, 2 rotamers, ratio 5:1): δ = 7.37-7.26 (m, 5 H, C_6H_5), 5.66 (d, 0.15 H, ^3J = 9.0 Hz, NH), 5.54 (d, 0.85 H, ^3J = 9.2 Hz, NH), 5.11 (d, 0.15 H, ^2J = 12.4 Hz, OCH_2), 5.09 (s, 1.7 H, OCH_2), 5.04 (d, 0.15 H, ^2J = 12.4 Hz, OCH_2), 4.73 (dd, 0.15 H, ^3J = 9.0 Hz, ^3J = 4.7 Hz, NHCH), 4.70-4.55 (m, 1 H, CH_3NCH), 4.51 (dd, 0.85 H, ^3J = 5.9 Hz, ^3J = 9.2 Hz, NHCH), 3.98-3.92 (m, 0.15 H, OCH), 3.92-3.81 (m, 0.85 H, OCH), 3.35 (s, 2.55 H, OCH_3), 3.34 (s, 0.45 H, OCH_3), 2.98 (s, 2.55 H, NCH_3), 2.78 (s, 0.45 H, NCH_3), 2.44 (dd, 1 H, ^2J = 15.7 Hz, ^3J = 2.0 Hz, CH_2COO), 2.23 (dd, 1 H, ^2J = 15.7 Hz, ^3J = 9.0 Hz, CH_2COO), 2.07-1.81 (m, 2 H, NHCHC$H(CH_3)_2$, CH_3NCHC$H(CH_3)_2$), 1.46 (s, 7.65 H, $C(CH_3)_3$), 1.45 (s, 1.35 H, $C(CH_3)_3$), 1.007 (d, 3 H, ^3J = 6.6 Hz, NHCHCH$(CH_3)_2$), 1.003 (d, 3 H, ^3J = 6.6 Hz, CH_3NCHCH$(CH_3)_2$), 0.92 (d, 3 H, ^3J = 6.8 Hz, NHCHCH$(CH_3)_2$), 0.84 (d, 3 H, ^3J = 6.4 Hz, CH_3NCHCH$(CH_3)_2$). **^{13}C NMR** (100

MHz, $CDCl_3$, major rotamer): δ = 173.2 (1C, CH_3NCO), 171.0 (1C, CH_2COO), 156.4 (1C, NH*C*O), 136.5 (1C, PhC_q), 128.4 (2C, *m*Ph-*C*), 127.9 (1C, *p*Ph-*C*), 127.8 (2C, *o*Ph-*C*), 80.8 (1C, $C(CH_3)_3$), 78.2 (1C, O*C*H), 66.7 (1C, OCH_2), 58.3 (1C, CH_3NCH), 57.7 (1C, OCH_3), 56.0 (1C, NH*C*H), 38.5 (1C, CH_2COO), 31.5 (1C, NCH_3), 31.0 (1C, NHCH*C*H$(CH_3)_2$), 28.0 (3C, $C(CH_3)_3$), 27.0 (1C, $CH_3NCHCH(CH_3)_2$), 20.02 (1C, $CH_3NCHCH(CH_3)_2$), 19.96 (1C, $CH_3NCHCH(CH_3)_2$), 19.6 (1C, NHCHCH$(CH_3)_2$), 17.1 (1C, NHCHCH$(CH_3)_2$). **IR** (ATR): $\tilde{\nu}$ = 3249 cm^{-1} (w), 2967 (m), 1721 (s), 1636 (s), 1524 (m), 1499 (m), 1455 (m), 1367 (m), 1298 (m), 1258 (m), 1233 (m), 1149 (s), 1097 (s), 1024 (m), 737 (m), 697 (m). **UV** (MeOH): λ_{max} (lg ε) = 205 nm (4.17), 257 (2.38). **MS** (ESI): *m/z* (%) = 979 $[2M+Na]^+$ (96), 501 $[M+Na]^+$ (100), 479 $[M+H]^+$ (20). **HRMS** (ESI): calcd. for $C_{26}H_{42}N_2O_5Na$ $[M+Na]^+$ 501.29351; found 501.29350.

(3*R*,4*S*)-*tert*-Butyl 4-((*S*)-2-((2*S*,3*S*)-2-(dimethylamino)-3-methylpentanamido)-*N*,3-dimethylbutanamido)-3-methoxy-5-methylhexanoate (11). Solution A: Pd/C (5%, 220 mg) was added to a solution of dipeptide **9** (213 mg, 0.44 mmol, 1.0 equiv) in MeOH (5 mL). The suspension was stirred under hydrogen atmosphere at ambient temperature for 1.5 h. The suspension was filtered over a Celite pad followed by washing with MeOH. The washings and the filtrate were combined and the solvent was removed under vacuum. The remaining yellow oil was dissolved in dry DCM (2 mL). Solution B: A suspension of *N*,*N*-dimethylisoleucine (210 mg, 1.32 mmol, 3.0 equiv) in DCM (5 mL) was treated with NEt_3 (0.25 mL, 4.0 equiv). The clear solution was cooled to 0 °C and DEPC (100 µL, 2.0 equiv) was added followed by 10 min of stirring. Solution A was added dropwise. The reaction mixture was stirred for 2 h at 0 °C and 16 h at ambient temperature. The reaction mixture was diluted with DCM and extracted with 25% citric acid. The aqueous solution was treated with bicarbonate and extracted three times with chloroform. The combined extracts were washed with brine. The solvent was removed to yield tripeptide **11** (213 mg, 0.37 mmol, 83%) as a colorless oil. **TLC** (EtOAc): R_f = 0.35. $[\alpha]^{28}_D$ –20.0 (*c* 0.15, MeOH). **^{1}H NMR** (400 MHz, $CDCl_3$): δ = 6.99 (d, 1 H, ^3J = 9.1 Hz, N*H*), 4.77 (dd, 1 H, ^3J = 9.1 Hz, ^3J = 6.6 Hz,

C*H*NH), 4.73-4.55 (m, 1 H, CH_3NCH), 3.98-3.78 (m, 1 H, CH_3OCH), 3.36 (s, 3 H, OCH_3), 3.02 (s, 3 H, CH_3NCH), 2.54 (d, 1 H, ^3J = 5.9 Hz, $CHN(CH_3)_2$), 2.45 (dd, 1 H, ^2J = 15.4 Hz, ^3J = 1.8 Hz, CH_2CO), 2.31 (dd, 1 H, ^2J = 15.4 Hz, ^3J = 9.6 Hz, CH_2CO), 2.22 (s, 6 H, $N(CH_3)_2$), 2.10-1.96 (m, 1 H, $NHCHCH(CH_3)_2$), 1.96-1.74 (m, 2 H, $CH_3NCHCH(CH_3)_2$, CH_3CH_2CH), 1.62-1.50 (m, 1 H, CH_3CH_2), 1.46 (s, 9 H, $C(CH_3)_3$), 1.28-1.12 (m, 1 H, CH_3CH_2), 1.02 (d, 3 H, ^3J = 6.8 Hz, $NHCHCH(CH_3)_2$), 0.99 (d, 3 H, ^3J = 6.6 Hz, $CH_3NCHCH(CH_3)_2$), 0.96 (d, 3 H, ^3J = 6.7 Hz, $NHCHCH(CH_3)_2$), 0.93 (t, 3 H, ^3J = 7.4 Hz, CH_3CH_2), 0.91 (d, 3 H, ^3J = 6.7 Hz, $CH_3CH_2CHCH_3$), 0.81 (d, 3 H, ^3J = 6.6 Hz, $CH_3NCHCH(CH_3)_2$). **^{13}C NMR** (100 MHz, $CDCl_3$): δ = 173.2 (1C, CH_3NCO), 171.9 (1C, NHCO), 171.0 (1C, COO), 80.8 (1C, $C(CH_3)_3$), 78.1 (1C, OCH), 74.8 (1C, $(CH_3)_2NCH$), 58.2 (1C, CH_3NCH), 57.8 (1C, OCH_3), 53.7 (1C, NHCH), 43.0 (2C, $(CH_3)_2N$), 38.5 (1C, CH_2COO), 34.4 (1C, $CH_3CH_2CHCH_3$), 31.7 (1C, NCH_3), (30.9 (1C, NHCHCH), 28.0 (3C, $C(CH_3)_3$), 26.90 (1C, CH_3NCHCH), 26.87 (1C, CH_3CH_2), 20.0 (1C, $CH_3NCHCH(CH_3)_2$), 19.9 (1C, $CH_3NCHCH(CH_3)_2$), 19.7 (1C, $NHCHCH(CH_3)_2$), 18.1 (1C, $NHCHCH(CH_3)_2$), 14.7 (1C, $CH_3CH_2CHCH_3$), 11.9 (1C, CH_3CH_2). **IR** (ATR): $\tilde{\nu}$ = 3297 cm^{-1} (w), 2963 (m), 2928 (m), 1730 (m), 1622 (s), 1368 (m), 1151 (s), 1099 (m). **UV** (MeOH): λ_{max} (lg ε) = 202 nm (4.20). **MS** (ESI): *m/z* (%) = 993 $[2M+Na]^+$ (25), 971 $[2M+H]^+$ (8), 508 $[M+Na]^+$ (48), 486 $[M+H]^+$ (100). **HRMS** (ESI): calcd. for $C_{26}H_{32}N_3O_5$ $[M+H]^+$ 486.39015; found 486.39041.

(2*S*,3*S*)-1-((*S*)-1-(((2*R*,3*S*)-1-Carboxy-2-methoxy-4-methylpentan-3-yl)(methyl)amino)-3-methyl-1-oxobutan-2-ylamino)-*N*,*N*,3-trimethyl-1-oxopentan-2-aminium 2,2,2-trifluoroacetate (12). A solution of peptide **11** (80 mg, 0.164 mmol, 1.0 equiv) in DCM/trifluoroacetic acid (4 mL, 1:1) was stirred at 0 °C 1.5 h. The solvent was removed and the residue was solved in DCM (50 mL) and evaporated to dryness. Acetate **12** (89 mg, 0.164 mmol, quant.) was obtained as a yellow oil. **TLC** (silica gel RP-18, MeOH): R_f = 0.84. $[\alpha]^{22}_D$ –127.0 (*c* 1.59, $CHCl_3$). **^{1}H NMR** (600 MHz, $CDCl_3$): δ = 9.54 (brs, 1 H, CH_2CO_2H), 6.30 (brs, 2 H, CHN *H,* $(CH_3)_2NH^+$), 4.91 (dd, 1 H, ^3J = 9.6 Hz, ^3J = 9.6 Hz, C*H*NH), 4.59 (dd, 1 H, ^3J = 3.6 Hz, ^3J = 8.7 Hz, CH_3NCH), 4.12 (d, 1 H, ^3J = 10.3 Hz, $(CH_3)_2NH^+CH$), 3.56-3.51 (m,

1 H, C*H*OCH$_3$), 3.37 (s, 3 H, OC*H*$_3$), 3.17 (s, 3 H, (C*H*$_3$)$_2$NH$^+$), 3.10 (s, 3 H, NC*H*$_3$), 2.90 (s, 3 H, (C*H*$_3$)$_2$NH$^+$), 2.70 (dd, 1 H, ^3J = 9.4 Hz, ^2J = 16.3 Hz, C*H*$_2$CO$_2$H), 2.59 (dd, 1 H, ^3J = 9.4 Hz, ^2J = 16.3 Hz, C*H*$_2$CO$_2$H), 2.19-2.10 (m, 1 H, CH$_3$NCHC*H*(CH$_3$)$_2$), 2.07-1.97 (m, 2 H, NHCHC *H,* CH$_3$CH$_2$C*H*), 1.75-1.67 (m, 1 H, CH$_3$C*H*$_2$), 1.13-1.02 (m, 1 H, CH$_3$C*H*$_2$), 1.02 (d, 3 H, ^3J = 7.1 Hz, NHCHCH(C*H*$_3$)$_2$), 1.01 (d, 3 H, ^3J = 7.1 Hz, NHCHCH(C*H*$_3$)$_2$), 0.97 (d, 3 H, ^3J = 6.7 Hz, CH$_3$NCHCH(C*H*$_3$)$_2$), 0.95 (t, 3 H, ^3J = 7.3 Hz, C*H*$_3$CH$_2$), 0.90 (d, 3 H, ^3J = 6.6 Hz, CH$_2$CHC*H*$_3$), 0.72 (d, 3 H, ^3J = 6.8 Hz, CH$_3$NCHCH(C*H*$_3$)$_2$). **^{13}C NMR** (150 MHz, CDCl$_3$): δ = 173.9 (1C, CH$_2$*C*O$_2$H), 172.4 (1C, CH$_3$N*C*O), 167.0 (1C, NH*C*O), 161.5 (q, 1C, $^2J_{CF}$ = 39.4 Hz, CF$_3$*C*O), 116.1 (q, 1C, $^1J_{CF}$ = 288.0 Hz, *C*F$_3$), 79.8 (1C, *C*HOCH$_3$), 70.5 (1C, (CH$_3$)$_2$NH$^+$*C*H), 59.9 (1C, CH$_3$N*C*H), 56.9 (1C, O*C*H$_3$), 54.4 (1C, NH*C*H), 43.3 (1C, (*C*H$_3$)$_2$NH$^+$), 38.0 (1C, (*C*H$_3$)$_2$NH$^+$), 34.9 (1C, *C*H$_2$CO), 34.1 (1C, CH$_3$CH$_2$*C*H), 32.5 (1C, NHCH*C*H(CH$_3$)$_2$), 31.8 (1C, N*C*H$_3$), 27.1 (1C, CH$_3$NCH*C*H(CH$_3$)$_2$), 24.3 (1C, CH$_3$*C*H$_2$), 20.9 (1C, CH$_3$NCHCH(*C*H$_3$)$_2$), 19.9 (1C, CH$_3$NCHCH(*C*H$_3$)$_2$), 18.7 (1C, NHCHCH(*C*H$_3$)$_2$), 18.6 (1C, NHCHCH(*C*H$_3$)$_2$), 15.4 (1C, CH$_2$CH*C*H$_3$), 11.2 (1C, CH$_2$*C*H$_3$). **^{19}F NMR** (376 MHz, CDCl$_3$): δ = –76.17 (s, 3F, C*F*$_3$). **IR** (ATR): $\tilde{\nu}$ = 3429 cm^{-1} (w), 1669 (s), 1632 (m), 1176 (s), 1132 (s), 1099 (m), 910 (m), 799 (m), 722 (s). **UV** (MeOH): λ_{max} (lg ε) = 203 nm (4.06). **MS** (ESI): *m/z* (%) = 881 [2M+Na]$^+$ (11), 452 [M+Na]$^+$ (32), 430 [M+H]$^+$ (100). **HRMS** (ESI): calcd. for $C_{22}H_{43}N_3O_5Na$ [M+Na]$^+$ 452.30949; found 452.30952.

(*S*)-2-((3*R*,4*R*,7*S*)-7-Benzyl-4,11,11-trimethyl-5-oxo-10,10-diphenyl-2,6,9-trioxa-10-siladodecan-3-yl)pyrrolidinium 2,2,2-trifluoroacetate (13). A solution of *N*-Boc-Dap-PPD-O1-TBDPS [2] (59 mg, 0.09 mmol, 1.0 equiv) in DCM/trifluoroacetic acid (4 mL, 1:1) was stirred at 0 °C 1 h. The solvent was removed and the residue was dissolved in DCM (10 mL) and evaporated to dryness. This procedure was repeated another two times. Acetate **13** (60 mg, 0.09 mmol, quant.) was obtained as a yellow oil. **TLC** (EtOAc): R_f = 0.38 (free amine). $[\alpha]_D^{27}$ –42.3 (*c* 0.15, CHCl$_3$). **^{1}H NMR** (600 MHz, CDCl$_3$): δ = 10.15 (brs, 1 H, N*H*$_2^+$), 8.20 (brs, 1 H, N*H*$_2^+$), 7.64 (dd, 4 H, ^3J = 7.9 Hz, ^3J = 7.9 Hz, *m*Ph-*H*(Si)), 7.43-7.41 (m, 2 H, *p*Ph-*H*(Si)), 7.39-7.35 (m, 4 H, *o*Ph-*H*(Si)), 7.27-7.23 (m, 2 H, *m*Ph-*H*(PPD)), 7.21-7.16 (m, 3

H, *o*Ph-*H*(PPD), *p*Ph-*H*(PPD)), 5.27-5.20 (m, 1 H, OCH_2CH), 3.76-3.68 (m, 1 H, CH_3OCH), 3.74 (dd, 1 H, ^3J = 5.2 Hz, ^2J = 11.0 Hz, OCH_2), 3.71 (dd, 1 H, ^3J = 4.1 Hz, ^2J = 11.0 Hz, OCH_2), 3.49-3.42 (m, 1 H, NC*H*), 3.36 (s, 3 H, OCH_3), 3.23-3.12 (m, 2 H, NCH_2), 3.00 (dd, 1 H, ^3J = 5.5 Hz, ^2J = 14.0 Hz, $PhCH_2$), 2.86 (dd, 1 H, ^3J = 5.5 Hz, ^2J = 14.0 Hz, $PhCH_2$), 2.62-2.56 (m, 1 H, $CHCH_3$), 1.94-1.85 (m, 1 H, NCH_2CH_2), 1.81-1.72 (m, 1 H, NCH_2CH_2), 1.72-1.60 (m, 2 H, $NCHCH_2$), 1.15 (d, 3 H, ^3J = 7.1 Hz, $CHCH_3$), 1.06 (s, 9 H, $C(CH_3)_3$). **^{13}C NMR** (150 MHz, $CDCl_3$): δ = 173.1 (1C, CH*C*OO), 162.0 (q, 1C, $^2J_{CF}$ = 37.5 Hz, F_3CCOO), 137.1 (1C, PhC_q(PPD)), 135.5 (4C, *m*Ph-*C*(Si)), 133.1 (2C, PhC_q(Si)), 129.8 (2C, *p*Ph-*C*(Si)), 129.3 (2C, *o*Ph-*C*(PPD)), 128.4 (2C, *m*Ph-*C*(PPD)), 127.7 (4C, *o*Ph-*C*(Si)), 126.6 (1C, *p*Ph-*C*(PPD)), 116.3 (q, 1C, $^1J_{CF}$ = 282.1 Hz, CF_3), 79.7 (1C, $CHOCH_3$), 75.5 (1C, OCH_2CH), 64.3 (1C, OCH_2), 60.8 (1C, NCH), 59.8 (1C, OCH_3), 45.2 (1C, NCH_2), 41.7 (1C, $CHCH_3$), 36.7 (1C, $PhCH_2$), 26.8 (3C, $C(CH_3)_3$), 24.0 (1C, $NCHCH_2$), 23.9 (1C, NCH_2CH_2), 19.3 (1C, $C(CH_3)_3$), 13.1 (1C, $CHCH_3$). **^{19}F NMR** (376 MHz, $CDCl_3$): δ = –76.07 (s, 3F, CF_3). **IR** (ATR): $\tilde{\nu}$ = 2934 cm^{-1} (w), 1672 (s), 1199 (s), 1177 (s), 1131 (s), 1111 (s), 825 (m), 798 (m), 741 (m), 721 (m), 701 (s). **UV** (MeOH): λ_{max} (lg ε) = 205 nm (4.43), 259 (2.94), 264 (2.92), 289 (2.31). **MS** (ESI): *m/z* (%) = 560 $[M-CF_3COO]^+$ (100). **HRMS** (ESI): calcd. for $C_{34}H_{46}NO_4Si$ $[M+H]^+$ 560.31906; found 560.31913.

(2*R*,3*R*)-((*S*)-1-(*tert*-Butyldiphenylsilyloxy)-3-phenylpropan-2-yl) 3-((*S*)-1-(diethoxyphosphoryl)pyrrolidin-2-yl)-3-methoxy-2-methylpropanoate (31) and (2*R*,3*R*)-((*S*)-1-(*tert*-butyldiphenylsilyloxy)-3-phenylpropan-2-yl) 3-((*S*)-1-((3*R*,4*S*)-4-((*S*)-2-((2*S*,3*S*)-2-(dimethylamino)-3-methylpentanamido)-*N*,3-dimethylbutanamido)-3-methoxy-5-methylhexanoyl)pyrrolidin-2-yl)-3-methoxy-2-methylpropanoate (14). Acetates **12** (54 mg, 0.1 mmol, 1.0 equiv) and **13** (67 mg, 0.1 mmol, 1.0 equiv) were dissolved in DCM (6 mL). The solution was treated with NEt_3 (85 µL, 0.6 mmol, 6 equiv) and cooled to 0 °C. DEPC (20 µL, 0.13 mmol, 1.3 equiv) was added and the reaction mixture was stirred for 16 h with warming to ambient temperature. The reaction mixture was diluted with DCM to 50 mL and washed successively with sat. bicarbonate, water, and brine. Evaporation

of the solvent yielded an oil which was subjected to reverse phase column chromatography (RP-18, MeOH). Phosphonate **31** (6 mg, 0.008 mmol, 8%) eluted first followed by peptide **14** (67 mg, 0.068 mmol, 68%). Both compounds were isolated as colorless oils. **31**: **TLC** (silica gel RP-18, MeOH): R_f = 0.76. $[\alpha]^{22}_D$ –4.3 (*c* 0.28, $CHCl_3$). **^{1}H NMR** (600 MHz, $CDCl_3$): δ = 7.67-7.61 (m, 4 H, *m*Ph-*H*(Si)), 7.45-7.40 (m, 2 H, *p*Ph-*H*(Si)), 7.40-7.34 (m, 4 H, *o*Ph-*H*(Si)), 7.26-7.16 (m, 5 H, $CH_2C_6H_5$), 5.17 (dddd, 1 H, ^3J = 4.6 Hz, ^3J = 5.6 Hz, ^3J = 6.1 Hz, ^3J = 7.7 Hz, OCH_2CH), 4.05–3.90 (m, 4 H, $P(OCH_2CH_3)_2$), 3.73 (dd, 1 H, ^3J = 4.7 Hz, ^2J = 10.9 Hz, OCH_2CH), 3.68 (dd, 1 H, ^3J = 4.5 Hz, ^2J = 10.9 Hz, OCH_2CH), 3.56–3.50 (m, 1 H, NC*H*), 3.49 (dd, 1H ^3J = 5.4 Hz, ^3J = 5.4 Hz, $CHOCH_3$), 3.37–3.28 (m, 1 H, NCH_2), 3.27 (s, 3 H, OCH_3), 3.06 (dd, 1 H, ^3J = 6.2 Hz, ^2J = 13.8 Hz, $PhCH_2$), 3.07–2.99 (m, 1 H, NCH_2), 2.91 (dd, 1 H, ^3J = 7.7 Hz, ^2J = 13.8 Hz, $PhCH_2$), 2.67–2.61 (m, 1 H, C*H*COO), 1.89–1.80 (m, 2 H, $NCHCH_2CH_2$), 1.75–1.67 (m, 2 H, $NCHCH_2CH_2$), 1.27 (dt, 3 H, $^4J_{HP}$ = 0.7 Hz, ^3J = 7.1 Hz, $P(OCH_2CH_3)_2$), 1.24 (dt, 3 H, $^4J_{HP}$ = 0.7 Hz, ^3J = 7.1 Hz, $P(OCH_2CH_3)_2$), 1.15 (d, 3 H, ^3J = 7.0 Hz, $CHCH_3$), 1.07 (s, 9 H, $C(CH_3)_3$). **^{13}C NMR** (150 MHz, $CDCl_3$): δ = 174.9 (1C, *C*OO), 137.4 (1C, PhC_q(PPD)), 135.6 (4C, *m*Ph-*C*(Si)), 133.3 (2C, PhC_q(Si)), 129.7 (2C, *p*Ph-*C*(Si)), 129.4 (2C, *o*Ph-*C*(PPD)), 128.4 (2C, *m*Ph-*C*(PPD)), 127.7 (4C, *o*Ph-*C*(Si)), 126.4 (1C, *p*Ph-*C*(PPD)), 83.7 (d, 1C, $^3J_{CP}$ = 3.3 Hz, $CHOCH_3$), 75.1 (1C, $OCHCH_2O$), 63.9 (1C, $OCHCH_2O$), 62.3 (d, 1C, $^2J_{CP}$ = 5.9 Hz, $P(OCH_2CH_3)_2$), 62.1 (d, 1C, $^2J_{CP}$ = 5.7 Hz, $P(OCH_2CH_3)_2$), 60.4 (d, 1C, $^2J_{CP}$ = 3.6 Hz, N*C*H), 60.3 (1C, OCH_3), 47.2 (d, 1C, $^2J_{CP}$ = 3.3 Hz, NCH_2), 41.8 (1C, $CHCH_3$), 36.7 (1C, $PhCH_2$), 26.79 (d, 1C, $^3J_{CP}$ = 6.2 Hz, $NCHCH_2$), 26.77 (3C, $C(CH_3)_3$), 25.6 (d, 1C, ^3J = 5.6 Hz, NCH_2CH_2), 19.3 (1C, $C(CH_3)_3$), 16.3 (1C, $^3J_{CP}$ = 6.7 Hz, $P(OCH_2CH_3)_2$), 16.2 (1C, $^3J_{CP}$ = 6.7 Hz, $P(OCH_2CH_3)_2$), 11.8 (1C, $CHCH_3$). **^{31}P NMR** (162 MHz, $CDCl_3$): δ = 9.42 (s, 1P, NPO_3) **IR** (ATR): $\tilde{\nu}$ = 3029 cm^{-1} (w), 1730 (m), 1259 (m), 1105 (m), 1027 (s), 959 (m), 793 (m), 742 (m), 701 (s), 588 (m). **UV** (MeOH): λ_{max} (lg ε) = 206 nm (4.44), 253 (2.98), 259 (3.04), 264 (3.03). **MS** (ESI): *m/z* (%) = 1413 $[2M+Na]^+$ (67), 718 $[M+Na]^+$ (100), 696 $[M+H]^+$ (43). **HRMS** (ESI): calcd. for $C_{38}H_{54}NO_7PSiNa$ $[M+Na]^+$ 718.32994; found 718.33026.

14: TLC [silica gel RP-18, MeOH]: R_f = 0.30. $[\alpha]^{24}_D$ –24.4 (*c* 0.16, $CHCl_3$). **^{1}H NMR** (600 MHz, $CDCl_3$, 2 conformers, ratio 3:1): δ = 7.69-7.61 (m, 4 H, *m*Ph-*H*(Si)), 7.47-7.33 (m, 6 H, *o*Ph-*H*(Si), *p*Ph-*H*(Si)), 7.26-7.14 (m, 5 H, $CH_2C_6H_5$), 7.05-6.95 (m, 1 H, N*H*), 5.26 (ddd, 0.25 H, ^3J = 5.3 Hz, ^3J = 9.0 Hz, ^3J = 9.0 Hz, OCH_2CHO), 5.20 (ddd, 0.75 H, ^3J = 4.5 Hz, ^3J = 7.1 Hz, ^3J = 11.4 Hz, OCH_2CHO), 4.83 (dd, 0.25 H, ^3J = 6.7 Hz, ^3J = 9.2 Hz, NHC*H*), 4.79-4.75 (m, 1 H, NHC *H,* CH_3NCH), 4.72-4.60 (m, 0.75 H, CH_3NCH), 4.20-4.08 (m, 1 H, $CH_3NCHCHO$), 3.98 (ddd, 1 H, ^3J = 3.8 Hz, ^3J = 3.8 Hz, ^3J = 7.6 Hz, CH_2NCH), 3.92 (dd, 1 H, ^3J = 3.3 Hz, ^3J = 7.3 Hz, $CH_2NCHCHO$), 3.80-3.60 (m, 3 H, OCH_2, NCH_2), 3.47-3.24 (m, 7 H, NCH_2, $CH_3NCHCHOCH_3$, $CH_2NCHCHOCH_3$), 3.08-2.99 (m, 3 H, NCH_3), 2.97-2.76 (m, 2 H, $PhCH_2$), 2.57-2.50 (m, 1 H, CH_3NCH), 2.50-2.36 (m, 2 H, CH_3OCHCH_2, CH_3CHCOO), 2.31-2.16 (m, 7 H, $(CH_3)_2NC$ H, CH_3OCHCH_2), 2.13-1.77 (m, 5 H, $NCHCH_2CH_2$, $CH_3NCHCH(CH_3)_2$, $NHCHCH(CH_3)_2$, CH_3CH_2CH), 1.73-1.43 (m, 3 H, $NCHCH_2CH_2$, CH_3CH_2), 1.26-1.15 (m, 4 H, CH_3CH_2, CH_3CHCOO), 1.09-1.04 (m, 9 H, $C(CH_3)_3$), 1.04-0.88 (m, 15 H, $CH_3CH_2CHCH_3$, $NHCHCH(CH_3)_2$, $CH_3NCHCH(CH_3)_2$), 0.85-0.81 (m, 3 H, $CH_3NCHCH(CH_3)_2$). **^{13}C NMR** (150 MHz, $CDCl_3$, main conformer): δ = 174.0 (1C, *C*OO), 173.7 (1C, CH_2NCO), 173.3 (1C, CH_3NCO), 172.0 (1C, NH*C*O), 137.3 (1C, PhC_q(PPD)), 135.5 (4C, *m*Ph-*C*(Si)), 133.3 (2C, PhC_q(Si)), 129.7 (2C, *p*Ph-*C*(Si)), 129.1 (2C, *o*Ph-*C*(PPD)), 128.3 (2C, *m*Ph-*C*(PPD)), 127.7 (4C, *o*Ph-*C*(Si)), 126.4 (1C, *p*Ph-*C*(PPD)), 81.5 (1C, $CH_2NCHCHO$), 78.0 (1C, $CH_3NCHCHO$), 75.0 (1C, OCH_2CHO), 74.9 (1C, $(CH_3)_2NCH$), 63.9 (1C, OCH_2CHO), 61.6 (1C, $CH_2NCHCHOCH_3$), 60.4 (1C, $CH_3NCHCHOCH_3$), 59.1 (1C, CH_2NCH), 57.9 (1C, CH_3NCH), 53.7 (1C, NH*C*H), 47.2 (1C, NCH_2), 43.1 (2C, $(CH_3)_2N$), 42.7 (1C, CH_3CHCOO), 37.8 (1C, CH_2CO), 36.6 (1C, $PhCH_2$), 34.4 (1C, CH_3CHCH_2), 31.9 (CH_3NCH), 27.1 (1C, $CH_3NCHCH(CH_3)_2$), 26.9 (1C, CH_3CH_2), 26.8 (3C, $C(CH_3)_3$), 25.0 (1C, $NCHCH_2$), 24.7 (1C, NCH_2CH_2), 20.04 (1C, $CH_3NCHCH(CH_3)_2$), 20.02 (1C, $CH_3NCHCH(CH_3)_2$), 19.3 (1C, $NHCHCH(CH_3)_2$), 19.2 (1C, $C(CH_3)_3$), 18.2 (1C, $NHCHCH(CH_3)_2$), 14.7 (1C, CH_3CHCH_2), 13.7 (1C, CH_3CHCOO), 12.0 (1C, CH_2CH_3). **IR** (ATR): $\tilde{\nu}$ = 3301 cm^{-1} (w), 2961 (m), 2932 (m), 1639 (s), 1623 (s), 1413 (m), 1097 (s), 1037 (m), 738 (m), 701 (s). **UV** (MeOH): λ_{max} (lg ε) = 205 nm (4.42), 253 (2.85),

259 (2.87), 264 (2.85). **MS** (EI, 70 eV): *m/z* (%) = 913 [M-*t*Bu]$^+$ (2), 881 (2), 481 [Me_2IleValMMMAH-C_4H_7N]$^+$ (2), 315 (4), 241 [Me_2IleVal]$^+$ (3), 199 (10), 114 [Me_2CHsBu]$^+$ (100). **MS** (ESI): *m/z* (%) = 993 [M+Na]$^+$ (77), 971 [M+H]$^+$ (100). **HRMS** (ESI): calcd. for $C_{56}H_{86}N_4O_8SiNa$ [M+Na]$^+$ 993.61071; found 993.60990.

Malevamide D (1). Silylether **14** (50 mg, 0.051 mmol, 1.0 equiv) was dissolved in THF (3 mL) and treated with TBAF·3H_2O (80 mg, 0.254 mmol, 5.0 equiv). The solution was stirred for 3 h at ambient temperature. The solvent was removed and chloroform was added to the residue. The solution was washed successively with water and brine. After evaporation of the solvent the remaining oil was purified by column chromatography [RP-18, MeOH/water (4:1)]. Malevamide D (**1**, 27 mg, 0.036 mmol, 71%) was obtained as a colorless oil. **TLC** [silica RP-18, MeOH/H_2O (4:1)]: R_f = 0.23. $[\alpha]^{28}_D$ –36.7 (*c* 0.09, MeOH). **^{1}H NMR** (600 MHz, $CDCl_3$, 2 conformers **a**,**b**, ratio 1:1): δ = 7.32-7.19 (m, 5 H, C_6H_5), 7.02 (d, 1 H, ^3J = 8.6 Hz, N*H*), 5.21 (ddd, 0.5 H, ^3J = 6.3 Hz, ^3J = 2.5 Hz, ^3J = 13.6 Hz, OCH_2C*H*-**a**), 4.88 (brs, 1 H, O*H*), 4.74 (dd, ^3J = 7.2 Hz, ^3J = 8.6 Hz, NHC*H*), 4.72-4.64 (m, 1 H, CH_3NC*H*), 4.30-4.22 (m, 1 H, OCH_2-**b**, OC*H*-**b**), 4.16-4.00 (m, 2.5 H, CH_3NCHC*H*O, CH_2CHC*H*O, CH_2NC*H*-**b**), 3.95-3.90 (m, 0.5 H, ddd, ^3J = 0.8 Hz, ^3J = 4.5 Hz, ^3J = 8.3 Hz, NC*H*CH_2-**a**), 3.83 (d, 0.5 H, ^3J = 9.1 Hz, OCH_2-**b**), 3.76-3.67 (m, 1 H, OCH_2-**a**), 3.52-3.39 (m, 2 H, NCH_2), 3.42 (s, 1.5 H, CH_2NCHCHOCH_3-**a**), 3.41 (s, 1.5 H, CH_2NCHCHOCH_3-**b**), 3.33 (s, 1.5 H, CH_3NCHCHOCH_3-**a**), 3.30 (s, 1.5 H, CH_3NCHCHOCH_3-**b**), 3.05-2.99 (m, 3 H, CH_3NCHCHO), 2.99-2.87 (m, 1.5 H, PhCH_2-**a**, PhCH_2-**b**), 2.70 (dd, 0.5 H, ^3J = 6.7 Hz, ^2J = 13.8 Hz, PhCH_2-**b**), 2.532 (d, 0.5 H, ^3J = 5.8 Hz, $(CH_3)_2$NC*H*), 2.529 (d, 0.5 H, ^3J = 5.8 Hz, $(CH_3)_2$NC*H*), 2.50-2.39 (m, 1.5 H, CH_3C*H*COO-**b**, CH_3OCHCH_2), 2.36-2.28 (m, 1.5 H, CH_3C*H*COO-**a**, CH_3OCHCH_2), 2.23-2.20 (m, 6 H, $(CH_3)_2$N), 2.11-1.92 (m, 3 H, NCHCH_2CH_2, NHCHC*H*), 1.90-1.74 (m, 4 H, CH_3CH_2C *H,* NCHCH_2CH_2, CH_3NCHC*H*$(CH_3)_2$), 1.60-1.51 (m, 1 H, CH_3CH_2), 1.28 (d, 1.5 H, ^3J = 6.9 Hz, CH_3CHCOO-**b**), 1.22-1.10 (m, 1 H, CH_3CH_2), 1.15 (d, 1.5 H, ^3J = 6.9 Hz, CH_3CHCOO-**a**), 1.03-0.98 (m, 6 H, CH_3NCHCH(CH_3)$_2$, NHCHCH(CH_3)$_2$), 0.97-0.89 (m, 9 H, NHCHCH(CH_3)$_2$, C$H_3$$CH_2CHCH_3$), 0.82 (d, 3 H, ^3J = 6.5 Hz, CH_3NCHCH(CH_3)$_2$). **^{13}C NMR** (150 MHz, $CDCl_3$,

conformer-**16a**): δ = 173.5 (1C, NHCH*C*O), 173.24 (1C, CH*C*OO), 172.0 (1C, NH*C*O), 170.5 (1C, CH_2N*C*O), 137.1 (1C, PhC_q), 129.5 (2C, *o*Ph-*C*), 128.4 (2C, *m*Ph-*C*), 126.5 (1C, *p*Ph-*C*), 81.4 (1C, CH_2NCH*C*HO), 78.1 (1C, CH_3NCH*C*HO), 76.0 (1C, OCH_2*C*H), 74.9 (1C, $(CH_3)_2$N*C*H), 63.0 (1C, O*C*H_2), 61.4 (1C, CH_2CHCHO*C*H_3), 59.83 (1C, CH_2N*C*H), 58.3 (1C, CH_3N*C*H), 57.9 (1C, CH_3NCHCHO*C*H_3), 53.8 (1C, NH*C*H), 48.12 (1C, N*C*H_2), 45.5 (1C, *C*HCOO), 43.1 (2C, N(*C*H_3)$_2$), 37.7 (1C, CH_3OCH*C*H_2), 36.6 (1C, Ph*C*H_2), 34.4 (1C, CH_3CH_2*C*H), 32.0 (1C, N*C*H_3), 30.9 (1C, NHCH*C*H), 27.0 (1C, CH_3NCH*C*H(*C*H_3)$_2$), 26.9 (1C, CH_3*C*H_2), 24.923 (1C, NCH*C*H_2), 24.3 (1C, NCH_2*C*H_2), 20.1 (1C, CH_3NCHCH(*C*H_3)$_2$), 20.0 (1C, CH_3NCHCH(*C*H_3)$_2$), 19.4 (1C, NCHCH(*C*H_3)$_2$), 18.3 (1C, NCHCH(*C*H_3)$_2$), 14.7 (1C, *C*H_3CHCOO), 14.6 (1C, CH_2CH*C*H_3), 12.0 (1C, *C*H_3CH_2). **^{13}C NMR** (150 MHz, $CDCl_3$, Me_2IleValMMMAH-(Dap-PPD)-**16b**): δ = 173.9 (1C, CH*C*OO), 170.4 (1C, CH_2N*C*O), 137.9 (1C, PhC_q), 129.3 (2C, *o*Ph-*C*), 128.3 (2C, *m*Ph-*C*), 126.4 (1C, *p*Ph-*C*), 81.2 (1C, CH_2NCH*C*HO), 69.8 (1C, OCH_2*C*H), 69.0 (1C, O*C*H_2), 61.3 (1C, CH_2CHCHO*C*H_3), 59.80 (1C, CH_2N*C*H), 48.13 (1C, N*C*H_2), 44.6 (1C, *C*HCOO), 39.7 (1C, Ph*C*H_2), 24.919 (1C, NCH_2*C*H_2), 24.2 (1C, NCH*C*H_2), 14.8 (1C, *C*H_3CHCOO). **IR** (ATR): $\tilde{\nu}$ = 3380 cm^{-1} (w), 2962 (m), 2930 (m), 1731 (m), 1621 (s), 1450 (m), 1414 (m), 1095 (s), 1039 (m), 701 (m). **UV** (MeOH): λ_{max} (lg ε) = 203 nm (4.00). **MS** (ESI): *m/z* (%) = 755 $[M+Na]^+$ (100), 733 $[M+H]^+$ (70). **MS** (EI, 70 eV): *m/z* (%) = 732 $[M]^+$ (<1), 675 [M-*s*Bu]$^+$ (1), 641 [M-Bn]$^+$ (2), 481 [Me_2IleValMMMAH-C_4H_7N]$^+$ (2), 449 (1), 396 (2), 364 (2), 304 (1), 241 [Me_2IleVal]$^+$ (3), 172 (3), 134 (4), 114 [Me_2CH*s*Bu]$^+$ (100), 91 [Bn]$^+$ (10), 70 (7). **HRMS** (ESI): calcd. for $C_{40}H_{68}N_4O_8Na$ $[M+Na]^+$ 755.49294; found 755.49257.

2-((1*S*,2*S*)-1-(Dimethylamino)-2-methylbutyl)-4-isopropyloxazol-5-yl diethyl phosphate (18). A solution of carbamate (3*R*,4*S*)-**7** (190 mg, 0.5 mmol, 0.43 equiv) in MeOH (10 mL) was treated with Pd/C (5%, 200 mg) under argon. The atmosphere was changed to hydrogen and the black suspension was stirred for 48 h at ambient temperature. The catalyst was filtered off and the celite pad was washed with MeOH followed by removement of the solvent in vacuo. The remaining oil was dissolved in dry DMF (5 mL). A solution of acid **15**

(0.300 g, 1.16 mmol, 1.0 equiv) in dry DMF (6 mL) was added. After cooling to 0 °C, dry NEt_3 (0.7 mL, 5.03 mmol, 4.3 equiv) und DEPC (0.4 mL, 2.9 mmol, 2.5 equiv) were added and the reaction mixture was stirred with warming to rt for 18 h. EtOAc (80 mL) was added and the solution was washed with saturated bicarbonate solution and brine. The organic phase was dried over $MgSO_4$ and the solvent was removed in vacuo. Chromatography (silica, EtOAc) yielded oxazole **18** as yellow oil (0.153 g, 0.41 mmol, 35%). **TLC** (silica gel, EtOAc): R_f = 0.48. $[\alpha]^{25}_D$ –39.7 (*c* 0.63, $CHCl_3$). **^{1}H NMR** (400 MHz, $CDCl_3$): δ = 4.34-4.19 (m, 4 H, $P(OCH_2CH_3)_2$), 3.25 (d, 1 H, ^3J = 10.4 Hz, NC*H*), 2.89 (dsept, 1 H, $^6J_{PH}$ = 1.0 Hz, ^3J = 6.9 Hz, $CH(CH_3)_2$), 2.21 (s, 6 H, $N(CH_3)_2$), 2.07-1.95 (m, 1 H, $CH_3CH_2CHCH_3$), 1.77-1.64 (m, 1 H, CH_2CH), 1.39 (dt, 6 H, $^4J_{PH}$ = 0.9 Hz, ^3J = 7.1 Hz, $P(OCH_2CH_3)_2$), 1.24 (d, 6 H, ^3J = 6.8 Hz, $(CH_3)_2CH$), 1.25-1.12 (m, 1 H, CH_2CH), 0.91 (t, 3 H, ^3J = 7.4 Hz, CH_3CH_2CH), 0.78 (d, 3 H, ^3J = 6.7 Hz, CH_2CHCH_3). **^{13}C NMR** (100 MHz, $CDCl_3$): d = 155.0 (1C, NCO), 143.9 (d, 1C, $^2J_{CP}$ = 10.0 Hz, OCO), 124.2 (d, 1C, $^3J_{CP}$ = 6.2 Hz, $CCH(CH_3)_2$), 67.8 (1C, NCH), 65.3 (d, 2C, $^2J_{CP}$ = 6.1 Hz, $P(OCH_2CH_3)_2$), 41.7 (2C, $N(CH_3)_2$), 34.1 (1C, CH_2CHCH_3), 25.5 (1C, CH_3CH_2CH), 24.6 (1C, $CH(CH_3)_2$), 21.3 (2C, $CH(CH_3)_2$), 16.1 (2C, $^3J_{CP}$ = 6.7 Hz, $P(OCH_2CH_3)_2$), 15.8 (1C, CH_2CHCH_3), 10.4 (1C, CH_3CH_2CH). **IR** (ATR): $\tilde{\nu}$ = 3411 cm^{-1} (w), 2967 (m), 1662 (m), 1285 (m), 1026 (s), 982 (m), 951 (m), 909 (m), 875 (m), 808 (m). **UV** ($CHCl_3$): λ_{max} (lg ε) = 294 nm (3.32), 240 (3.45). **MS** (EI, 70 eV): *m/z* (%) = 319 $[M\text{-}sBu]^+$ (100), 137 $[M\text{-}OP(OEt)_2]^+$ (53), 114 $[Me_2NCH(sBu)]^+$ (25), 85 (13), 42 (10). **MS** (ESI): 775 $[2M+Na]^+$ (100), 753 $[2M+H]^+$ (7), 399 $[M+Na]^+$ (86), 377 $[M+H]^+$ (34). **HRMS** (ESI): calcd. for $C_{17}H_{33}N_2O_5PNa$ $[M+Na]^+$ 399.20193; found 399.20175.

1-(4-(2,3-Dihydroxypropyl)phenyl)-2,2,2-trifluoroethanone oxime (20). Ketone **19** (2.063 g, 7.2 mmol, 1.0 equiv) and $H_2NOH{\cdot}HCl$ (7.460 g, 10.4 mmol, 1.5 equiv) were dissolved in pyridine (10 mL) and refluxed for 2 h. The solvent was removed in vacuo. The residue was extracted with Et_2O (100 mL), washed with saturated aqueous NH_4Cl (15 mL) and brine (15 mL) and dried over $MgSO_4$. Removing the solvent in vacuo yielded diol **20** (1.513 g, 5.75 mmol, 80%) as pale yellow solid. **TLC** (silica gel, EtOAc): R_f = 0.52. **M. p.**:

113-114 °C. **^{1}H NMR** (400 MHz, DMSO-d_6): δ = 12.66 (s, 1 H, NO*H*), 7.38 (d, 2 H, ^3J = 8.3 Hz, *o*C_6H_4-*H*), 7.34 (d, 2 H, ^3J = 8.3 Hz, *m*C_6H_4-*H*), 4.65 (d, 1 H, ^3J = 5.4 Hz, CHO*H*), 4.63 (t, 1 H, ^3J = 5.6 Hz, CH_2O*H*), 3.70-3.60 (m, 1 H, C*H*OH), 2.83 (dd, 1 H, ^2J = 13.6 Hz, ^3J = 4.3 Hz, $C_6H_4CH_2$), 2.55 (dd, 1 H, ^2J = 13.6 Hz, ^3J = 8.2 Hz, $C_6H_4CH_2$). **^{13}C NMR** (100 MHz, DMSO-d_6): δ = 144.7 (q, 1C, $^2J_{CF}$ = 30.8 Hz, *C*N), 142.2 (1C, *p*C_6H_4-*C*), 129.6 (2C, *m*C_6H_4-*C*), 128.1 (2C, *o*C_6H_4-*C*), 124.0 (1C, *ipso*C_6H_4-*C*), 121.2 (q, 1C, $^1J_{CF}$ = 274.0 Hz, F_3C), 72.2 (1C, *C*HOH), 65.4 (CH_2OH), 39.6 (1C, $C_6H_4CH_2$). **^{19}F NMR** (376 MHz, DMSO-d_6): –57.33 (s, 0.13F, CF_3), –60.39 (s, 2.87F, CF_3). **IR** (ATR): $\tilde{\nu}$ = 3231 cm^{-1} (m), 1341 (m), 1180 (s), 1110 (s), 1036 (m), 1011 (s), 962 (s), 809 (s), 741 (m), 712 (m), 686 (m), 539 (m). **UV** (MeOH): λ_{max} (lg ε) = 243 nm (3.93), 203 (4.20). **MS** (ESI): *m/z* (%) = 302 $[M+K]^+$ (27), 286 $[M+Na]^+$ (100). **HRMS** (ESI): calcd. for $C_{11}H_{12}F_3NO_3Na$ $[M+Na]^+$ 286.06615; found 286.06616.

1-(4-((2,2-Dimethyl-1,3-dioxolan-4-yl)methyl)phenyl)-2,2,2-trifluoroethanone oxime (21). Diol **20** (1.372 g 5.2 mmol, 1 equiv) was dissolved in dry DMF (8 mL). To this solution 2,2-dimethoxypropane (8 mL, 64.5 mmol, 12.4 equiv) and *p*-TsOH·H_2O (45 mg, 0.24 mmol, 0.05 equiv) were added. After stirring for 1 h at ambient temperature the reaction was stopped by addition of saturated bicarbonate solution (150 mL). The reaxtion mixture was extracted with TBME (3 × 100 mL). The extracts were combined and washed with water (20 mL) and brine (20 mL). After drying over $MgSO_4$ and removal of the solvent in vacuo acetonide **21** (1.491 g, 4.92 mmol, 95%) was obtained as colorless solid. **TLC** [silica gel, PE/EtOAc (1:1)]: R_f = 0.59. **M. p.**: 73-74 °C. **^{1}H NMR** (400 MHz, $CDCl_3$, *E*/*Z*-isomers, ratio 11:1): δ = 9.55 (brs, 0.08 H, NO*H*), 9.36 (brs, 0.92 H, NO*H*), 7.47 (d, 1.84 H, ^3J = 8.3 Hz, *o*C_6H_4-*H*), 7.42 (d, 0.16 H, ^3J = 7.8 Hz, *o*C_6H_4-*H*), 7.33 (d, 1.84 H, ^3J = 8.3 Hz, *m*C_6H_4-*H*), 7.27 (d, 0.16 H, ^3J = 7.8 Hz, *m*C_6H_4-*H*), 4.42-4.34 (m, 1 H, OC*H*), 4.04 (dd, 1 H, ^3J = 4.9 Hz, ^2J = 8.2 Hz, OCH_2), 3.68 (dd, 1 H, ^3J = 6.8 Hz, ^2J = 8.2 Hz, OCH_2), 3.03 (dd, 1 H, ^3J = 6.6 Hz, ^2J = 13.9 Hz, $C_6H_4CH_2$), 2.84 (dd, 1 H, ^3J = 6.3 Hz, ^2J = 13.9 Hz, $C_6H_4CH_2$), 1.46 (d, 3 H, $C(CH_3)_2$), 1.37 (d, 3 H, $C(CH_3)_2$). **^{13}C NMR** (100 MHz, $CDCl_3$): δ = 147.2 (q, 1C,

$^{2}J_{CF}$ = 32.2 Hz, *C*N), 140.3 (1C, *p*C_6H_4-*C*), 129.3 (2C, *m*C_6H_4-*C*), 128.8 (2C, *o*C_6H_4-*C*), 124.4 (1C, *ipso*C_6H_4-*C*), 120.7 (q, 1C, $^{1}J_{CF}$ = 274.6 Hz, F_3*C*), 109.6 (1C, *C*$(CH_3)_2$), 76.3 (1C, O*C*H), 68.9 (1C, O*C*H_2), 39.9 (1C, C_6H_4*C*H_2), 26.9 (1C, C(*C*H_3)$_2$), 25.6 (1C, C(*C*H_3)$_2$). **^{19}F NMR** (376 MHz, $CDCl_3$): δ = –62.78/–66.82 (s, 0.25/2.75F, C*F*$_3$). **IR** (ATR): $\tilde{\nu}$ = 3301 cm^{-1} (w), 1376 (m), 1337 (m), 1213 (m), 1180 (s), 1154 (m), 1118 (s), 1063 (m), 1032 (m), 1009 (s), 960 (s), 830 (s), 723 (m), 693 (m). **UV** (MeOH): λ_{max} (lg ε) = 242 nm (3.95), 204 (4.20). **MS** (ESI): *m/z* (%) = 342 $[M+K]^+$ (41), 326 $[M+Na]^+$ (100), 304 $[M+H]^+$ (33). **HRMS** (ESI): calcd. for $C_{14}H_{16}F_3NO_3Na$ $[M+Na]^+$ 326.09745; found 326.09749.

(*E*)- and (*Z*)-1-(4-((2,2-Dimethyl-1,3-dioxolan-4-yl)methyl)phenyl)-2,2,2-trifluoroethanone *O*-tosyl oxime (*E*)-(22) and (*Z*)-(22). Ketone **19** (1.423 g, 4.9 mmol, 1.0 equiv) was dissolved in EtOH (10 mL). $H_2NOH\cdot HCl$ (378 mg, 5.4 mmol, 1.1 equiv) and NaOH (217 mg, 5.4 mmol, 1.1 equiv) were added and the reaction mixture was refluxed for 4 h followed by 4 d at ambient temperature. $H_2NOH\cdot HCl$ (378 mg, 5.4 mmol, 1.1 equiv) and NaOH (217 mg, 5.4 mmol, 1.1 equiv) were added and the reaction mixture was refluxed for another 4 h. EtOH (4 mL), $H_2NOH\cdot HCl$ (1.135 g, 16.2 mmol, 3.3 equiv) and NaOH (652 mg, 16.2 mmol, 3.3 equiv) were added. The reaction mixture was stirred for 1 h under reflux and 16 h at ambient temperature. Pyridine (1 mL), $H_2NOH\cdot HCl$ (3.780 g, 54.0 mmol, 11.0 equiv) and NaOH (2.172 g, 5.4 mmol, 11.0 equiv) were added and the mixture was heated to reflux for 4 h. The reaction mixture cooled down to ambient temperature while stirring was continued for 18 h. Die suspension was filtered and the solvent was removed in vacuo. Oxime **21** (*E*/*Z*: 11:10, 1.364 g, 4.5 mmol, 91%) was obtained after chromatography (PE/EtOAc 1:1) as colorless solid. The solid was dissolved in dry DCM (12 mL) and dry NEt_3 (0.82 mL, 5.85 mmol, 1.3 equiv) was added. The reaction mixture was cooled to 0 °C. Tosyl chloride (900 mg, 4.72 mmol, 1.05 equiv) was added and the reaction mixture was stirred for 18 h with warming to ambient temperature. The suspension was diluted with DCM (100 mL) and washed successively with 2 N HCl (10 mL), water (10 mL), saturated bicarbonate solution (10 mL), and brine (10 mL). The organic phase was dried over $MgSO_4$. After the solvent was

removed in vacuo the remaining oil was subjected to column chromatography [silica, PE/EtOAc (5:1)]. First eluted was (*E*)-**22** (39 mg, 0.085 mmol, 2%), followed by an *E*/*Z*-mixture of **22** (1.578 g, 3.5 mmol, 71%) and (*Z*)-**22** (20 mg, 0.043 mmol, 1%). (*E*)-**22** was obtained as colorless solid and (*Z*)-**22** was obtained as colorless oil. (*E*)-**22: TLC** [silica gel, PE/EtOAc (5:1)]: R_f = 0.45. **M. p.**: 86-88 °C. **^{1}H NMR** (400 MHz, $CDCl_3$): δ = 7.89 (d, 2 H, ^3J = 8.4 Hz, *o*C_6H_4-*H*(Ts)), 7.38 (d, 2 H, ^3J = 8.0 Hz, *m*C_6H_4-*H*(Ts)), 7.37-7.34 (m, 4 H, *o*C_6H_4-*H*(PPD), *m*C_6H_4-*H*(PPD)), 4.38-4.31 (m, 1 H, CH_2CH), 4.04 (dd, 1 H, ^2J = 8.2 Hz, ^3J = 6.0 Hz, CH_2O), 3.65 (dd, 1 H, ^2J = 8.2 Hz, ^3J = 6.7 Hz, CH_2O), 2.99 (dd, 1 H, ^2J = 13.9 Hz, ^3J = 7.0 Hz, $C_6H_4CH_2$), 2.84 (dd, 1 H, ^2J = 13.9 Hz, ^3J = 5.8 Hz, $C_6H_4CH_2$), 2.46 (s, 3 H, $C_6H_4CH_3$), 1.43 (s, 3 H, $C(CH_3)_2$), 1.36 (s, 3 H, $C(CH_3)_2$). **^{13}C NMR** (100 MHz, $CDCl_3$): δ = 153.7 (q, 1C, $^2J_{CF}$ = 33.3 Hz, N*C*), 146.1 (1C, *p*C_6H_4-*C*(Ts)), 142.0 (1C, *p*C_6H_4-*C*(PPD)), 131.1 (1C, *ipso*C_6H_4-*C*(Ts)), 129.8 (2C, *m*C_6H_4-*C*(Ts)), 129.6 (2C, *m*C_6H_4-*C*(PPD)), 129.2 (2C, *o*C_6H_4-*C*(Ts)), 128.6 (2C, *o*C_6H_4-*C*(PPD)), 122.6 (1C, *ipso*C_6H_4-*C*(PPD)), 119.6 (q, 1C, $^1J_{CF}$ = 277.7 Hz, CF_3), 109.3 (1C, $C(CH_3)_2$) 75.9 (1C, *C*HO), 68.9 (1C, CH_2O), 40.0 (1C, $C_6H_4CH_2$), 26.9 (1C, $C(CH_3)_2$), 25.6, (1C, $C(CH_3)_2$), 21.6 (1C, $C_6H_4CH_3$). **^{19}F NMR** (376 MHz, $CDCl_3$): δ = –66.93 (s, 3F, CF_3). **IR** (ATR): $\tilde{\nu}$ = 3400 cm^{-1} (br, w), 1385 (m), 1194 (s), 1180 (s), 1145 (s), 1092 (m), 1062 (m), 1002 (m), 891 (m), 814 (m), 779 (s), 731 (m), 705 (m), 673 (s), 653 (m), 546 (s). **UV** (MeOH): λ_{max} (lg ε) = 203 nm (4.44), 229 (4.28), 256 (4.08). **MS** (ESI): *m/z* (%) = 937 $[2M+Na]^+$ (64), 480 $[M+Na]^+$ (100), 458 $[M+H]^+$ (9). **HRMS** (ESI): calcd. for $C_{21}H_{22}F_3NO_5SNa$ $[M+Na]^+$ 480.10630; found 480.10639.

(*Z*)-**22: TLC** [silica gel, PE/EtOAc (5:1)]: R_f = 0.35. **^{1}H NMR** (400 MHz, $CDCl_3$): δ = 7.90 (d, 2 H, ^3J = 8.4 Hz, *o*C_6H_4-*H*(Ts)), 7.41-7.34 (m, 4 H, *m*C_6H_4-*H*(Ts), *o*C_6H_4-*H*(PPD)), 7.29 (d, 2 H, ^3J = 8.4 Hz, *m*C_6H_4-*H*(PPD)), 4.36-4.33 (m, 1 H, OC*H*), 4.01 (dd, 1 H, ^2J = 8.2 Hz, ^3J = 6.0 Hz, OCH_2), 3.63 (dd, 1 H, ^2J = 8.2 Hz, ^3J = 6.7 Hz, OCH_2), 2.97 (dd, 1 H, ^2J = 13.9 Hz, ^3J = 7.0 Hz, $C_6H_4CH_2$), 2.33 (dd, 1 H, ^2J = 13.9 Hz, ^3J = 5.9 Hz, $C_6H_4CH_2$), 2.46 (s, 3 H, $C_6H_4CH_3$), 1.43 (s, 3 H, $C(CH_3)_2$), 1.34 (s, 3 H, $C(CH_3)_2$). **^{13}C NMR** (100 MHz, $CDCl_3$): δ = 153.8 (q, 1C, $^2J_{CF}$ = 33.3 Hz, N*C*), 145.9 (1C, *p*C_6H_4-*C*(Ts)), 142.1 (1C, *p*C_6H_4-*C*(PPD)), 131.5 (1C, *ipso*C_6H_4-*C*(Ts)), 129.9 (2C, *m*C_6H_4-*C*(Ts)), 129.6 (2C, *m*C_6H_4-*C*(PPD)), 129.1

(2C, oC$_6$H$_4$-C(Ts)), 129.0 (2C, oC$_6$H$_4$-C(PPD)), 126.0 (1C, $ipso$C$_6$H$_4$-C(PPD)), 117.4 (q, 1C, $^1J_{CF}$ = 284.0 Hz, CF$_3$), 109.4 (1C, C(CH$_3$)$_2$), 76.1 (1C, OCH), 68.9 (1C, OCH$_2$), 40.0 (1C, C$_6$H$_4$$CH_2$), 27.0 (1C, C($CH_3$)$_2$), 25.6 (1C, C($CH_3$)$_2$), 21.6 (1C, C$_6H_4$$CH_3$). **^{19}F NMR** (376 MHz, CDCl$_3$): δ = –61.88 (s, 3F, CF_3). **IR** (ATR): $\tilde{\nu}$ = 3402 cm^{-1} (br, w), 1385 (m), 1177 (s), 1156 (s), 1092 (m), 1063 (m), 1022 (m), 989 (m), 878 (m), 806 (s), 731 (m), 705 (m), 662 (m), 643 (m), 622 (m), 547 (s). **UV** (MeOH): λ_{max} (lg ε) = 202 nm (4.37), 226 (4.24), 265 (4.09). **MS** (ESI): m/z (%) = 458 [M+H]$^+$ (9), 480 [M+Na]$^+$ (100), 937 [2M+Na]$^+$ (64).

(*E*)-(22): To a solution of oxime **21** (1.371 g, 4.5 mmol, 1.0 equiv, *E*/*Z*= 11:1) in dry DCM (12 mL) dry NEt$_3$ (0.82 mL, 5.85 mmol, 1.3 equiv) was added and cooled to 0 °C. Tosyl chloride (900 mg, 4.72 mmol, 1.05 equiv) was added and the reaction mixture was stirred with warming to ambient temperature for 18 h. The suspension was diluted with DCM (100 mL) and washed with 2 N HCl (10 mL), water (10 mL), saturated bicarbonate solution (10 mL) and brine (10 mL). The organic phase was dried over MgSO$_4$ and the solvent was removed in vacuo. Chromatography on silica [PE/EtOAc (5:1)] yielded tosyloxime (*E*)-**22** as colorless oil (1.714 g, 3.75 mmol, 83%), which crystallized overnight to broad colorless plates. **^{19}F NMR** (376 MHz, CDCl$_3$): δ = –61.90/–66.93 (s, 0.17/2.83F, CF_3).

3-(4-((2,2-Dimethyl-1,3-dioxolan-4-yl)methyl)phenyl)-3-(trifluoromethyl)diaziridine (23). Ammonia (ca. 50 mL) was condensed at –40 °C. A solution of tosyloxime (*E*)-**22** (1.215 g, 2.67 mmol, 1.0 equiv) in TBME (10 mL) was added. After 2 h the cooling was switched off and the ammonia was evaporated by warming to ambient temperature for 16 h. The suspension was filtered, and the residue was washed with TBME (2·30 mL). Removal of the solvent yielded diaziridine **23** (803 mg, 2.66 mmol, quant.) as a colorless solid. **TLC** [silica gel, PE/EtOAc (4:1)]: R_f = 0.38. **M. p.**: 72-75 °C. **^{1}H NMR** (400 MHz, CDCl$_3$): δ = 7.54 (d, 2 H, ^3J = 8.4 Hz, oC$_6$H$_4$-H), 7.28 (d, 2 H, ^3J = 8.4 Hz, mC$_6$H$_4$-H), 4.314 (dddd, 0.5 H, ^3J = 6.4 Hz, ^3J = 6.4 Hz, ^3J = 6.3 Hz, ^3J = 6.3 Hz, OCH), 4.308 (dddd, 0.5 H, ^3J = 6.4 Hz, ^3J = 6.4 Hz, ^3J = 6.3 Hz, ^3J = 6.3 Hz, OCH), 4.01-3.96 (m, 1 H, OCH_2), 3.63 (dd, 0.5 H, ^3J = 6.7 Hz,

^3J = 8.2 Hz, OCH_2), 3.62 (dd, 0.5 H, ^3J = 6.8 Hz, ^3J = 8.1 Hz, OCH_2), 2.99 (dd, 1 H, ^2J = 13.9 Hz, ^3J = 6.7 Hz, $C_6H_4CH_2$), 2.81 (dd, 1 H, ^2J = 13.9 Hz, ^3J = 6.2 Hz, $C_6H_4CH_2$), 2.79 (d, 1 H, ^3J = 8.8 Hz, N*H*), 2.22 (d, 1 H, ^3J = 8.8 Hz, N*H*), 1.43 (s, 3 H, C(CH_3)$_2$), 1.34 (s, 3 H, C(CH_3)$_2$). **^{13}C NMR** (100 MHz, $CDCl_3$): δ = 140.0 (1C, *p*C_6H_4-*C*), 129.9 (1C, *ipso*C_6H_4-*C*), 129.5 (2C, *m*C_6H_4-*C*), 128.2 (2C, *o*C_6H_4-*C*), 123.5 (q, 1C, $^1J_{CF}$ = 278.3 Hz, *C*F_3), 109.3 (1C, *C*(CH_3)$_2$), 76.27 (0.5C, O*C*H), 76.23 (0.5C, O*C*H), 68.9 (1C, O*C*H_2), 57.80 (q, 0.5C, $^2J_{CF}$ = 35.9 Hz, *C*N), 57.78 (q, 0.5C, $^2J_{CF}$ = 35.9 Hz, *C*N), 39.87 (0.5C, $C_6H_4CH_2$), 39.84 (0.5C, $C_6H_4CH_2$), 26.9 (1C, C(*C*H_3)$_2$), 25.6 (1C, C(*C*H_3)$_2$). **^{19}F NMR** (376 MHz, $CDCl_3$): δ = –75.96/–75.93 (s, 1.5/1.5F, C*F*$_3$). **IR** (ATR): $\tilde{\nu}$ = 3240 cm^{-1} (m), 1376 (m), 1231 (m), 1157 (s), 1140 (s), 1070 (m), 951 (m), 867 (m), 804 (m), 710 (m), 645 (m), 570 (m). **UV** (MeOH): λ_{max} (lg ε) = 259 nm (2.40), 217 (3.98). **MS** (EI, 70 eV): *m/z* (%) = 301 [M-H]$^+$ (1), 287 [M-CH_3]$^+$ (10), 101 [$C_5H_9O_2$]$^+$ (100), 73 (16), 43 (83). **HRMS** (EI, 70 eV): calcd. for $C_{14}H_{16}F_3N_2O_2$ [M-H]$^+$ 301.11584; found 301.11573.

3-(4-((2,2-Dimethyl-1,3-dioxolan-4-yl)methyl)phenyl)-3-(trifluoromethyl)-3*H*-diazirine (24). Diaziridine **23** (707 mg, 2.34 mmol, 1.0 equiv) was dissolved in Et_2O (30 mL) and cooled to 0 °C. Dry NEt_3 (0.72 mL, 5.15 mmol, 2.2 equiv) was added, followed by dropwise addition (15 min) of iodine solution (713 mg, 2.81 mmol, 1.2 equiv) in Et_2O (25 mL). The solution was stirred for 1 h at ambient temperature, diluted with Et_2O (30 mL) and washed with water, saturated thiosulfate solution and brine. The organic phase was dried over $MgSO_4$. The solvent was removed in vacuo. The remaining yellow oil was identified as diazirine **24** (700 mg, 2.33 mmol, 99%). **TLC** [silica gel, PE/EtOAc (2:1)]: R_f = 0.64. **^{1}H NMR** (400 MHz, $CDCl_3$): δ = 7.26 (d, 2 H, ^3J = 8.3 Hz, *m*C_6H_4-*H*), 7.13 (d, 2 H, ^3J = 8.3 Hz, *o*C_6H_4-*H*), 4.34-4.26 (m, 1 H, OC*H*), 3.99 (dd, 1 H, ^2J = 8.1 Hz, ^3J = 6.0 Hz, OCH_2), 3.61 (dd, 1 H, ^2J = 8.1 Hz, ^3J = 6.8 Hz, OCH_2), 2.96 (dd, 1 H, ^2J = 13.9 Hz, ^3J = 6.8 Hz, $C_6H_4CH_2$), 2.80 (dd, 1 H, ^2J = 13.9 Hz, ^3J = 6.1 Hz, $C_6H_4CH_2$), 1.42 (s, 3 H, C(CH_3)$_2$), 1.34 (s, 3 H, C(CH_3)$_2$). **^{13}C NMR** (100 MHz, $CDCl_3$): δ = 139.6 (1C, *p*C_6H_4-*C*), 129.7 (2C, *m*C_6H_4-*C*), 127.4 (1C, *ipso*C_6H_4-*C*), 126.6 (2C, *o*C_6H_4-*C*), 122.1 (q, 1C, $^1J_{CF}$ = 273.1 Hz, *C*F_3), 109.3 (1C, *C*(CH_3)$_2$),

76.1 (1C, O*C*H), 68.8 (1C, O*C*H_2), 39.8 (1C, C_6H_4*C*H_2), 28.3 (q, 1C, $^{2}J_{CF}$ = 40.4 Hz, *C*N), 27.0 (1C, C(*C*H_3)$_2$), 25.6 (1C, C(*C*H_3)$_2$). **^{19}F NMR** (376 MHz, $CDCl_3$): δ = –65.72 (s, 3F, C*F*$_3$). **IR** (ATR): $\tilde{\nu}$ = 2988 cm^{-1} (w), 1230 (m), 1182 (m), 1150 (s), 100 (m), 938 (m). **UV** (MeOH): λ_{max} (lg ε) = 201 nm (3.91), 222 (4.09), 361 (2.43). **MS** (EI, 70 eV): *m/z* (%) = 285 $[M-CH_3]^+$ (8), 257(3), 197 (3), 177 (7), 172 (4), 151 (6), 128 (6), 101 $[C_5H_9O_2]^+$ (100), 73 (11), 43 (53). **HRMS** (ESI): calcd. for $C_{14}H_{15}F_3N_2O_2Na$ $[M+Na]^+$ 323.09778; found 323.09783.

3-(4-(3-(Trifluoromethyl)-3*H*-diazirin-3-yl)phenyl)propane-1,2-diol (25). To a solution of acetonide **24** (700 mg, 2.33 mmol, 1.0 equiv) in THF (10 mL) 2 N HCl (10 mL, 20.0 mmol, 8.6 equiv) was added. The reaction mixture was stirred for 3 h at ambient temperature. The reaction mixture was treated with saturated bicarbonate solution, until evolution of carbon dioxide had stopped. The mixture was extracted with EtOAc (3 × 50 mL). The combined extracts were washed with brine (10 mL) and dried over $MgSO_4$. Chromatography on silica gel [PE/EtOAc (1:1)] yielded diol **25** (582 mg, 2.24 mmol, 97%) as yellow solid. **TLC** [silica gel, PE/EtOAc (1:1)]: R_f = 0.18. **M. p.**: 54 °C. **^{1}H NMR** (400 MHz, $CDCl_3$): δ = 7.24 (d, 2 H, ^{3}J = 8.2 Hz, *m*C_6H_4-*H*), 7.13 (d, 2 H, ^{3}J = 8.2 Hz, *o*C_6H_4-*H*), 3.93-3.83 (m, 1 H, C*H*OH), 3.62 (dd, 1 H, ^{2}J = 11.2 Hz, ^{3}J = 2.6 Hz, C*H*$_2$OH), 3.44 (dd, 1 H, ^{2}J = 11.2 Hz, ^{3}J = 7.1 Hz, C*H*$_2$OH), 2.80-2.68 (m, 2 H, C_6H_4C*H*$_2$), 2.64 (brs, 2 H, *H*OCHCH$_2$O*H*). **^{13}C NMR** (100 MHz, $CDCl_3$): δ = 139.8 (1C, *p*C_6H_4-*C*), 129.8 (2C, *m*C_6H_4-*C*), 127.4 (1C, *ipso*C_6H_4-*C*), 126.7 (2C, *o*C_6H_4-*C*), 122.1 (q, 1C, $^{1}J_{CF}$ = 274.7 Hz, *C*F$_3$), 72.7 (1C, O*C*H), 65.9 (1C, O*C*H_2), 39.2 (1C, C_6H_4*C*H_2), 28.3 (q, 1C, $^{2}J_{CF}$ = 40.4 Hz, *C*N). **^{19}F NMR** (376 MHz, $CDCl_3$): δ = –65.72 (s, 3F, C*F*$_3$). **IR** (ATR): $\tilde{\nu}$ = 3265 cm^{-1} (m), 1347 (m), 1229 (m), 1175 (m), 1136 (s), 1086 (s), 1036 (s), 939 (m), 895 (m), 811 (m), 723 (m), 691 (m), 560 (m). **UV** (MeOH): λ_{max} (lg ε) = 201 nm (4.02), 223 (4.13), 360 (2.23). **MS** (EI, 70 eV): *m/z* (%) = 260 $[M]^+$ (0.2), 232 $[M-N_2]^+$ (23), 172 $[M-N_2-C_2H_5O_2]^+$ (100), 151 (33), 133 (14), 122 (20), 103 (17), 61 (55), 43 (22). **MS** (ESI): *m/z* (%) = 543 $[2M+Na]^+$ (21), 515 $[2M-N_2+Na]^+$ (11), 283 $[M+Na]^+$ (100), 255 $[M-N_2+Na]^+$ (8). **HRMS** (ESI): calcd. for $C_{11}H_{11}F_3N_2O_2Na$ $[M+Na]^+$ 283.06648; found 283.06643.

1-(*tert*-Butyldiphenylsilyloxy)-3-(4-(3-(trifluoromethyl)-3*H*-diazirin-3-yl)phenyl)propan-2-ol (26). Diol **25** (0.375 g, 1.44 mmol, 1.0 equiv) and imidazole (0.216 g, 3.18 mmol, 2.2 equiv) were dissolved in dry DMF (2 mL) and cooled to 0 °C. TBDPSCl (475 mg, 1.73 mmol, 1.2 equiv) was added and the reaction mixture was stirred for 1 h. The solution was diluted with PhMe/EtOAc (1:1, 50 mL) and extracted with 20% citric acid (20 mL), water (20 mL), saturated bicarbonate solution (20 mL), and brine (20 mL). The organic phase was dried over $MgSO_4$ and the solvent was removed in vacuo. The residue was purified by column chromatography [silica gel, PE/EtOAc (8:1)]. Alcohol **26** was obtained as yellow oil (572 mg, 1.15 mmol, 80%). **TLC** [silica gel, PE/EtOAc (8:1)]: R_f = 0.39. **^{1}H NMR** (400 MHz, $CDCl_3$): δ = 7.68-7.61 (m, 4 H, *m*Ph-*H*), 7.48-7.32 (m, 6 H, *o*Ph- *H, p*Ph-*H*), 7.18 (d, 2 H, ^3J = 8.3 Hz, *m*C_6H_4-*H*), 7.07 (d, 2 H, ^3J = 8.3 Hz, *o*C_6H_4-*H*), 3.95-3.84 (m, 1 H, OC*H*), 3.64 (dd, 1 H, ^2J = 10.2 Hz, ^3J = 3.8 Hz, OCH_2), 3.53 (dd, 1 H, ^2J = 10.2 Hz, ^3J = 6.6 Hz, OCH_2), 2.75 (d, 2 H, ^3J = 6.5 Hz, $C_6H_4CH_2$), 2.49 (d, 1 H, ^3J = 3.8 Hz, O*H*), 1.07 (s, 9 H, C(CH_3)$_3$). **^{13}C NMR** (100 MHz, $CDCl_3$): δ = 140.2 (1C, *p*C_6H_4-*C*), 135.5 (4C, *m*Ph-*C*), 132.9 (2C, PhC_q), 129.9 (2C, *p*Ph-*C*), 129.8 (2C, *m*C_6H_4-*C*), 127.8 (4C, *o*Ph-*C*), 127.0 (1C, *ipso*C_6H_4-*C*), 126.4 (2C, *o*C_6H_4-*C*), 122.2 (q, 1C, $^1J_{CF}$ = 274.7 Hz, *C*F_3), 72.6 (1C, O*C*H), 67.0 (1C, O*C*H_2), 39.0 (1C, $C_6H_4CH_2$), 28.3 (q, 1C, $^2J_{CF}$ = 40.3 Hz, *C*N), 26.8 (3C, C(*C*H_3)$_3$), 19.2 (1C, *C*(CH_3)$_3$). **^{19}F NMR** (376 MHz, $CDCl_3$): δ = –65.71 (s, 3F, C*F*$_3$). **IR** (ATR): $\tilde{\nu}$ = 3575 cm^{-1} (w), 1183 (m), 1154 (m), 1109 (s), 1055 (m), 938 (m), 820 (m), 739 (m), 701 (s), 612 (m), 580 (m), 550 (m). **UV** (MeOH): λ_{max} (lg ε) = 204 nm (4.47), 220 (4.46), 259 (3.14), 264 (3.13), 361 (2.39). **MS** (ESI): *m/z* (%) = 1019 $[2M+Na]^+$ (100), 521 $[M+Na]^+$ (90). **HRMS** (ESI): calcd. for $C_{27}H_{29}F_3N_2O_2SiNa$ $[M+Na]^+$ 521.18426; found 521.18432.

(*S*)-*tert*-Butyl 2-((3*R*,4*R*)-4,11,11-trimethyl-5-oxo-10,10-diphenyl-7-(4-(3-(trifluoromethyl)-3*H*-diazirin-3-yl)benzyl)-2,6,9-trioxa-10-siladodecan-3-yl)pyrrolidine-1-carboxylate (28). Boc-Dap (**27**, 50 mg, 0.174 mmol, 1.00 equiv) and alcohol **26** (84 mg, 0.168 mmol, 0.97 equiv) were dissolved in dry DCM (5 mL). DMAP (9 mg, 0.073 mmol, 0.42 equiv) and (*S*)-10-camphorsulfonic acid (10 mg, 0.042 mmol, 0.24 equiv) were added.

The solution was cooled to 0 °C and DCC (36 mg, 0.174 mmol, 1.00 equiv) was added. The reaction mixture was stirred with warming to ambient temperature for 16 h. DCU was removed by filtration and washed with DCM (20 mL). The solvent was removed in vacuo and the crude product was purified by column chromatography [silica, PE/EtOAc (8:1)]. Alcohol **26** (19 mg, 0.038 mmol, 22%) was recovered, followed by elution of ester **28** which was obtained as a yellow oil (92 mg, 0.120 mmol, 69%). **TLC** [silica gel, PE/EtOAc (8:1)]: R_f = 0.35. **^{1}H NMR** (400 MHz, $CDCl_3$, 4 isomers): δ = 7.67-7.58 (m, 4 H, *m*Ph-*H*(Si)), 7.47-7.32 (m, 6 H, *o*Ph-*H*(Si), *p*Ph-*H*(Si)), 7.26-7.15 (m, 2 H, *m*C_6H_4-*H*), 7.10-7.01 (m, 2 H, *o*C_6H_4-*H*), 5.22-5.09 (m, 1 H, OCH_2CH), 3.97-3.77 (m, 0.4 H, $CHOCH_3$), 3.77-3.59 (m, 3.2 H, $CHOCH_3$, OCH_2, NC*H*), 3.59-3.37 (m, 1.4 H, NC *H,* NCH_2), 3.37-3.25 (m, 3 H, OCH_3), 3.25-3.14 (m, 1 H, NCH_2), 3.07-2.87 (m, 2 H, $C_6H_4CH_2$), 2.50-2.34 (m, 1 H, $CHCH_3$), 1.99-1.77 (m, 2 H, $NCHCH_2CH_2$), 1.77-1.54 (m, 2 H, $NCHCH_2CH_2$), 1.51-1.41 (m, 9 H, $OC(CH_3)_3$), 1.17 (d, 3 H, ^3J = 6.7 Hz, $CHCH_3$), 1.07-1.04 (m, 9 H, $SiC(CH_3)_3$). **^{13}C NMR** (100 MHz, $CDCl_3$, main isomer): δ = 174.1 (1C, CH*C*OO), 154.3 (1C, N*C*O), 139.1 (1C, *p*C_6H_4-*C*), 135.6 (4C, *m*Ph-*C*), 132.9 (2C, PhC_q), 129.9 (2C, *m*C_6H_4-*C*), 129.8 (2C, *p*Ph-*C*), 127.7 (4C, *o*Ph-*C*), 127.4 (1C, *ipso*C_6H_4-*C*), 126.4 (2C, *o*C_6H_4-*C*), 122.1 (q, 1C, $^1J_{CF}$ = 275.6 Hz, CF_3), 83.1 (1C, $CHOCH_3$), 79.7 (1C, $OC(CH_3)_3$), 74.5 (1C, OCH_2CH), 63.9 (1C, OCH_2), 61.0 (1C, OCH_3), 59.1 (1C, NCH), 46.5 (1C, NCH_2), 42.7 (1C, $CHCH_3$), 36.3 (1C, $C_6H_4CH_2$), 28.6 (3C, $OC(CH_3)_3$), 28.3-28.1 (m, 1C, F_3CC), 26.8 (3C, $SiC(CH_3)_3$), 26.1 (1C, $NCHCH_2$), 23.8 (1C, NCH_2CH_2), 19.2 (1C, $SiC(CH_3)_3$), 13.6 (1C, $CHCH_3$). **^{19}F NMR** (376 MHz, $CDCl_3$): δ = –(65.66-65.71, m, 3F, CF_3). **IR** (ATR): $\tilde{\nu}$ = 3072 cm^{-1} (w), 1733 (m), 1691 (s), 1393 (m), 1366 (m), 1156 (s), 1096 (s), 740 (m), 702 (s), 613 (m). **UV** (MeOH): λ_{max} (lg ε) = 205 nm (4.44), 218 (4.42), 265 (3.41), 357 (2.33). **MS** (ESI): *m/z* (%) = 1558 $[2M+Na]^+$ (41), 790 $[M+Na]^+$ (100), 768 $[M+H]^+$ (18). **HRMS** (ESI): calcd. for $C_{41}H_{52}F_3N_3O_6SiNa$ $[M+Na]^+$ 790.34697; found 790.34721.

(2*R*,3*R*)-1-(*tert*-Butyldiphenylsilyloxy)-3-(4-(3-(trifluoromethyl)-3*H*-diazirin-3-yl)phenyl)propan-2-yl 3-((*S*)-1-((3*R*,4*S*)-4-((*S*)-2-((2*S*,3*S*)-2-(dimethylamino)-3-methyl-

pentanamido)-*N*,3-dimethylbutanamido)-3-methoxy-5-methylhexanoyl)pyrrolidin-2-yl)-3-methoxy-2-methylpropanoate (29). Ester **28** (48 mg, 0.062 mmol, 1.0 equiv) was treated at 0 °C with CH_2Cl_2/CF_3COOH (1:1, (2 mL) for 1.5 h. The crude product was concentrated in vacuo and dissolved in DCM (10 mL), followed by removal of the solvent in vacuo. This procedure was repeated twice. The yellow oily residue (48 mg) was treated under argon with a solution of acetate **12** (34 mg, 0.062 mmol, 1.0 equiv) in dry DCM (2 mL). When the mixture had become clear, dry NEt_3 (53 µL, 0.372 mmol, 6.0 equiv) was added. After cooling to 0 °C DEPC (20 µL, 0.133 mmol, 2.14 equiv) was added and the reaction mixture was stirred for 2.5 h. Water (3 mL) and saturated bicarbonate solution (5 mL) were added and the mixture was extracted with chloroform (3 × 50 mL). The organic phase was washed with brine (15 mL) and dried over $MgSO_4$. The solvent was removed in vacuo and the crude product was purified by column chromatography (RP-18, MeOH). Acylpyrrolidine **29** (45 mg, 0.024 mmol, 68%) was obtained as yellowish viscous oil. **TLC** (RP-18, MeOH): R_f = 0.37. **^{1}H NMR** (600 MHz, $CDCl_3$, 4 Isomers): δ = 7.69-7.57 (m, 4 H, *m*Ph-*H*), 7.47-7.32 (m, 6 H, *o*Ph- *H, p*Ph-*H*), 7.25-7.17 (m, 2 H, *m*C_6H_4-*H*), 7.06-6.98 (m, 3 H, *o*C_6H_4- *H,* N*H*), 5.23-5.09 (m, 1 H, OCH_2CHO), 4.85-4.70 (m, 1 H, NHC*H*), 4.70-4.60 (m, 1 H, CH_3NCH), 4.19-4.09 (m, 1 H, $CH_3NCHCHO$), 4.07-3.96 (m, 1 H, CH_2NCH), 3.95-3.83 (m, 1 H, $CH_2NCHCHO$), 3.75-3.55 (m, 2 H, OCH_2), 3.42-3.22 (m, 8 H, NCH_2, $CH_3NCHCHOCH_3$, $CH_2NCHCHOCH_3$), 3.08-2.90 (m, 5 H, $C_6H_4CH_2$, NCH_3), 2.54 (d, 1 H, ^3J = 5.5 Hz, $(CH_3)_2NCH$), 2.50-2.26 (m, 3 H, CH_3OCHCH_2, $CH_3OCHCHCH_3$), 2.26-2.19 (m, 6 H, $(CH_3)_2N$), 2.05-1.87 (m, 2 H, $CH_3NCHCH(CH_3)_2$, $NHCHCH(CH_3)_2$), 1.87-1.51 (m, 6 H, $NCHCH_2CH_2$, CH_3CH_2CH), 1.22-1.14 (m, 4 H, CH_3CH_2, $CH_3OCHCHCH_3$), 1.09-1.04 (m, 9 H, $C(CH_3)_3$), 1.04-0.97 (m, 6 H, $NHCHCH(CH_3)_2$, $CH_3NCHCH(CH_3)_2$), 0.96-0.89 (m, 9 H, $NHCHCH(CH_3)_2$, $CH_3CH_2CHCH_3$), 0.85-0.80 (m, 3 H, $CH_3NCHCH(CH_3)_2$). **^{13}C NMR** (150 MHz, $CDCl_3$, major diastereomer): δ = 174.0 (1C, *C*OO), 173.9 (1C, CH_2NCO), 173.4 (1C, CH_3NCO), 172.0 (1C, NH*C*O), 139.1 (1C, *p*C_6H_4-*C*), 135.5 (4C, *m*Ph-*C*), 133.1 (2C, PhC_q), 130.0 (2C, *m*C_6H_4-*C*), 129.8 (2C, *p*Ph-*C*), 127.7 (4C, *o*Ph-*C*), 127.1 (1C, *ipso*C_6H_4-*C*), 126.4 (2C, *o*C_6H_4-*C*), 122.1 (q, 1C, $^1J_{CF}$ = 274.5 Hz, CF_3), 81.5 (1C, $CH_2NCHCHO$), 78.3 (1C, $CH_3NCHCHO$), 74.9 (1C,

($(CH_3)_2$NC*H*), 74.4 (1C, OCH_2*C*HO), 63.6 (1C, O*C*H_2), 60.5 (1C, CH_2NCHCHO*C*H_3), 59.2 (1C, CH_2N*C*H), 58.4 (1C, CH_3N*C*H), 58.0 (1C, CH_3NCHCHO*C*H_3), 53.7 (1C, NH*C*H), 47.6 (1C, N*C*H_2), 43.09 (1C, CH_3OCH*C*HCH_3), 43.06 (2C, (CH_3)$_2$N), 37.7 (1C, CH_3OCH*C*H_2), 36.3 (1C, C_6H_4*C*H_2), 34.4 (1C, CH_3CH_2*C*H), 31.9 (1C, N*C*H_3), 30.6 (1C, NHCH*C*H), 28.4-28.2 (m, 1C, CF_3*C*) 27.0 (1C, CH_3NCH*C*H(CH_3)$_2$), 26.9 (1C, CH_3*C*H_2), 26.7 (3C, C(*C*H_3)$_3$), 24.8 (1C, NCH*C*H_2), 24.7 1C, NCH_2*C*H_2), 20.1 (1C, CH_3NCHCH(*C*H_3)$_2$), 20.0 (1C, CH_3NCHCH(*C*H_3)$_2$), 19.5 (1C, NHCHCH(*C*H_3)$_2$), 19.2 (1C, *C*(CH_3)$_3$), 18.2 (1C, NHCHCH(*C*H_3)$_2$), 14.6 (1C, CH_2CH*C*H_3), 13.6 (1C, CH_3OCHCH*C*H_3), 12.0 (1C, *C*H_3CH_2). **^{19}F NMR** (376 MHz, $CDCl_3$): δ = –65.65/–65.68/–65.69/–65.74 (s, 3F, C*F*$_3$). **IR** (ATR): $\tilde{\nu}$ = 3299 cm^{-1} (w), 2963 (m), 2933 (m), 1625 (m), 1158 (m), 1098 (s), 1033 (s), 981 (m), 940 (m), 704 (s). **UV** (MeOH): λ_{max} (lg ε) = 203 nm (4.50), 260 (3.30), 264 (3.30), 357 (2.25). **MS** (ESI): *m/z* (%) = 1101 [M+Na]$^+$ (40) 1079 [M+H]$^+$ (100). **HRMS** (ESI): calcd. for $C_{58}H_{86}F_3N_6O_8Si$ [M+H]$^+$ 1079.62230; found 1079.62284.

Photo malevamide D (30). Silylether **29** (44 mg, 0.04 mmol, 1.0 equiv) was dissolved in THF (2 mL). Water (3 drops) was added, followed by TBAF·3H_2O (100 mg, 0.374 mmol, 9.3 equiv). The reaction mixture was stirred at 0 °C for 2 h. The solvent was removed in vacuo and the residue was dissolved in chloroform (100 mL) and washed with water (2·10 mL). The organic phase was dried over $MgSO_4$ and the solvent was removed in vacuo. The product was purified on HPLC [Phenomenex Luna, 250 x 10, 5 µm, C-18, MeOH/water (9:1), 4.0 mL/min, 210 nm]. Diazirine **30** (11 mg, 0.013 mmol, 33%) was obtained as light yellow oil. **TLC** [silica gel RP-18, MeOH/H_2O (4:1)]: R_f = 0.23. **^{1}H NMR** (600 MHz, $CDCl_3$, four isomers, **a**, **b**, **c**, **d**): δ = 7.36-7.27 (m, 2 H, *m*C_6H_4-*H*), 7.15-7.08 (m, 2 H, *o*C_6H_4-*H*), 6.99 (d, 1 H, ^3J = 8.9 Hz, N*H*), 5.22-5.15 (m, 0.2 H, HOCH_2C*H*-**a**), 5.05-5.00 (m, 0.2 H, HOCH_2C*H*-**b**), 4.96 (brs, 0.4 H, O*H*), 4.93 (brs, 0.4 H, O*H*), 4.88 (brs, 0.2 H, O*H*), 4.76 (dd, 1 H, ^3J = 6.6 Hz, ^3J = 8.9 Hz, NHC*H*), 4.73-4.63 (m, 1 H, CH_3NC*H*), 4.61-4.56 (m, 0.3 H, HOC*H*$_2$-**d**), 4.30-4.20 (m, 0.6 H, HOC*H*$_2$C*H*-**c**), 4.12-3.88 (m, 3.3 H, CH_3NCHC*H*O, CH_2NC*H*C*H*O, HOCH_2C*H*-**d**), 3.85-3.62 (m, 1.4 H, C*H*$_2$O), 3.51-3.36 (m, 5.2 H, C*H*$_2$O-**a**, C*H*$_2$NCHCHOC*H*$_3$), 3.35-3.27 (m,

3 H, $CH_3NCHCHOCH_3$), 3.05-2.99 (m, 3 H, NCH_3), 2.99-2.70 (m, 2 H, $C_6H_4CH_2$), 2.56-2.50 (m, 1.2 H, $(CH_3)_2NC$ *H,* $CH_3OCHCHCH_3$-**b**), 2.48-2.38 (m, 1.6 H, CH_3OCHCH_2, $CH_3OCHCHCH_3$-**c**, $CH_3OCHCHCH_3$-**d**), 2.37-2.28 (m, 1.3 H, CH_3OCHCH_2, $CH_3OCHCHCH_3$-**a**), 2.26-2.20 (m, 6 H, $(CH_3)_2N$), 2.15-1.95 (m, 3 H, $NCHCH_2CH_2$, NHCHC *H,* $CH_3NCHCH(CH_3)_2$), 1.90-1.73 (m, 4 H, $NCH_2CH_2CH_2$, $(CH_3)_2NCHCH$), 1.61-1.52 (m, 1 H, CH_3CH_2), 1.31 (d, 0.9 H, $^3J = 6.9$ Hz, CH_3CHCOO-**d**), 1.28 (d, 0.9 H, $^3J = 7.2$ Hz, CH_3CHCOO-**c**), 1.17 (d, 0.6 H, $^3J = 7.0$ Hz, CH_3CHCOO-**b**), 1.10 (d, 0.6 H, $^3J = 6.8$ Hz, CH_3CHCOO-**a**), 1.05-0.80 (m, 18 H, $CH_3CH_2CHCH_3$, $NHCHCH(CH_3)_2$, $CH_3NCHCH(CH_3)_2$). **^{13}C NMR** (150 MHz, $CDCl_3$, Isomer **207a**): δ = 173.5 (1C, CH_3NCO), 173.2 (1C, CHCOO), 172.0 (1C, NHCO), 170.5 (1C, CH_2NCO), 139.1 (1C, pC_6H_4-C), 129.8 (2C, mC_6H_4-C), 127.1 (1C, *ipso*C_6H_4-C) 126.5 (2C, oC_6H_4-C), 122.2 (q, 1C, $^1J_{CF} = 274.1$ Hz, CF_3), 81.41 (1C, $CH_2NCHCHO$), 78.2 (1C, $CH_3NCHCHO$), 75.5 (1C, OCH_2CHO), 74.9 (1C, $(CH_3)_2NCH$), 62.9 (1C, CH_2OH), 61.44 (1C, $CH_2NCHCHOCH_3$), 59.9 (1C, CH_2NCH), 58.1 (1C, CH_3NCH), 58.0 (1C, $CH_3NCHCHOCH_3$), 53.8 (1C, NHCH), 48.0 (1C, NCH_2), 45.5 (1C, CHCOO), 43.1 (2C, $(CH_3)_2N$), 37.8 (1C, CH_3OCHCH_2), 36.3 (1C, $C_6H_4CH_2$), 34.5 (1C, CH_2CHCH_3), 31.9 (1C, NCH_3), 30.9 (1C, NHCHCH), 28.3 (q, 1C, $^2J_{CF} = 40.8$ Hz, CF_3C), 27.00 (1C, $CH_3NCHCH(CH_3)_2$), 26.97 (1C, CH_3CH_2), 25.0 (1C, NCH_2CH_2), 24.2 (1C, $NCHCH_2$), 20.10 (1C, $CH_3NCHCH(CH_3)_2$), 20.05 (1C, $CH_3NCHCH(CH_3)_2$), 19.4 (1C, $NCHCH(CH_3)_2$), 18.3 (1C, $NCHCH(CH_3)_2$), 14.7 (1C, CH_2CHCH_3), 14.6 (1C, CH_3CHCOO), 12.0 (1C, CH_2CH_3). **^{13}C NMR** (150 MHz, $CDCl_3$, Me_2IleValMMMAH-(Dap-Photo-PPD)-**b**): δ = 174.2 (1C, CHCOO), 139.1 (1C, pC_6H_4-C), 129.8 (2C, mC_6H_4-C), 127.1 (1C, *ipso*C_6H_4-C) 126.5 (2C, oC_6H_4-C), 122.2 (q, 1C, $^1J_{CF} = 274.1$ Hz, CF_3), 81.6 (1C, $CH_2NCHCHO$), 76.2 (1C, OCH_2CHO), 62.3 (1C, CH_2OH), 60.9 (1C, $CH_2NCHCHOCH_3$), 59.9 (1C, CH_2NCH), 48.0 (1C, NCH_2), 42.6 (1C, CHCOO), 36.2 (1C, $C_6H_4CH_2$), 28.3 (q, 1C, $^2J_{CF} = 40.8$ Hz, CF_3C), 25.0 (1C, NCH_2CH_2), 24.2 (1C, $NCHCH_2$), 12.9 (1C, CH_3CHCOO). **^{13}C NMR** (150 MHz, $CDCl_3$, Me_2IleValMMMAH-(Dap-Photo-PPD)-**c**): δ = 173.9 (1C, CHCOO), 139.9 (1C, pC_6H_4-C), 129.8 (2C, mC_6H_4-C), 127.1 (1C, *ipso*C_6H_4-C) 126.5 (2C, oC_6H_4-C), 122.2 (q, 1C, $^1J_{CF} = 274.1$ Hz, CF_3), 81.2 (1C, $CH_2NCHCHO$), 69.4 (1C, OCH_2CHO), 68.9 (1C, CH_2OH),

61.36 (1C, $CH_2NCHCHOCH_3$), 59.9 (1C, CH_2NCH), 48.2 (1C, NCH_2), 44.7 (1C, *C*HCOO), 39.1 (1C, $C_6H_4CH_2$), 28.3 (q, 1C, $^2J_{CF}$ = 40.8 Hz, CF_3C), 24.9 (1C, NCH_2CH_2), 24.2 (1C, $NCHCH_2$), 14.8 (1C, CH_3CHCOO). **^{13}C NMR** (150 MHz, $CDCl_3$, Me_2IleValMMMAH-(Dap-Photo-PPD)**-d**): δ = 173.8 (1C, CH*C*OO), 140.3 (1C, *p*C_6H_4-*C*), 129.8 (2C, *m*C_6H_4-*C*), 127.1 (1C, *ipso*C_6H_4-*C*) 126.5 (2C, *o*C_6H_4-*C*), 122.2 (q, 1C, $^1J_{CF}$ = 274.1 Hz, CF_3), 81.4 (1C, $CH_2NCHCHO$), 69.8 (1C, OCH_2CHO), 68.4 (1C, CH_2OH), 61.40 (1C, $CH_2NCHCHOCH_3$), 59.9 (1C, CH_2NCH), 48.2 (1C, NCH_2), 44.9 (1C, *C*HCOO), 39.2 (1C, $C_6H_4CH_2$), 28.3 (q, 1C, $^2J_{CF}$ = 40.8 Hz, CF_3C), 24.9 (1C, NCH_2CH_2), 24.2 (1C, $NCHCH_2$), 14.7 (1C, CH_3CHCOO). **^{19}F NMR** (376 MHz, $CDCl_3$): δ = –65.64/–65.66/–65.68/–65.70 (s, 3F, CF_3). **IR** (ATR): $\tilde{\nu}$ = 3374 cm^{-1} (w), 2960 (m), 2927 (m), 1732 (m), 1621 (s), 1452 (m), 1259 (m), 1236 (m), 1182 (m), 1155 (s), 1096 (s), 1056 (m), 938 (m), 803 (m), 544 (m). **UV** (MeOH): λ_{max} (lg ε) = 202 nm (4.38), 259 (3.18), 357 (2.40). **MS** (ESI): *m/z* (%) = 1682 $[2M+H]^+$ (10), 863 $[M+Na]^+$ (98), 841 $[M+H]^+$ (100). **HRMS** (ESI): calcd. for $C_{42}H_{67}F_3N_6O_8Na$ $[M+Na]^+$ 863.48647; found 863.48644.

2. Cytotoxicity tests

Monolayer Assay. A modified propidium iodide assay [3] was used to assess the anticancer activity of the compounds towards panels of 12 and 42 solid tumor cell lines, respectively. Adherent cells were harvested from exponential phase cultures, counted and plated in 96 well flat-bottomed microtiter plates at a cell density depending on the cell line (4,000 to 40,000 cells/well). After a 24 h recovery period to allow the cells to resume exponential growth, 10 µL of culture medium or of culture medium containing the test compound were added. Compounds were applied at 10 concentrations in duplicate and treatment continued for four days. After 4 days of continuous treatment, cells were washed with 200 µL PBS to remove dead cells and next 200 µL of a solution containing 7 µg/mL propidium iodide (PI) and 0.1%(v/v) Triton X-100 were added to the wells. After an incubation period of 1–2 hours at room temperature, fluorescence (FU) was measured using the Envision Xcitemultilabel reader (excitation λ= 530 nm, emission λ= 620 nm) to quantify the amount of attached viable cells.

Tumor cell lines

The characteristics and origin of the 42 cell lines used in the present study are shown in Table S1. Authenticity of cell lines was proven at the DSMZ by STR (short tandem repeat) analysis, a PCR based DNA-fingerprinting methodology [4,5].

Cell lines comprised 15 different tumor histotypes, each represented by one to six cell lines. They were established from cancer of the bladder (three), colon (five), head and neck (one), liver (one), lung (six), breast (three), pancreas (three), prostate (four), ovary (two), kidney (three), stomach (two) and the uterine body (one), as well as from malignant melanoma (three), sarcoma (two), and pleuramesothelioma (three). The 24 cell lines BXF 1218L, BXF 1352L, CXF 269L, GXF 251L, LIXF 575L, LXFL 1121L, LXFA 289L, LXFA 526L, LXFL 529L, LXFA 629L, MAXF 401NL, MEXF 1341L, MEXF 276L, MEXF 462NL, OVXF 899L, PAXF 1657L, PAXF 546L, PXF 1118L, PXF 1752L, PXF 698L, RXF 1781L, RXF 393NL, RXF 486L, UXF 1138L were established at Oncotest from patient-derived tumor xenografts (for reference, see Roth et al. 1999 [6]). The origin of the donor xenografts was described by Fiebig et al. 1992 and 1999 [7,8]. The other 18 cell lines were either kindly provided by the NCI (Bethesda; MD), or were purchased from ATCC (Rockville, MD), DSMZ (Braunschweig, Germany) or JCRB (Osaka, Japan).

Cell lines were routinely passaged once or twice weekly and maintained in culture for up to 20 passages. All cells were grown at 37°C in a humidified atmosphere with 5% CO_2 in RPMI1640 medium supplemented with 10% (v/v) fetal calf serum and 0.1 mg/mL gentamicin (medium and all components from PAA, Cölbe, Germany).

Growth inhibition is expressed as Test/Control × 100 (%T/C) values. Based on the T/C values, relative IC_{50} values were calculated bynon-linear regression analysis using the analysis software GraphPad Prism®, Prism 5 for windows, version 5.01 (GraphPad Software Inc., CA). The overall potency of a compound was determined by the geometric mean ('geomean') IC_{50} value of all individual IC_{50} values.

If an IC_{50} value could not be determined within the examined dose range (because a compound was either too active or lacked activity), the lowest or highest concentration studied was used for calculation of the geometric mean value.

In the heatmap presentation of IC_{50} values, the distribution of IC_{50} values obtained for a test compound in the individual cell lines is given in relation to the geometric mean IC_{50} value, obtained for all cell lines tested. The individual IC_{50} values are highlighted in colors ranging from dark green (≤1/32-fold geometric mean IC_{50}, equal to very potent compound activity or tumor sensitivity) to dark red (≥32-fold geometric mean IC_{50}, equal to lack of compound activity or tumor resistance). The heatmap presentation therefore represents an anti-proliferative "fingerprint" profile of a test compound. Furthermore, antitumor activity is displayed as a mean graph presentation of the absolute IC_{50} values.

Table S1: 42 solid tumor cell line panel as used in the present study

#	cell line: histotype		cell line: name	cell line: histopathology	origin
1	Bladder	BXF	1218L	urothel ca, pd	Xenograft
2	Bladder	BXF	1352L	urothel ca, pd	Xenograft
3	Bladder	BXF	T24	urothel ca	ATCC
4	Colon	CXF	269L	rectum carcinoma, pd	Xenograft
5	Colon	CXF	DIFI	rectum carcinoma[2)], wd	
6	Colon	CXF	HCT116	colon ca, pd	NCI
7	Colon	CXF	HT29	colon adeno ca, pd	NCI
8	Colon	CXF	RKO	epithelial colon ca, pd	ATCC
9	Gastric	GXF	251L	adeno ca, pd	Xenograft
10	Gastric	GXA	MKN45	adeno ca, pd	JCRB
11	Head&Neck	HNXF	CAL27	squamous cell ca, tongue	ATCC
12	Liver	LIXF	575L	hepatoma, wd	Xenograft
13	Lung	LXFA	289L	adeno ca, pd	Xenograft
14	Lung	LXFA	526L	adeno ca, pd	Xenograft
15	Lung	LXFA	629L	adeno ca, pd	Xenograft
16	Lung	LXFL	1121L	large cell, pd	Xenograft
17	Lung	LXFL	529L	large cell, pd	Xenograft
18	Lung	LXFL	H460	large cell, pd	NCI
19	Mammary	MAXF	401NL	adeno ca, wd	Xenograft
20	Mammary	MAXF	MCF7	mamma ca, pd	NCI
21	Mammary	MAXF	MDAMB231	mamma ca, pd	ATCC
22	Melanoma	MEXF	1341L	amelanotic melanoma	Xenograft
23	Melanoma	MEXF	276L	amelanotic melanoma	Xenograft
24	Melanoma	MEXF	462NL	amelanotic melanoma	Xenograft
25	Ovarian	OVXF	899L	papill serous adeno, wd	Xenograft
26	Ovarian	OVXF	OVCAR3	adeno ca	NCI
27	Pancreas	PAXF	1657L	adeno ca, md	Xenograft
28	Pancreas	PAXF	546L	adenosquamous, wd	Xenograft
29	Pancreas	PAXF	PANC1	epitheloid ca	ATCC
30	Prostate	PRXF	22RV1	prostate ca, pd	ATCC
31	Prostate	PRXF	DU145	prostate ca, pd	NCI
32	Prostate	PRXF	LNCAP	prostate ca, pd	DSMZ
33	Prostate	PRXF	PC3M	prostate ca, pd	NCI
34	Pleuramesoth.	PXF	1118L	pleuramesothelioma, pd	Xenograft
35	Pleuramesoth.	PXF	1752L	pleuramesothelioma	Xenograft
36	Pleuramesoth.	PXF	698L	pleuramesothelioma	Xenograft
37	Renal	RXF	1781L	hypernephroma, pd	Xenograft
38	Renal	RXF	393NL	hypernephroma, pd	Xenograft
39	Renal	RXF	486L	hypernephroma, pd	Xenograft
40	Sarcoma	SXF	SAOS2	osteosarcoma	DSMZ
41	Sarcoma	SXF	TE671	Rhabdomyosarcoma	ATCC
42	Uterus	UXF	1138L	endometrium carcino sarcoma, pd	Xenograft

ATCC : American Type Culture Collection, Rockville, MD, USA; NCI: National Cancer Institute, Bethesda, MD, USA, DSMZ : German Collection of Microorganisms and Cell Cultures, Braunschweig, Germany

[2)] established from a familial adenomatous polyposis patient with extracolonic features of the Gardner syndrome

BXF Bladder, CXF Colorectal, GXF Gastric, HNXF head&neck, LIXF Liver, LXF Lung A adeno, L large cell, MAXF Breast, MEXF Melanoma, OVXF Ovarian, PAXF Pancreatic, PXF Pleuramesothelioma, RXF Renal, UXF Uterus Body
ca = carcinoma, pap = papillary, pd = poorly differentiated, wd = well differentiated, md = moderately differentiated

Table S2: List of reference compounds used for COMPARE Analysis

#	OT-Key	Compound	Mode of Action
1	20959	AT7867	AKT and p70 S6 kinase inhibitor
2	20960	NVP-TAE684	ALK inhibitor
3	22550	Crizotinib	ALK, ROS1, c-Met ihibitor
4	10525	4-Hydroperoxy-ifosfamide, IFOACT	Alkylating agent
5	15206	Hepsulfam, NCI-17	Alkylating agent
6	18084	Treosulfan	Alkylating agent
7	10524	4-Hydroperoxy-cyclophosphamide	Alkylating agent, Crosslinking agent
8	16693	Mitomycin C	Alkylating agent, Crosslinking agent
9	18005	Thiotepa, THT	Alkylating agent, Crosslinking agent
10	12824	Elmustin, HECNU	Alkylating agent, Nitrosourea derivative
11	15985	Lomustin, CCNU	Alkylating agent, Nitrosourea derivative
12	12171	Carboplatin	Alkylating agent, Platinum compounds
13	12280	Cisplatin	Alkylating agent, Platinum compounds
14	17106	Oxaliplatin	Alkylating agent, Platinum compounds
15	17984	Tetraplatin	Alkylating agent, Platinum compounds
16	11123	Amsacrine HCl	Alkylating agent, Topoisomerase II inhibitor
17	17187	Pemetrexed, dinatrium	Antimetabolite, Antifolate
18	19215	Methotrexat Hydrat, MTX	Antimetabolite, Folic acid antagonist
19	17396	Raltitrexed, Tomudex	Antimetabolite, Folic acid antagonist
20	10619	6-Mercaptopurine	Antimetabolite, Purine antagonist
21	10623	6-Thioguanine	Antimetabolite, Purine antagonist
22	10570	5-Fluoro-2´-deoxyuridine	Antimetabolite, Pyrimidine antagonist
23	12458	Cyclocytidine	Antimetabolite, Pyrimidine antagonist
24	12460	Cyclopentenyl cytosin, NCI-14	Antimetabolite, Pyrimidine antagonist
25	12470	Cytarabin	Antimetabolite, Pyrimidine antagonist
26	14609	Ftorafur	Antimetabolite, Pyrimidine antagonist
27	14682	Gemcitabine HCl	Antimetabolite, Pyrimidine antagonist
28	10575	5-Fluorouracil	Antimetabolite, Pyrimidine antagonist, thymidilate
29	12727	DUP 785	Antimetabolite, Pyrimidine biosynthesis inhibitor
30	20969	Alisertib, free base; MLN-8237	Aurora kinase A inhibitor
31	20962	Aurora A inhibitor I	Aurora kinase A inhibitor
32	20961	ENMD-2076	Aurora kinase A inhibitor
33	20974	AT9283	Aurora kinase A, B inhibitor
34	16075	VX-680	Aurora kinase A, B inhibitor
35	12448	CYC116	Aurora kinase A, B inhibitor, VEGFR2 inhibitor
36	20964	Danusertib, PHA-739358	Aurora kinase A, B, C and Bcr-Abl kinase inhibitor
37	20963	PHA-680632	Aurora kinase A, B, C inihbitor
38	19398	GDC-0879	B-Raf inhibitor
39	18893	PLX-4720	B-Raf inhibitor
40	21919	SB-590885, GSK2118436	B-Raf inhibitor
41	20965	Vemurafenib	B-Raf inhibitor
42	17983	Tetrandrine, TRD	Ca+ channel blocker
43	23969	PHA-767491	CDC7/CDK9 inhibitor
44	11075	Alsterpaullone, APL	CDK inhibitor, CDK1/Cyclin B
45	17847	SU9516	CDK inhibitor, CDK2/Cyclin A
46	17321	Purvalanol A	CDK inhibitor, CDK2/Cyclin B
47	14559	Fascaplysin, synthetic	CDK inhibitor, CDK4/Cyclin D1
48	14574	Flavopiridol HCl	CDK2/4/7 inhibitor
49	23968	BMS 777607	c-Met inhibitor
50	23963	INCB28060	c-Met inhibitor
51	23967	MK-2461	c-Met inhibitor
52	18862	PF-04217903	c-Met inhibitor
53	22479	PHA-665752	c-Met inhibitor
54	20966	SU11274	c-Met inhibitor
55	16946	Nimesulide	COX II inhibitor
56	16697	Mitoxantron HCl	DNA Binder, Intercalator
57	11916	Bleomycin sulfate	DNA synthesis inhibitor
58	12814	Echinomycin A	DNA synthesis inhibitor
59	16692	Mithramycin A, MTM	DNA/RNA synthesis inhibitor
60	10999	Actinomycin D	DNA/RNA synthesis inhibitor, Intercalator
61	23962	NU7441	DNA-PK inhibitor

Table S2 (continued): List of reference compounds used for COMPARE Analysis

#	OT-Key	Compound	Mode of Action
62	15523	Ispinesib, mesylate	Eg5 inhibitor
63	19085	Monastrol	Eg5 inhibitor
64	16759	Monastrolin, HR22C16	Eg5 inhibitor
65	17837	S-Trityl-L-cysteine	Eg5 inhibitor
66	12886	Erlotinib HCl	EGFR inhibitor
67	14677	Gefitinib	EGFR inhibitor
68	17153	PD168393	EGFR inhibitor
69	17180	Pelitinib	EGFR inhibitor
70	21039	Afatinib, free base, BIBW2992	EGFR, HER2 inhibitor
71	15935	Lapatinib, free base	EGFR, HER2 inhibitor
72	16538	Manumycin A	Farnesyl transferase inhibitor
73	12233	Dovitinib, free base	FGFR3 inhibitor
74	10611	DON, 6-Diazo-5-oxo-L-Norleucine	Glutamate synthase inhibitor
75	18889	SB216763	Glycogen synthase kinase 3 (GSK3) inhibitor
76	20967	CHIR-99021	Glycogen synthase kinase 3β (GSK3β) inhibitor
77	23979	CUDC-101	HDAC, EGFR, HER2
78	20903	Vismodegib, free base, GDC-0449	Hedgehog inhibitor
79	20968	Dacinostat, LAQ824	Histon deacetylase (HDAC) inhibitor
80	20970	Droxinostat	Histon deacetylase (HDAC) inhibitor
81	19078	Panobinostat, free base, LBH589	Histon deacetylase (HDAC) inhibitor
82	10981	Acetyl-dinaline	Histon deacetylase (HDAC) inhibitor, Benzamide
83	16803	MS275	Histon deacetylase (HDAC) inhibitor, Benzamide
84	11219	Apicidin	Histon deacetylase (HDAC) inhibitor, Cyclic peptide
85	12598	Romidepsin	Histon deacetylase (HDAC) inhibitor, Cyclic peptide
86	16079	M344	Histon deacetylase (HDAC) inhibitor, Hydroxamate
87	17854	Suberic bis-hydroxamic acid	Histon deacetylase (HDAC) inhibitor, Hydroxamate
88	18214	Vorinostat	Histon deacetylase (HDAC) inhibitor, Hydroxamate
89	10390	Alvespimycin HCl, 17DMAG	HSP90 inhibitor
90	20971	BIIB021	Hsp90 inhibitor
91	17064	PUH64	HSP90 inhibitor
92	18186	VER-49009	HSP90 inhibitor
93	20972	AUY922	HSP90α und HSP90β inhibitor
94	20973	GSK1904529A	IGF-IR and IR inhibitor
95	11220	Apigenin	MAP kinase
96	21000	AS-703026	MEK inhibitor
97	21859	AZD8330	MEK inhibitor
98	18890	CI-1040	MEK inhibitor
99	21861	GSK11220212	MEK inhibitor
100	18891	PD0325901	MEK inhibitor
101	21860	PD318088	MEK inhibitor
102	11443	Selumetinib, AZD-6244	MEK inhibitor
103	18923	TAK-733	MEK inhibitor
104	21862	BIX02188	MEK5 inhibitor
105	21863	BIX02189	MEK5 inhibitor
106	23966	AZ 3146	Mps1 inhibitor
107	21001	AZD-8055	mTOR inhibitor
108	18892	KU0063794	mTOR inhibitor
109	21022	OSI-027	mTOR inhibitor
110	17391	Everolimus, RAD001	mTOR inhibitor
111	17397	Rapamycin	mTOR inhibitor
112	18852	Temsirolimus	mTOR inhibitor
113	12570	Dasatinib monohydrate	Multikinase inhibitor
114	18853	Nilotinib HCl	Multikinase inhibitor
115	18861	Pazopanib, free base	Multikinase inhibitor
116	21139	Regorafenib, free base	Multikinase inhibitor
117	17319	Vatalanib, free base	Multikinase inhibitor
118	17783	Sorafenib, free base	Multikinase inhibitor
119	17861	Sunitinib malate	Multikinase inhibitor
120	18864	Tandutinib, free base	Multikinase inhibitor (Flt-3, PDGFR-ß, c-kit)

Table S2 (continued): List of reference compounds used for COMPARE Analysis

#	OT-Key	Compound	Mode of Action
121	11835	BIRB796	p38 inhibitor
122	17649	SB-202190	p38 MAP kinase
123	20899	Canertinib, dihydrochloride, CI-1033	pan EGFR irreversible
124	20976	GSK1059615	pan-PI3K inhibitor
125	18914	AZD2281	PARP-inhibitor
126	17846	SU6668	PDGF/Kit/KDR inhibitor
127	19068	BKM120	PI3K inhibitor
128	21021	CAL-101	PI3K inhibitor
129	20902	GDC-0941, free base	PI3K inhibitor
130	16010	LY-294,002 HCl	PI3K inhibitor
131	23965	PI-103	PI3K inhibitor
132	20901	PIK-90, free base	PI3K inhibitor
133	23981	ZSTK474, free base	PI3K inhibitor
134	20978	AS-605240	PI3K γ inhibitor
135	11726	BEZ235, free base	PI3K/mTOR inhibitor
136	23961	NVP-BGT226	PI3K/mTOR inhibitor
137	18885	PF04691502	PI3K/mTOR inhibitor
138	21020	PKI-587	PI3K/mTOR inhibitor
139	17832	Staurosporine	PKC modulator, PKC inhibitor
140	17638	Satraplatin	Platinum IV agent
141	11738	BI 2536	Plk1 inhibitor
142	18913	BI 6727	Plk1 inhibitor
143	17066	GSK461364A	Plk1 inhibitor
144	23964	ON-01910	Plk1 inhibitor
145	16662	Methyl-GAG	Polyamine synthesis inhibitor
146	12878	Epoxomicin, synthetic	Proteasome inhibitor
147	16665	MG132	Proteasome inhibitor
148	12078	Bortezomib	Proteasome inhibitor (26S)
149	11137	Anguidine	Protein synthesis inhibitor
150	15301	Homoharringtonine	Protein synthesis inhibitor
151	15380	Hydroxyurea	Ribonucleotid reductase (RNR) inhibitor
152	17451	Rifamycin SV	RNA polymerase (bact.) inhibitor
153	20977	BIBR1532	Telomerase inhibitor
154	17974	MST-312	Telomerase inhibitor
155	10837	7-Ethyl-10-hydroxy-camptothecin, SN38	Topoisomerase I inhibitor
156	12160	Camptothecin	Topoisomerase I inhibitor
157	18065	Topotecan HCl	Topoisomerase I inhibitor
158	12579	Daunorubicin HCl	Topoisomerase II inhibitor
159	12707	Doxorubicin HCl	Topoisomerase II inhibitor
160	12911	Etoposide, VP16	Topoisomerase II inhibitor
161	15419	Idarubicin	Topoisomerase II inhibitor
162	17324	Pyrazoloacridine, NCI-15	Topoisomerase II inhibitor
163	17976	Teniposide, VM26	Topoisomerase II inhibitor
164	12700	Docetaxel, Taxtotere, TXT	Tubulin binder
165	12877	Epothilon D	Tubulin binder
166	20979	Epothilone A	Tubulin binder
167	12876	Epothilone B, free base	Tubulin binder
168	17122	Paclitaxel	Tubulin binder
169	18202	Vinorelbine bistartrate, Navelbine	Tubulin binder
170	18194	Vinblastine sulfate, VELBE	Tubulin binder, Vincaalkaloid
171	18197	Vincristin sulfate	Tubulin binder, Vincaalkaloid
172	18199	Vindesin sulfate	Tubulin binder, Vincaalkaloid
173	18200	Vinflunine, di-tartrate	Tubulin binder, Vincaalkaloid
174	15444	Imatinib, mesylate	Tyrosine kinase inhibitor (Bcr-Abl)
175	20975	OSI-930	Tyrosine kinase inhibitor (c-kit, VEGFR-2)
176	20980	Plinabulin, NPI-2358	vascular disrupting agent
177	11747	BI-20, Intedanib	VEGFR, PDGFR, FGFR inhibitor

Table S3. Cytotoxicity of malevamide D and photomalevamide D against a panel of 42 human cancer cell lines.

Heatmap of Absolute IC50 and IC70 [µM]

	IC70 Li-0111 malevamide D	IC50 Li-0111 malevamide D	IC70 Li-0112 photomalevamide D	IC50 Li-0112 photomalevamide D
MAXF 401	0,0002	0,0002	0,049	0,043
CXF HT-29	0,0003	0,0002	0,09	0,081
LXFL 529	0,0004	0,0003	0,113	0,081
PXF 1752	0,0004	0,0003	0,095	0,069
RXF 393	0,0004	0,0002	0,117	0,064
CXF RKO	0,0005	0,0004	0,107	0,098
MAXF MCF-7	0,0005	0,0002	0,13	0,069
PRXF DU-145	0,0005	0,0005	0,133	0,107
UXF 1138	0,0005	0,0004	0,078	0,056
HNXF CAL-27	0,0006	0,0004	0,099	0,073
LIXF 575	0,0006	0,0003	0,126	0,09
MEXF 462	0,0006	0,0004	0,099	0,081
OVXF NIH:OVCAR-3	0,0006	0,0005	0,092	0,068
LXFA 629	0,0007	0,0004	0,165	0,105
LXFL NCI-H460	0,0007	0,0006	0,239	0,196
PRXF PC-3M	0,0007	0,0005	0,16	0,113
RXF 486	0,0007	0,0005	0,619	0,39
CXF DiFi	0,0008	0,0006	0,347	0,182
CXF HCT-116	0,0008	0,0007	0,215	0,157
LXFL 1121	0,0008	0,0006	0,115	0,091
MAXF MDA-MB-231	0,0008	0,0005	0,163	0,093
MEXF 1341	0,0008	0,0005	0,159	0,085
PRXF 22Rv1	0,0008	0,0006	0,113	0,077
BXF T-24	0,0009	0,0008	0,39	0,298
LXFA 526	0,0009	0,0007	0,166	0,115
PAXF PANC-1	0,0009	0,0006	0,176	0,108
PRXF LNCaP	0,0009	0,0007	0,168	0,13
BXF 1218	0,001	0,0006	0,111	0,085
BXF 1352	0,001	0,0009	0,2	0,148
GXA MKN45	0,001	0,0005	0,273	0,128
OVXF 899	0,001	0,0007	0,835	0,476
PXF 698	0,001	0,0006	0,111	0,061
SXFO Saos-2	0,001	0,0008	0,217	0,128
PAXF 1657	0,004	0,001	10	0,389
GXF 251	0,005	0,0006	2,83	0,344
SXFS TE671	0,005	0,002	1,623	0,5
RXF 1781	0,05	0,0004	0,198	0,134
CXF 269	0,051	0,001	10	0,19
MEXF 276	0,053	0,0008	5,209	0,132
LXFA 289	0,1	0,051	10	5,261
PAXF 546	0,1	0,05	10	5,05
PXF 1118	0,1	0,006	10	1,48
**geomean IC_{70} [µM]**	**0,00149**	**0,00068**	**0,316**	**0,154**

1/32 1/16 1/8 1/4 1/2 1 2 4 8 16 32 -fold mean IC_{50}

sensitive cell lines — resistant cell lines

Table S4. Results of the Compare analyses performed for malevamide D and photomalevamide D.

Compare Analysis: malevamide D (1, Li-0111)

(based on in vitro antitumor activity in FA)

30.09.2013

Absolute IC50 Ranking

#	Compound	Mode of Action	Spearman coefficient	p-value	N
1	Li-0111		1,000	p<0.001	42
2	Photo-Malevamid D		0,742	0,000	42
3	GSK461364A	Plk1 inhibitor	0,541	0,000	42
4	Vinblastine sulfate	Tubulin binder	0,507	0,000	42
5	ON-01910	Plk1 inhibitor	0,495	0,001	42
6	Epothilone A	Tubulin binder	0,479	0,001	42
7	TAE684	ALK inhibitor	0,452	0,002	41
8	VX-680	Aurora kinase A, B	0,449	0,002	42
9	Dasatinib monohydrate	Multikinase inhibitor (Bcr-	0,445	0,002	42
10	PUH64	Hsp90 inhibitor	0,445	0,003	40
11	BI 6727 3HCl	Plk1 inhibitor	0,437	0,003	42
12	Alvespimycin HCl	Hsp90 inhibitor	0,430	0,004	42
13	Vatalanib, free base	Multikinase inhibitor	0,418	0,007	38
14	BI 2536	Plk1 inhibitor	0,403	0,007	42
15	Epothilon D	Tubulin binder	0,401	0,007	42
16	Nilotinib HCl	Multikinase inhibitor (Bcr-	0,396	0,008	42
17	Docetaxel	Tubulin binder	0,386	0,010	42
18	SU11274	c-Met inhibitor	0,379	0,011	42
19	Plinabulin	Vascular disrupting	0,378	0,011	42
20	AT9283	Aurora kinase A, B	0,372	0,013	42

Compare Analysis: Diazirinyl-subst malevamide D (30, Li-0112)

(based on in vitro antitumor activity in FA)

17.10.2013

Absolute IC50 Ranking

#	OT-Key	Compound	Mode of Action	Spearman coefficient	p-value	N
1	25000	Photo-Malevamid D		1,000	p<0.001	42
2	18194	Vinblastine sulfate	Tubulin binder	0,828	0,000	42
3	23244	Li-0111		0,742	0,000	42
4	18200	Vinflunine, di-tartrate	Tubulin binder	0,683	0,000	42
5	18197	Vincristin sulfate	Tubulin binder	0,681	0,000	42
6	18199	Vindesin sulfate hydrate	Tubulin binder	0,617	0,000	42
7	12700	Docetaxel	Tubulin binder	0,590	0,000	42
8	11738	BI 2536	Plk1 inhibitor	0,508	0,000	42
9	17066	GSK461364A	Plk1 inhibitor	0,499	0,001	42
10	18913	BI 6727 3HCl	Plk1 inhibitor	0,490	0,001	42
11	23964	ON-01910	Plk1 inhibitor	0,486	0,001	42
12	17122	Paclitaxel	Tubulin binder	0,480	0,001	42
13	17064	PUH64	Hsp90 inhibitor	0,468	0,002	40
14	15985	Lomustine	Alkylating agent,	0,448	0,002	42
15	17976	Teniposide	Topoisomerase II	0,419	0,006	40
16	12579	Daunorubicin HCl	Topoisomerase II	0,417	0,005	42
17	16697	Mitoxantron 2HCl	DNA binder	0,414	0,005	42
18	12911	Etoposide	Topoisomerase II	0,399	0,007	42
19	12824	Elmustin	Alkylating agent,	0,392	0,008	42
20	16075	VX-680	Aurora kinase A, B	0,373	0,013	42

References:

1. Miyazaki, K.; Kobayashi, M.; Natsume, T.; Gondo, M.; Mikami, T.; Sakakibara, K.; Tsukagoshi, S. *Chem. Pharm. Bull.* **1995**, *43*, 1706-1718.
2. Sone, H.; Shibata, T.; Fujita, T.; Ojika, M.; Yamada, K. *J. Am. Chem. Soc.* **1996**, *118*, 1874-1880.
3. Dengler, W. A.; Schulte, J.; Berger, D. P.; Mertelsmann, R.; Fiebig, H.-H. *Anti-Cancer Drugs* **1995**, *6*, 522–532.
4. Masters, J. R.; Thomson, J. A.; Daly-Burns, B.; Reid, Y. A.; Dirks, W. G.; Packer, P.; Toji, L. H.; Ohno, T.; Tanabe, H.; Arlett, C. F.; Kelland, L. R.; Harrison, M.; Virmani, A.; Ward, T. H.; Ayres, K. L.; Debenham P. G. *Proc. Natl. Acad. Sci. USA* **2001**, *98*, 8012-8017.
5. Dirks, W. G.; Faehnrich, S.; Estella, I. A.; Drexler, H. G. *ALTEX* **2005**, *22*, 103-109.
6. Roth, T.; Burger, A. M.; Dengler, W.; Willmann, H.; Fiebig, H. H. Human tumor cell lines demonstrating the characteristics of patient tumors as useful models for anticancer drug screening. In *Relevance of Tumor Models for Anticancer Drug Development*; Fiebig, H. H.; Burger, A. M., Eds.; Contributions to Oncology, Vol. 54; Karger: Basel, 1999; pp 145–156.
7. Fiebig, H. H.; Dengler, W. A.; Roth T. Human tumor xenografts: Predictivity, characterization, and discovery of new anticancer agents. In *Relevance of Tumor Models for Anticancer Drug Development*; Fiebig, H. H.; Burger, A. M., Eds.; Contributions to Oncology, Vol. 54; Karger: Basel, 1999; pp 29–50.
8. Fiebig, H. H.; Berger, D. P.; Dengler, W. A.; Wallbrecher, E.; Winterhalter, B. R. Combined *in vitro*/*in vivo* test procedure with human tumor xenografts. In *Immunodeficient Mice in Oncology*; Fiebig, H. H.; Berger, D. P., Eds.; Contributions to Oncology, Vol. 42; Karger: Basel, 1992; pp 321–351.

(*S*)-Benzyl methyl(3-methyl-1-oxobutan-2-yl)carbamate (5)

^{1}H NMR ($CDCl_3$, 400 MHz)

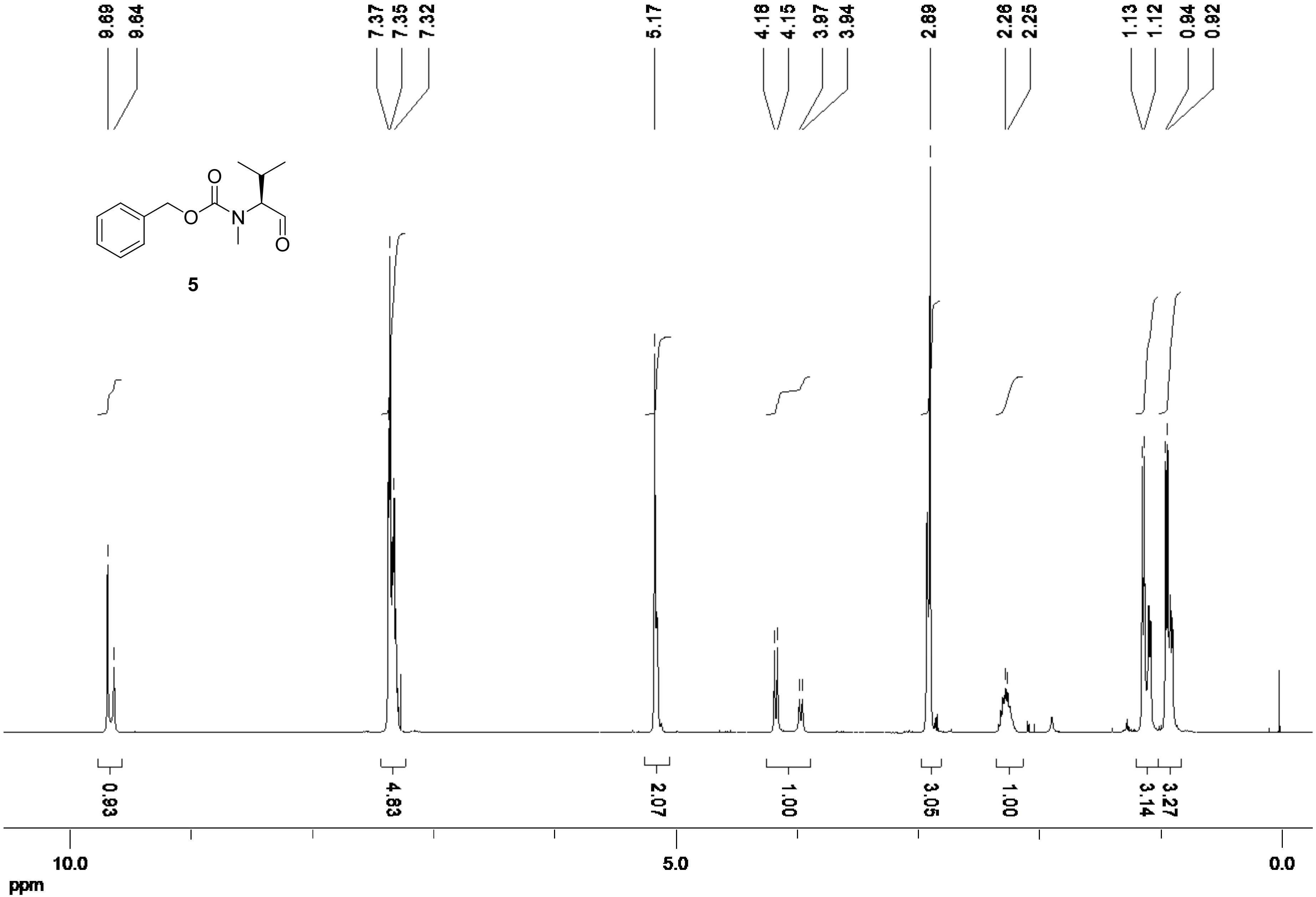

(*S*)-Benzyl methyl(3-methyl-1-oxobutan-2-yl)carbamate (5)

^{13}C NMR ($CDCl_3$, 100 MHz)

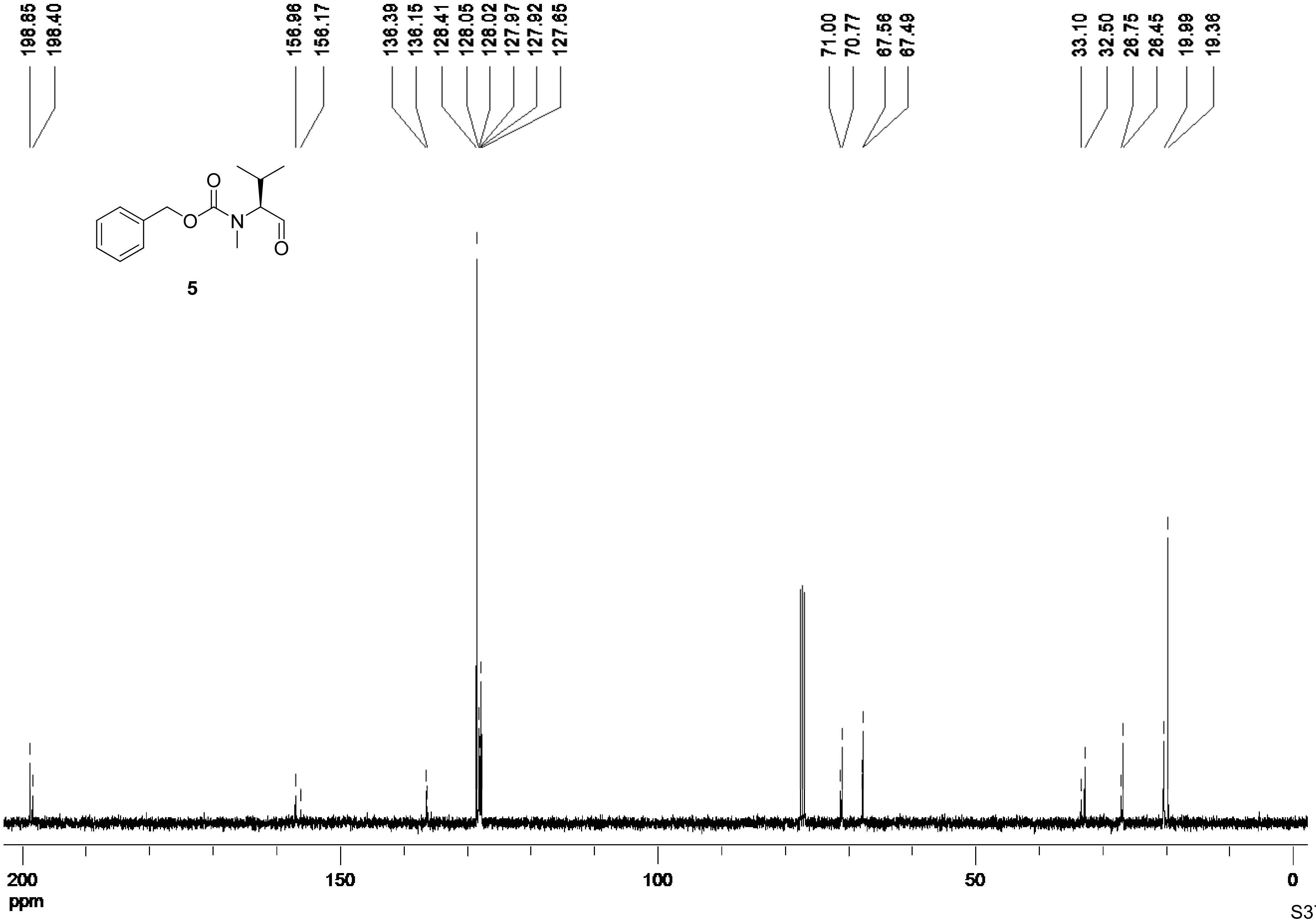

(3*S*,4*S*)-*Tert*-butyl4-((benzyloxycarbonyl)(methyl)amino)-
3-hydroxy-5-methylhexanoate (6), ^{1}H NMR ($CDCl_3$, 400 MHz)

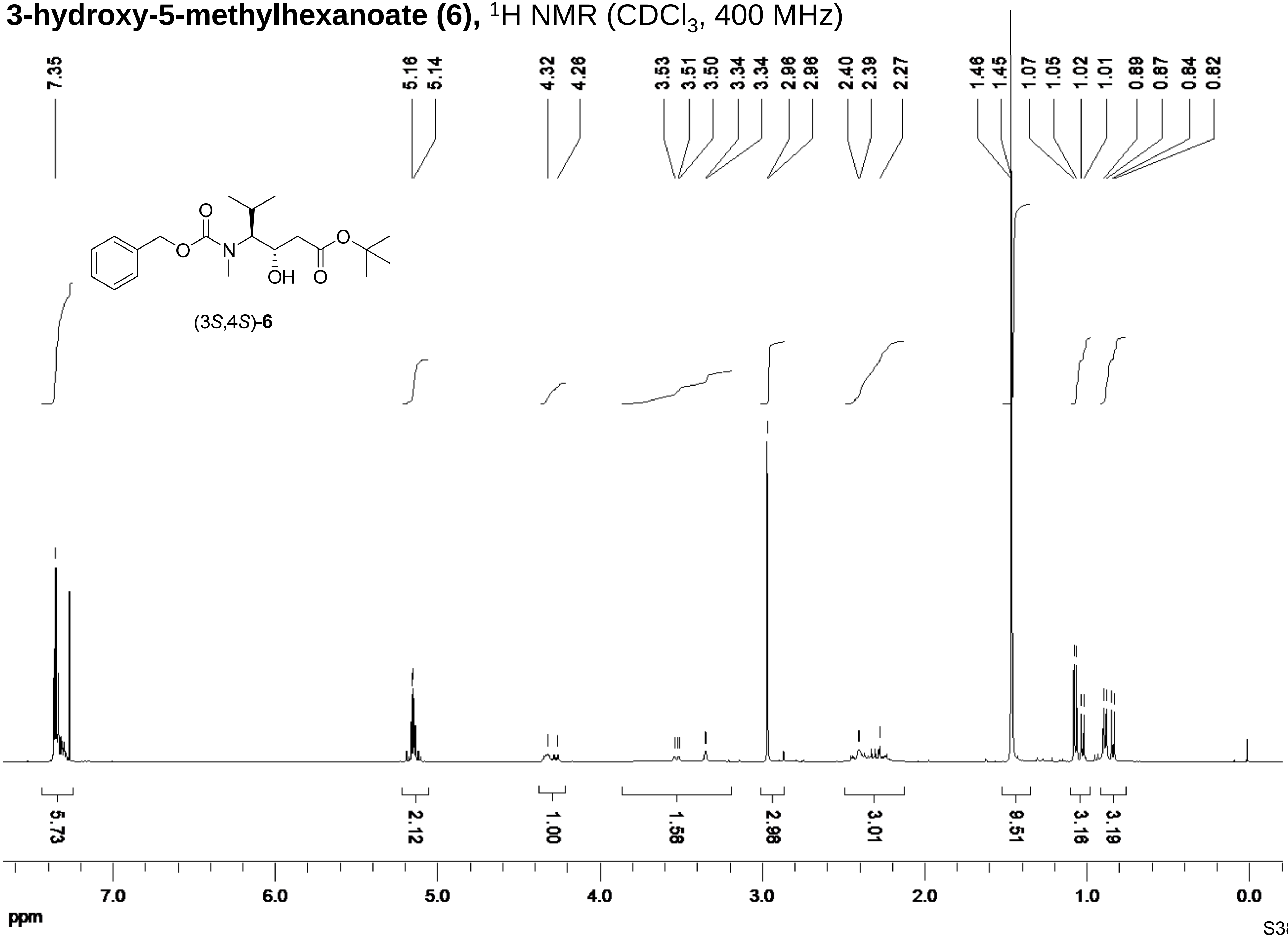

(3*S*,4*S*)- *Tert*-butyl 4-((benzyloxycarbonyl)(methyl)amino)-3-hydroxy-5-methylhexanoate (6), ^{13}C NMR ($CDCl_3$, 100 MHz)

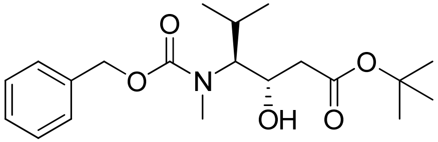

(3*S*,4*S*)-**6**

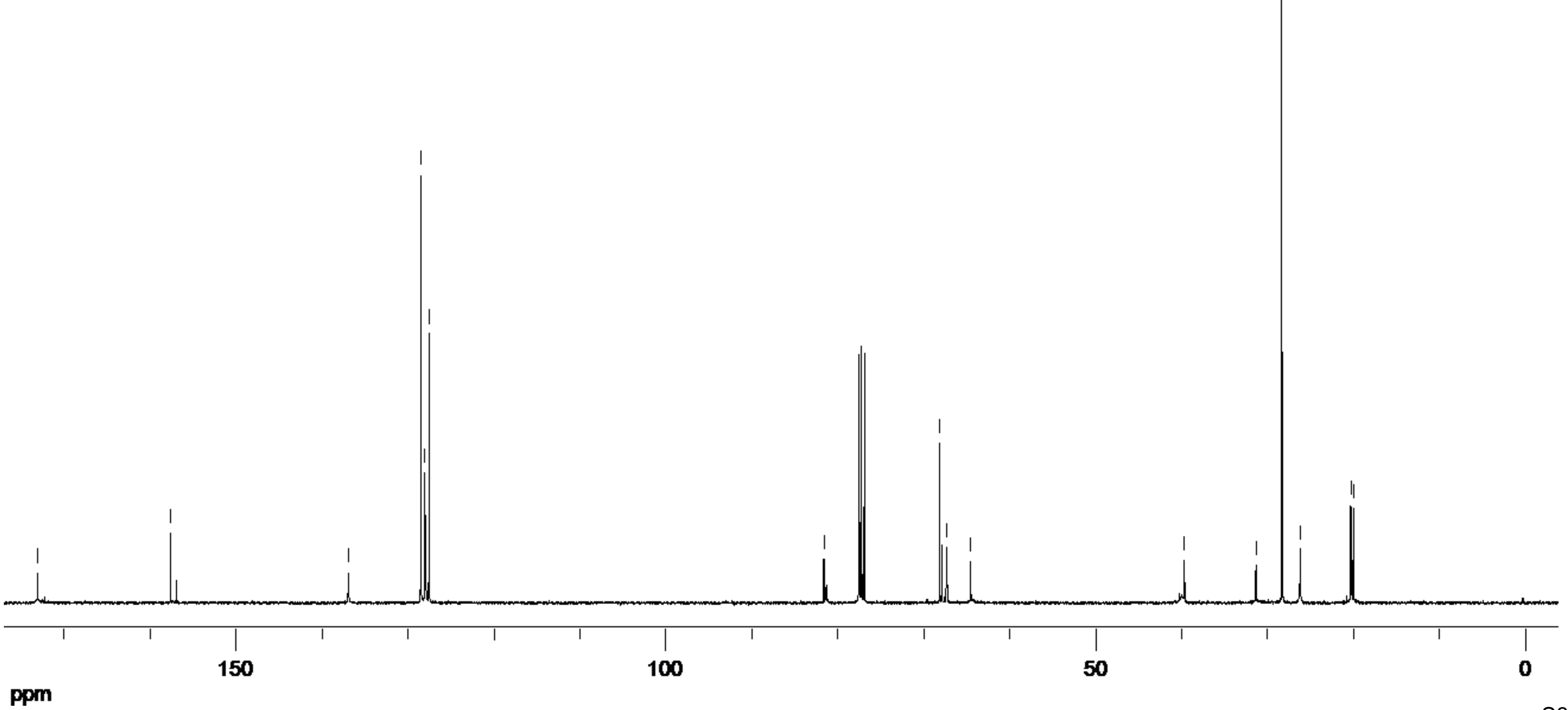

(3*R*,4*S*)-*Tert*-butyl 4-((benzyloxycarbonyl)(methyl)amino)-3-hydroxy-5-methylhexanoate (6), ^{1}H NMR ($CDCl_3$, 400 MHz)

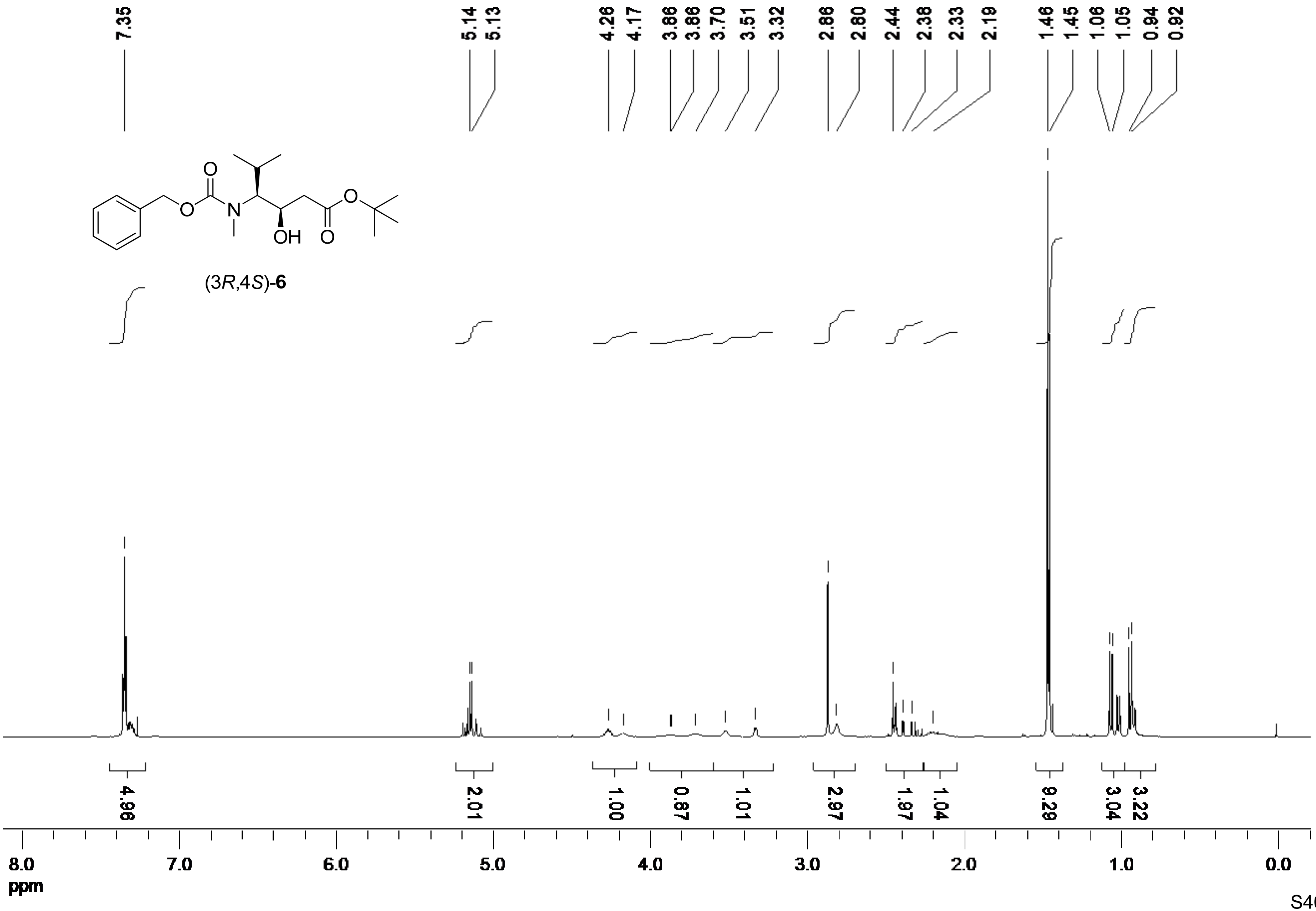

(3*R*,4*S*)-*Tert*-butyl 4-((benzyloxycarbonyl)(methyl)amino)-3-hydroxy-5-methylhexanoate (6), ^{13}C NMR ($CDCl_3$, 100 MHz)

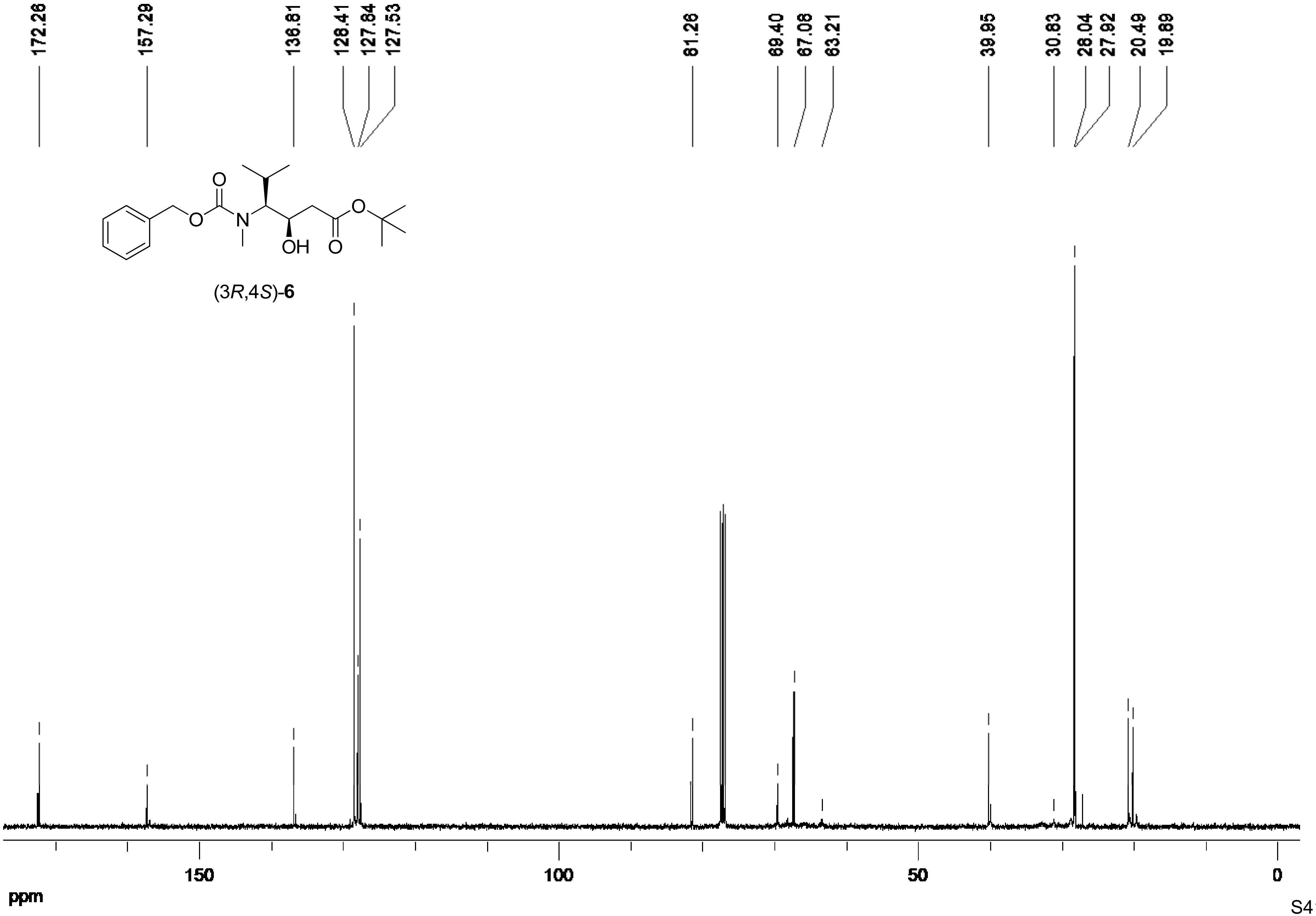

Me$_2$Ile-Val-MMMAH-*O*-*t*Bu (11)

^{13}C NMR ($CDCl_3$, 100 MHz)

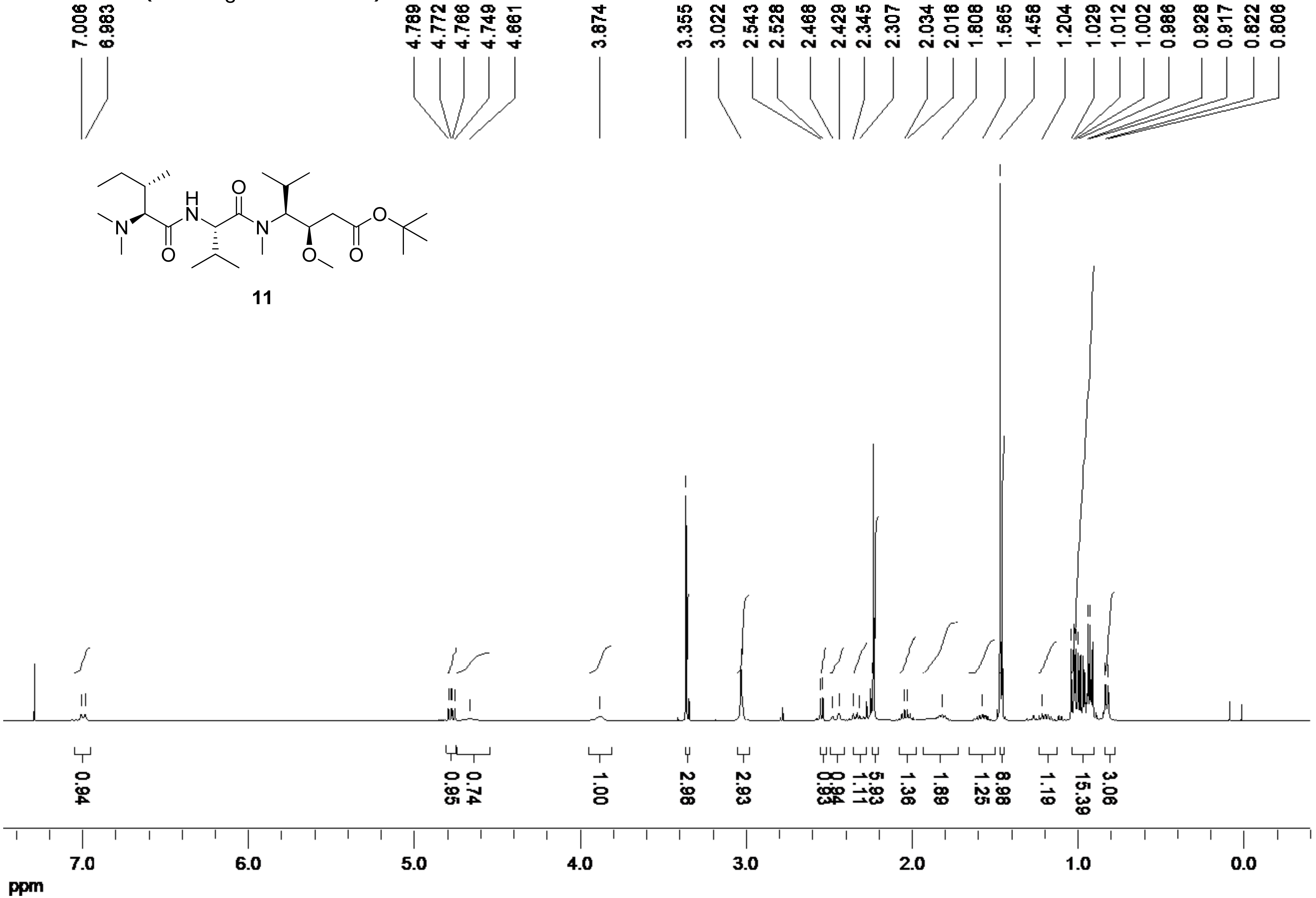

Me$_2$Ile-Val-MMMAH-*O*-*t*Bu (11)

^{13}C NMR (CDCl$_3$, 100 MHz)

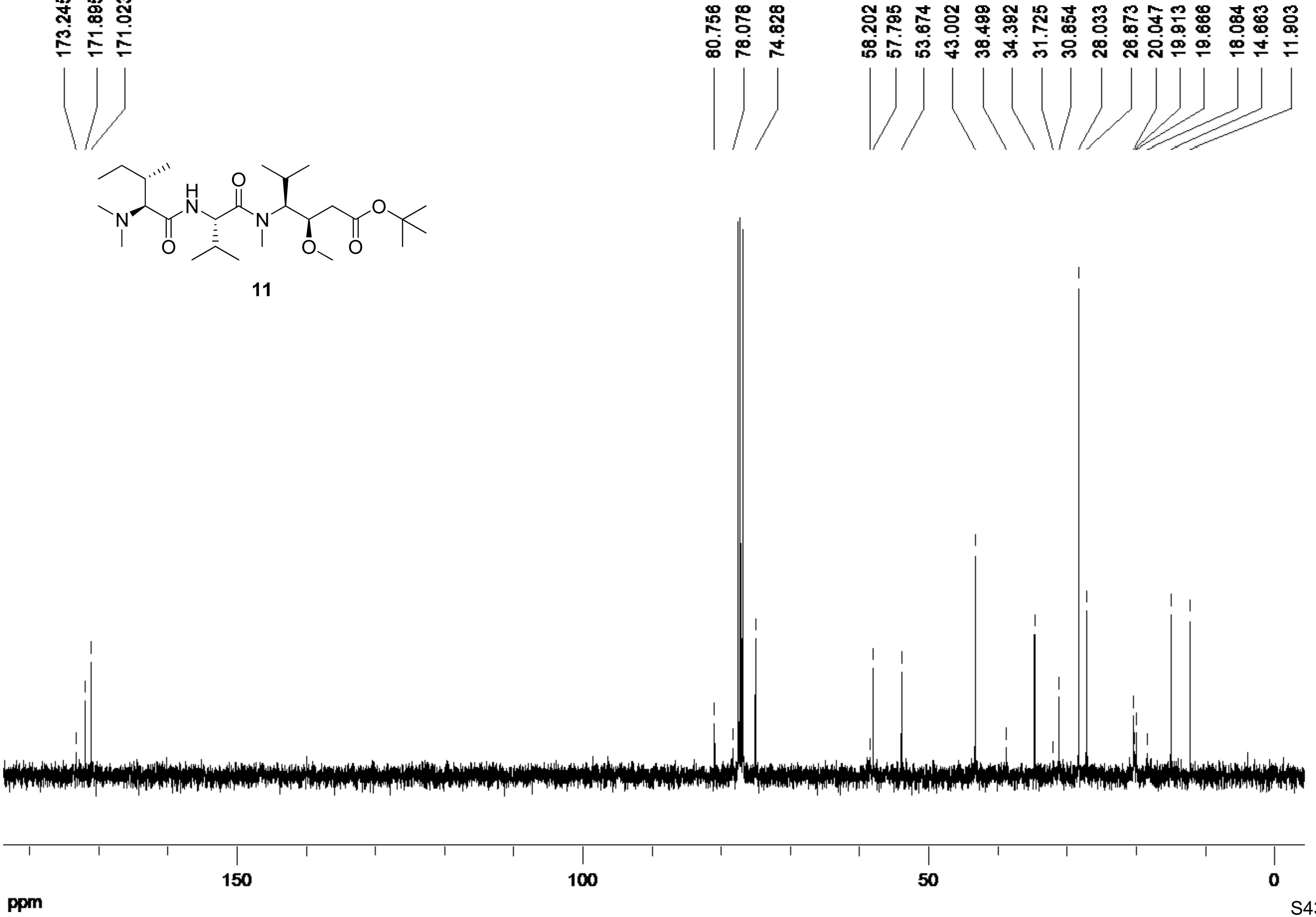

11

Me$_2$Ile-Val-MMMAH-OH TFA salt (12)

^{1}H NMR ($CDCl_3$, 600 MHz)

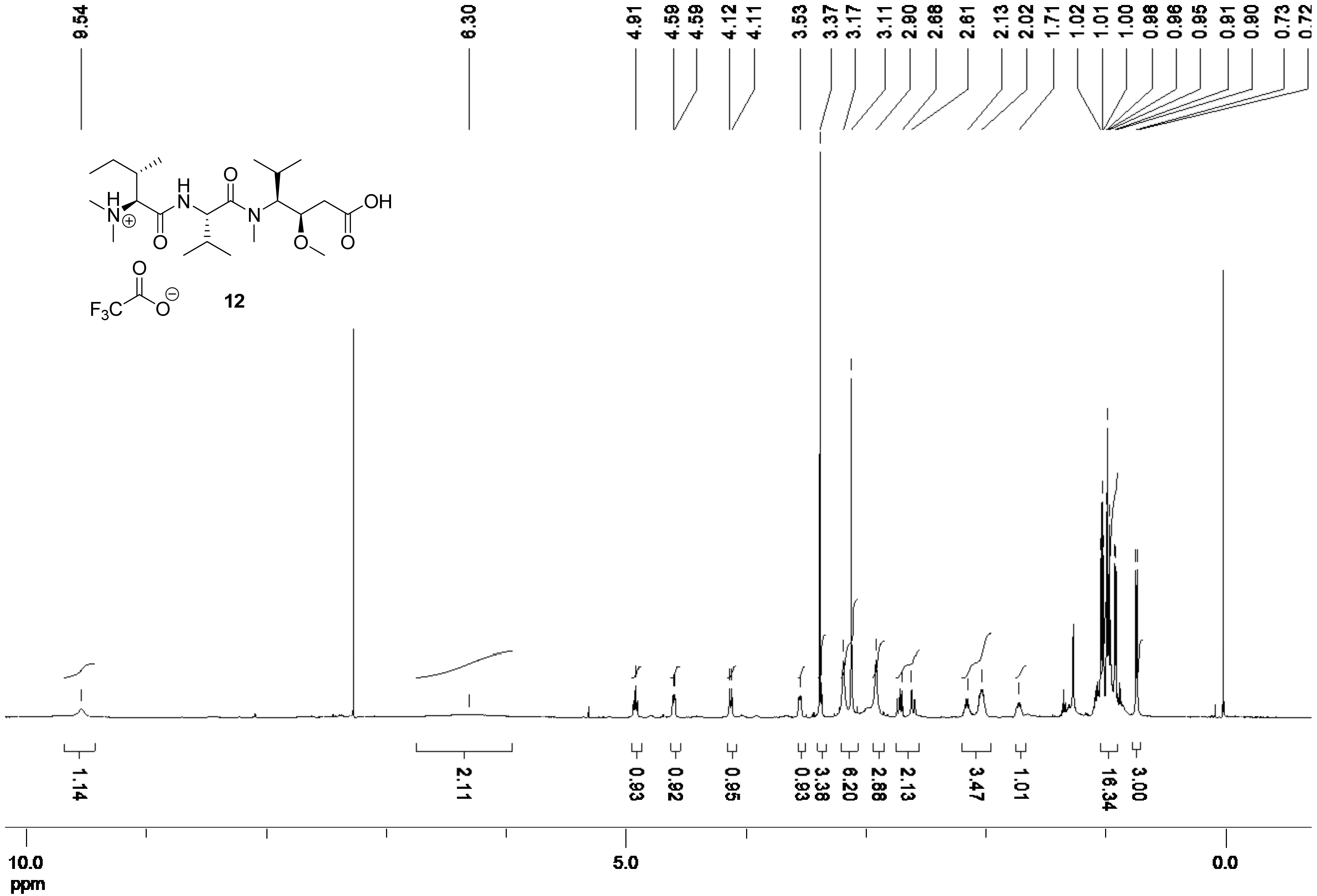

Me_2Ile-Val-MMMAH-OH TFA salt (12)

^{13}C NMR ($CDCl_3$, 150 MHz)

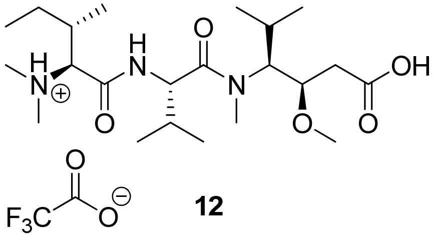

12

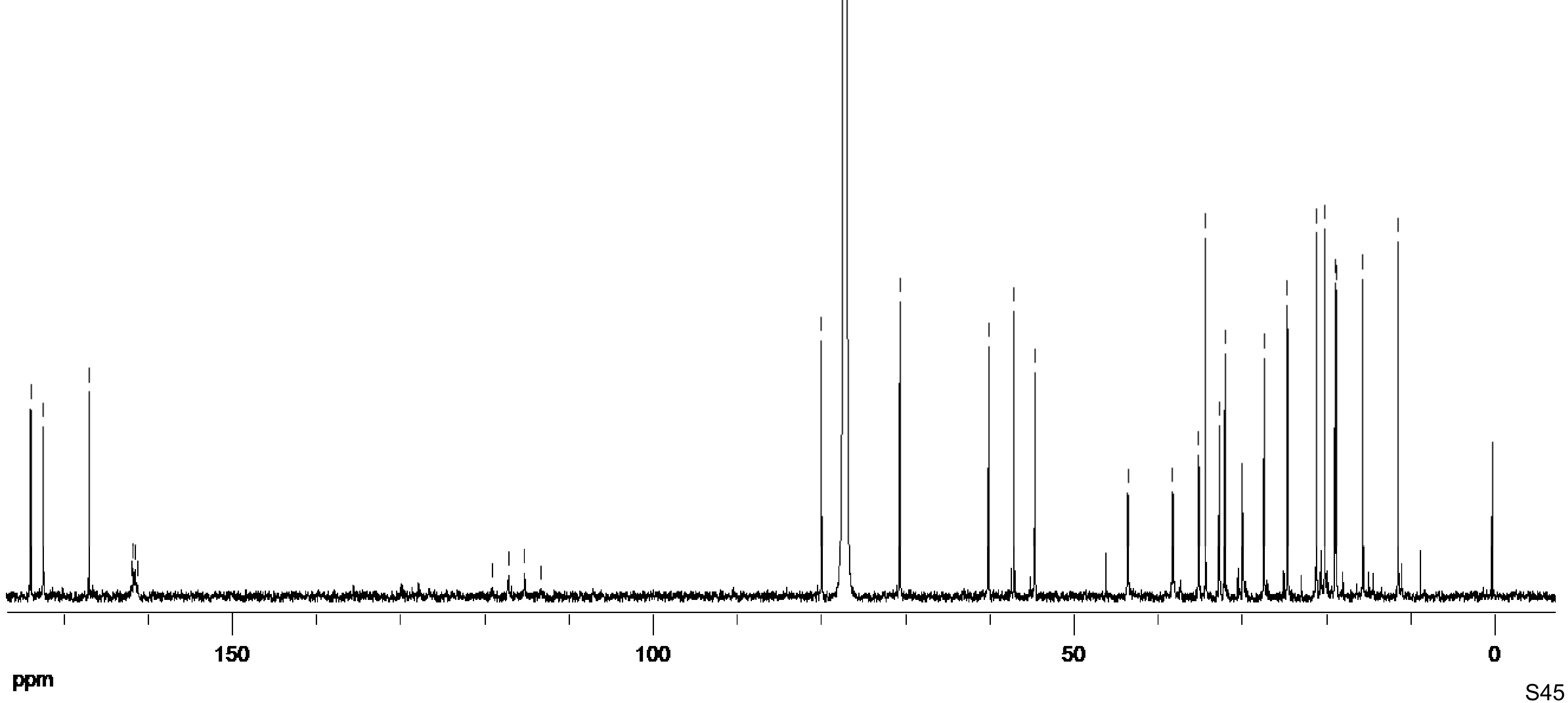

Dap-PPD-O[1]TBDPS TFA salt (13)

^{1}H NMR ($CDCl_3$, 600 MHz)

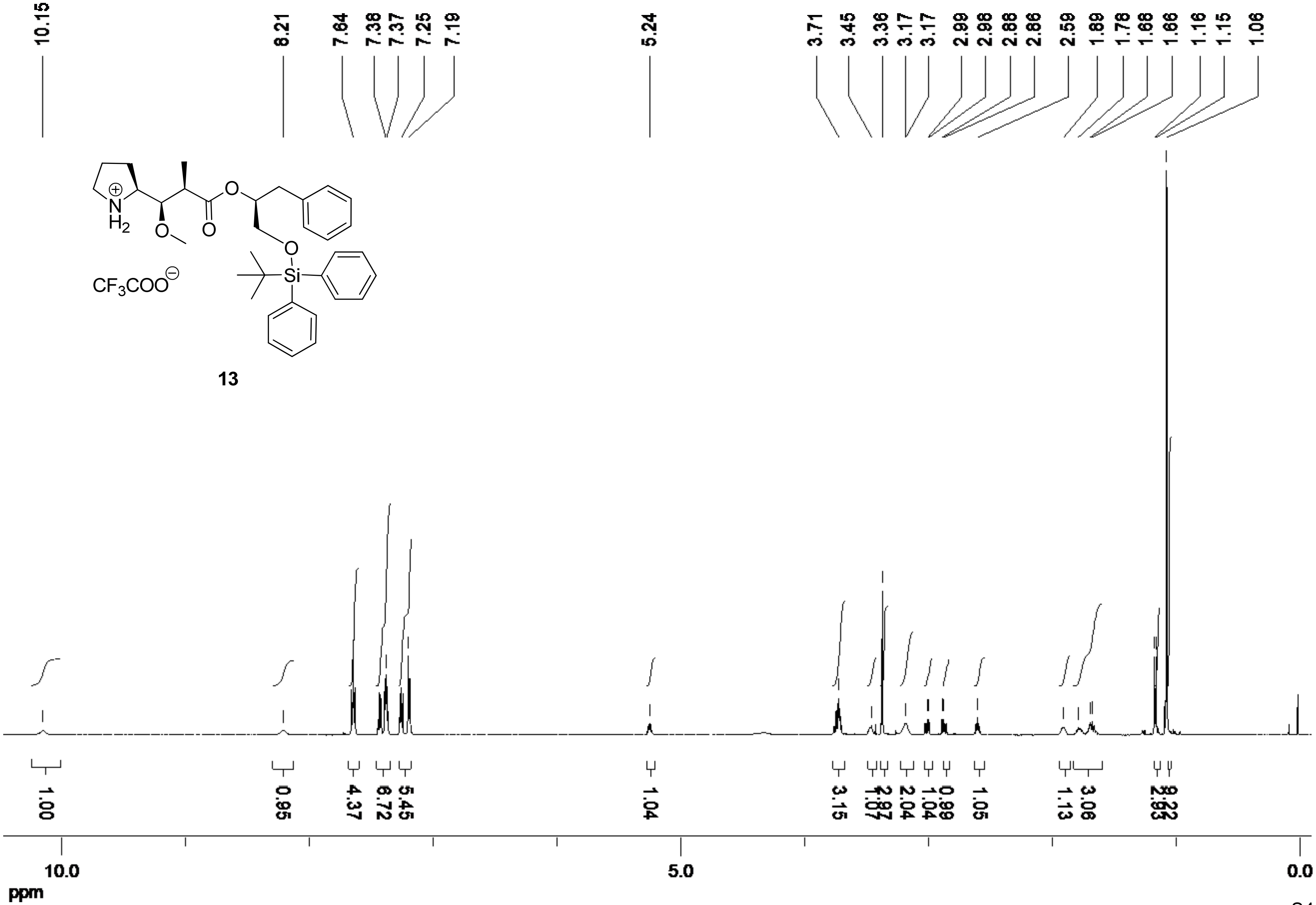

Dap-PPD-O[1]TBDPS TFA salt (13)

^{13}C NMR ($CDCl_3$, 150 MHz)

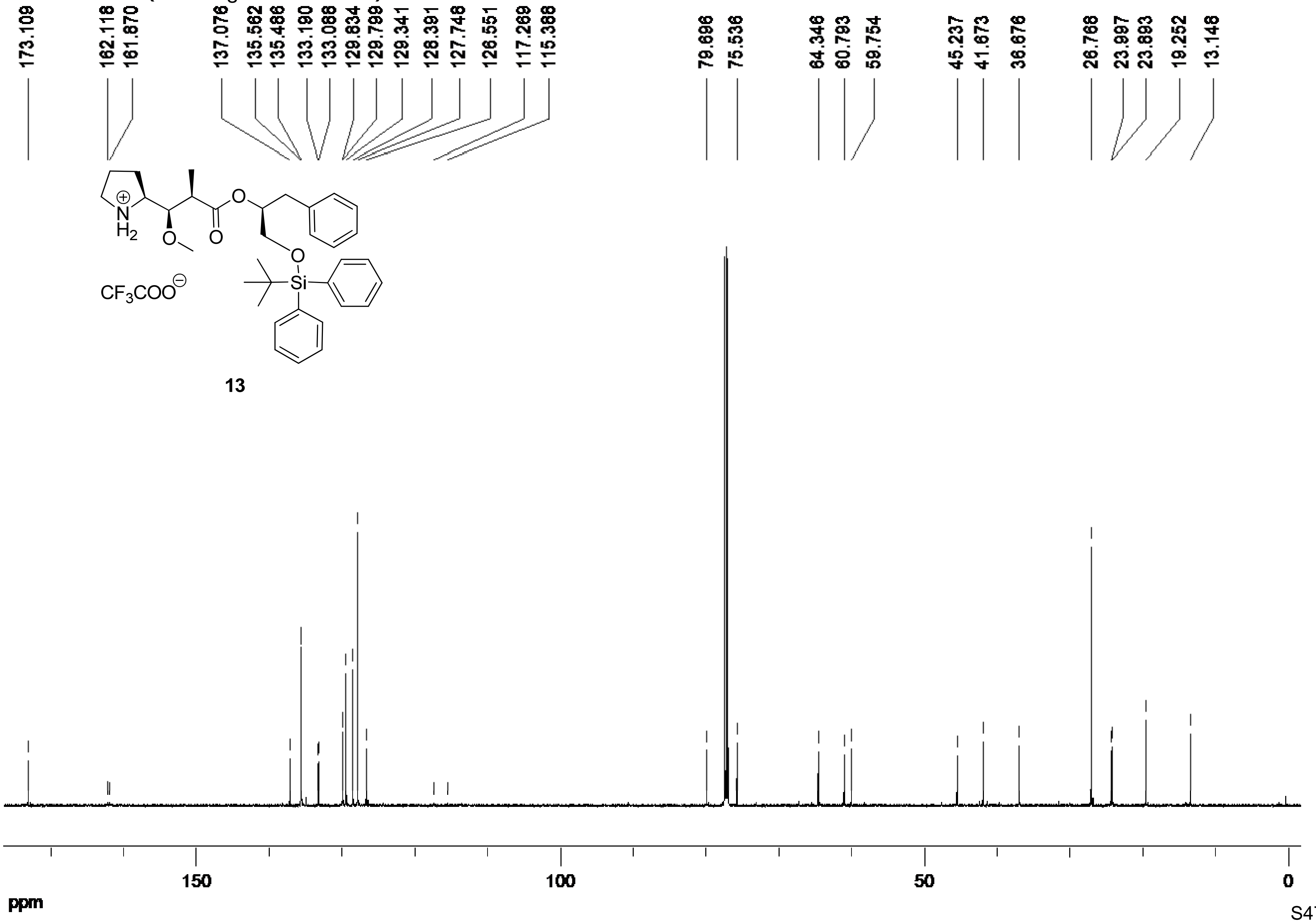

13

TBDPS-malevamide D (14)

^{1}H NMR ($CDCl_3$, 600 MHz)

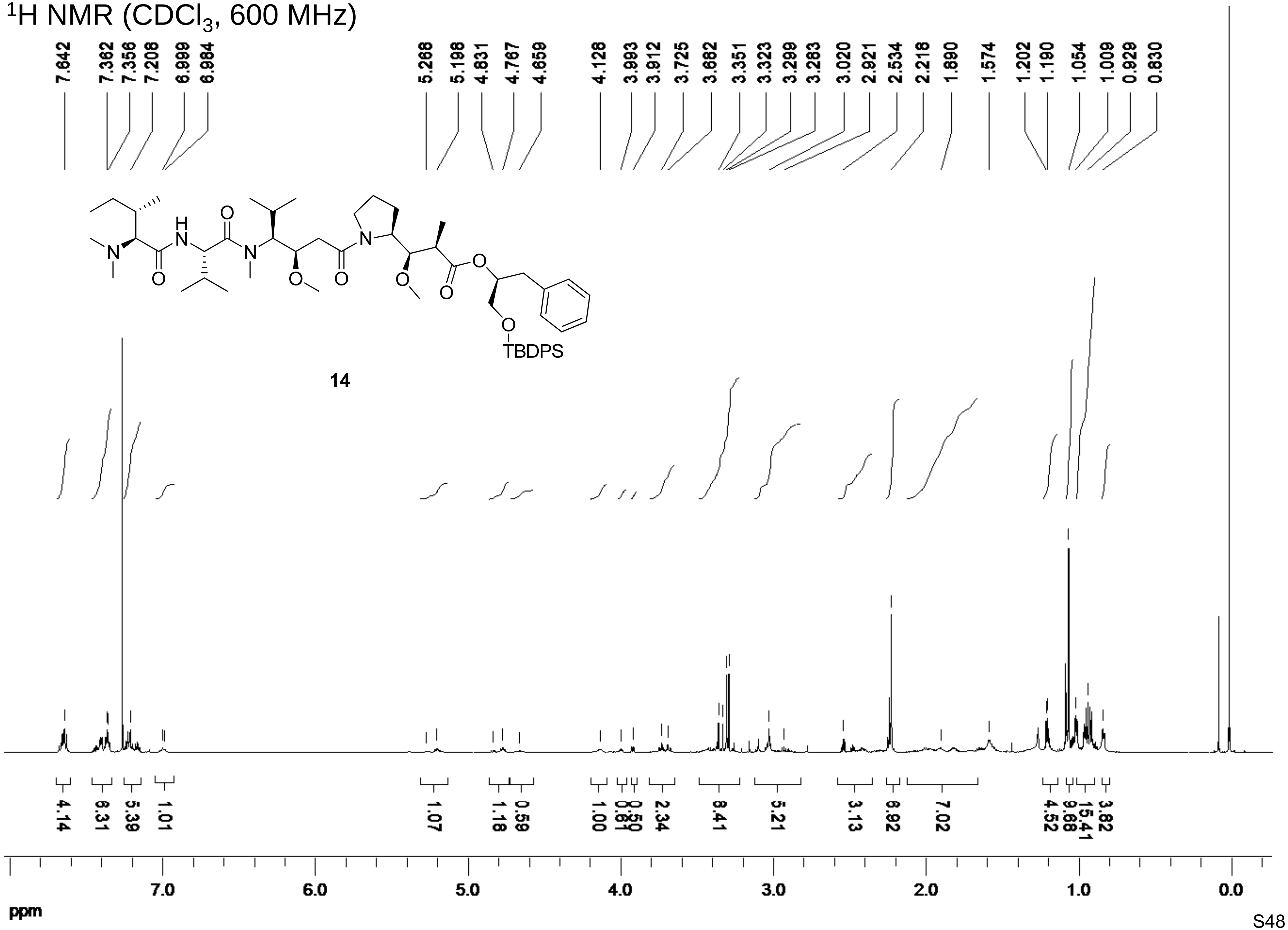

TBDPS-malevamide D (14)

^{13}C NMR ($CDCl_3$, 150 MHz)

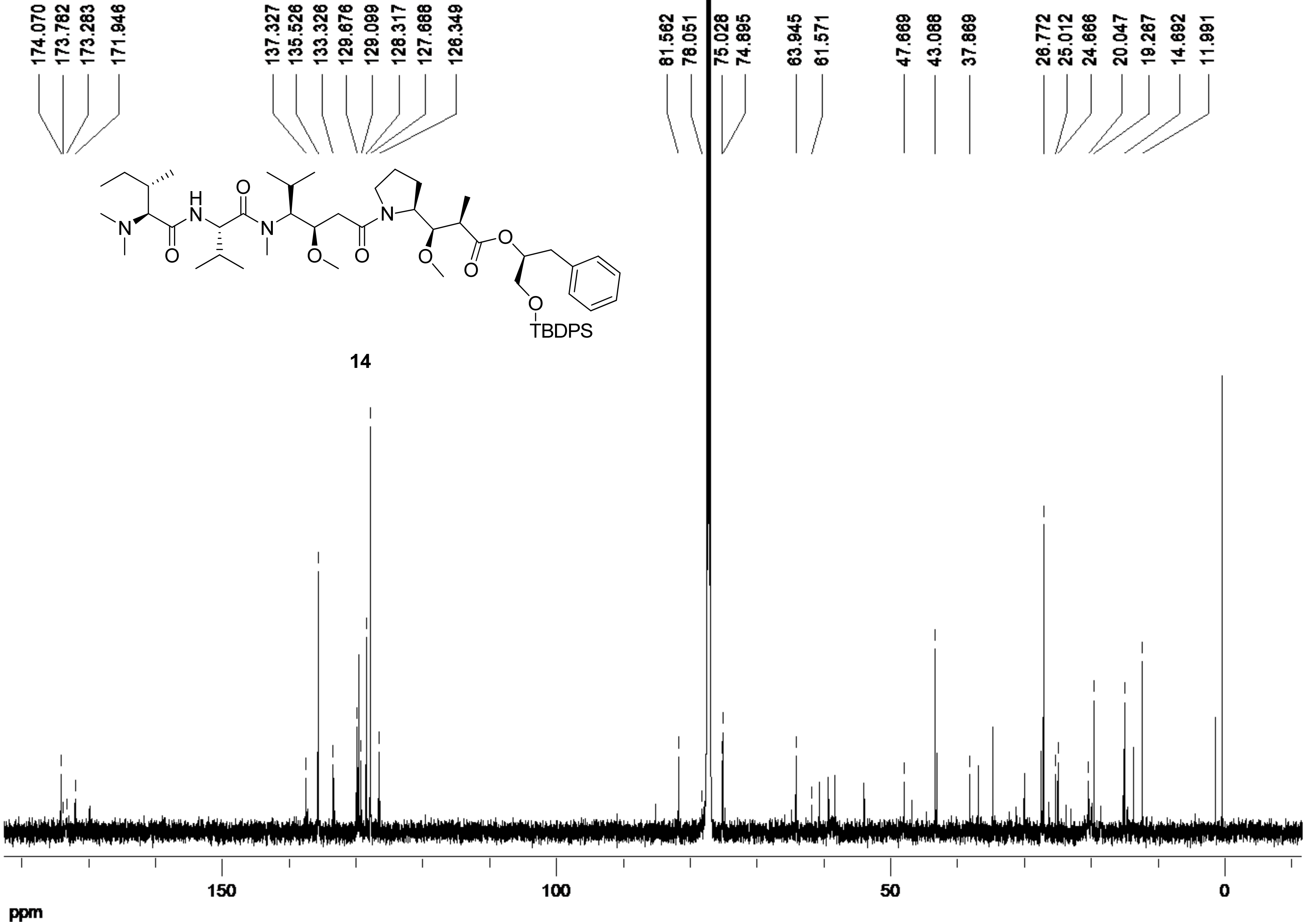

Malevamide D (1)

^{1}H NMR ($CDCl_3$, 600 MHz)

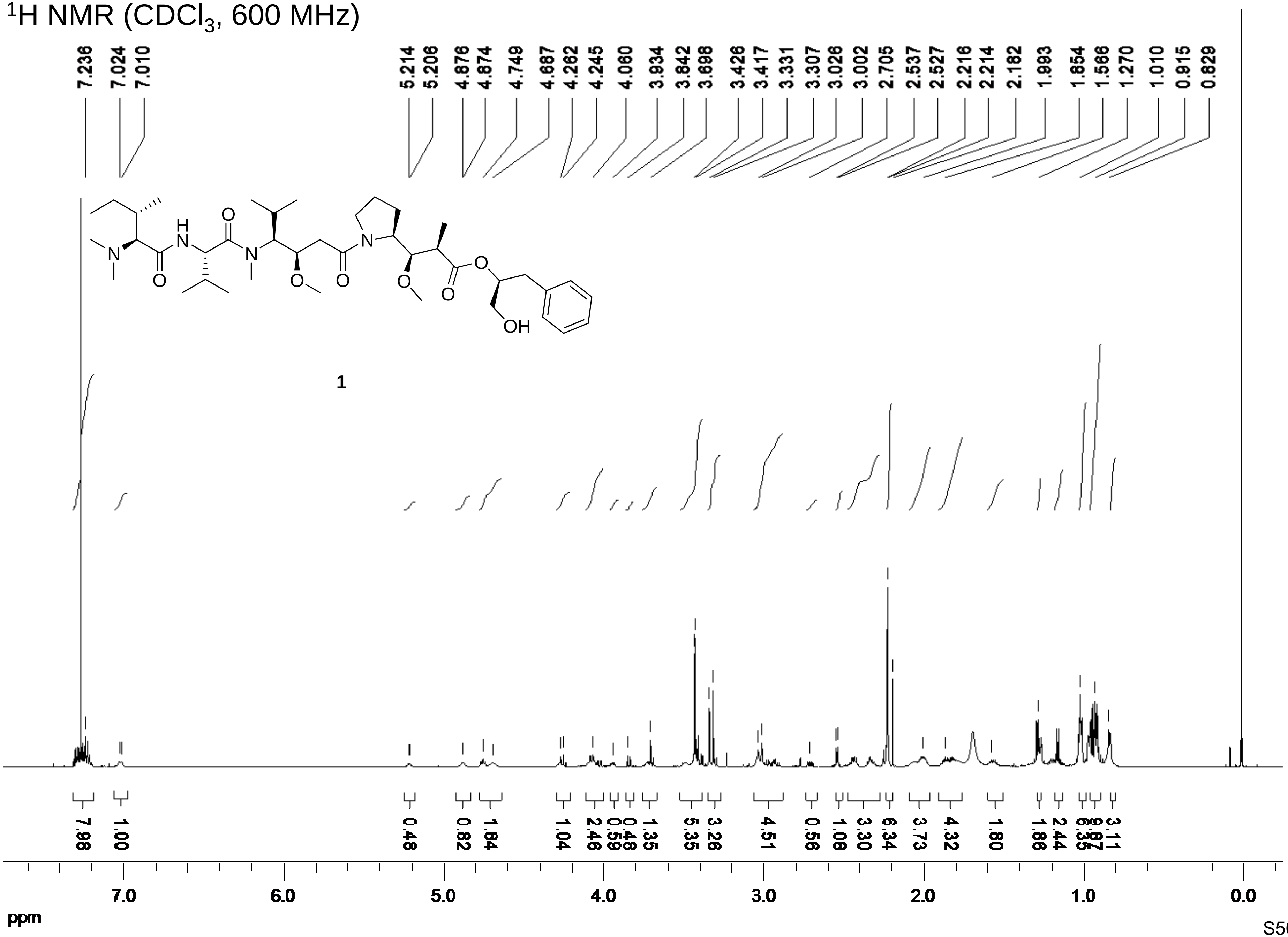

Malevamide D (1)

^{13}C NMR ($CDCl_3$, 150 MHz)

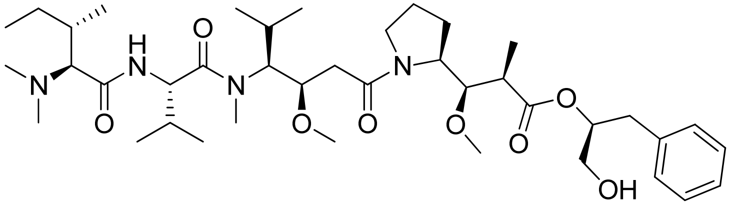

1

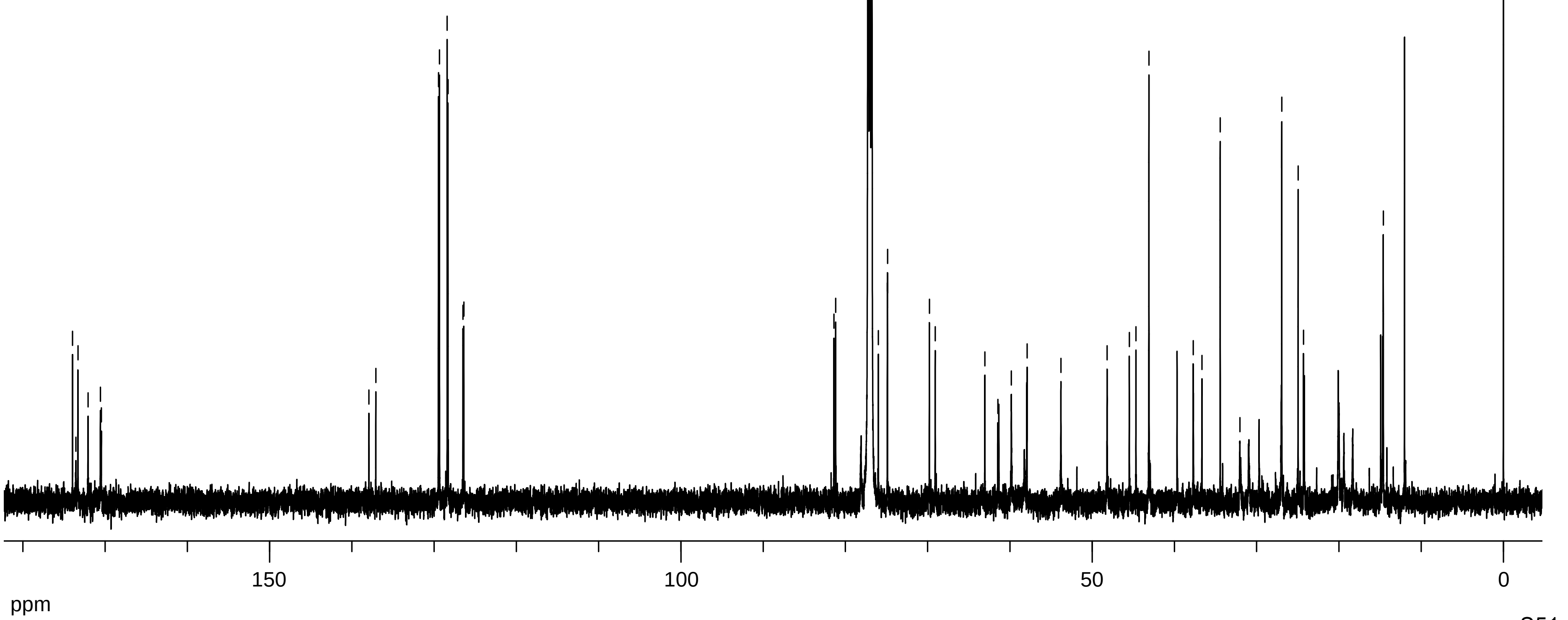

2-((1*S*,2*S*)-1-(Dimethylamino)-2-methylbutyl)-4-isopropyloxazol-5-yl diethylphosphate (18), ^{1}H NMR ($CDCl_3$, 400 MHz)

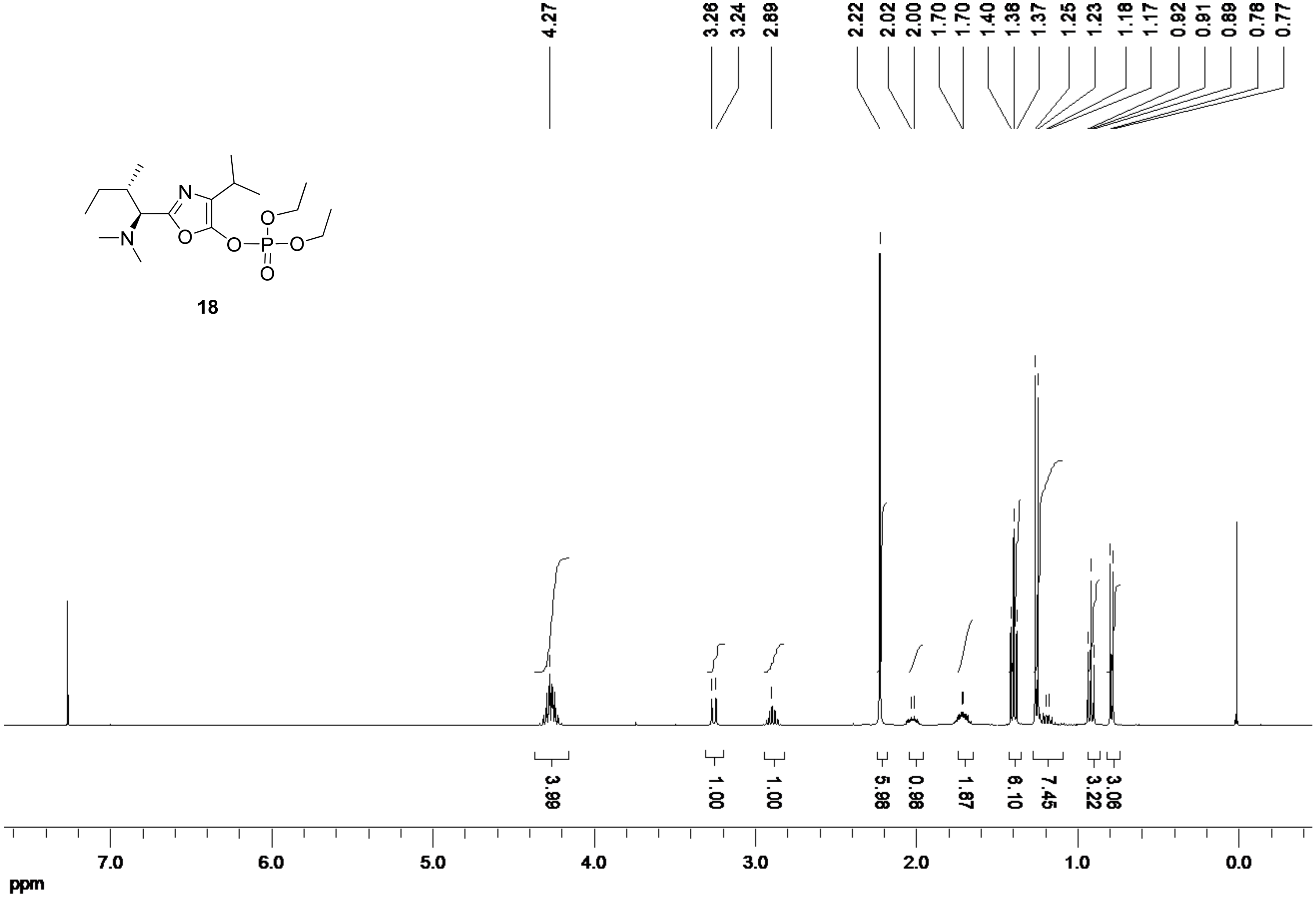

2-((1*S*,2*S*)-1-(Dimethylamino)-2-methylbutyl)-4-isopropyloxazol-5-yl diethylphosphate (18), ^{13}C NMR (CDCl$_3$, 100 MHz)

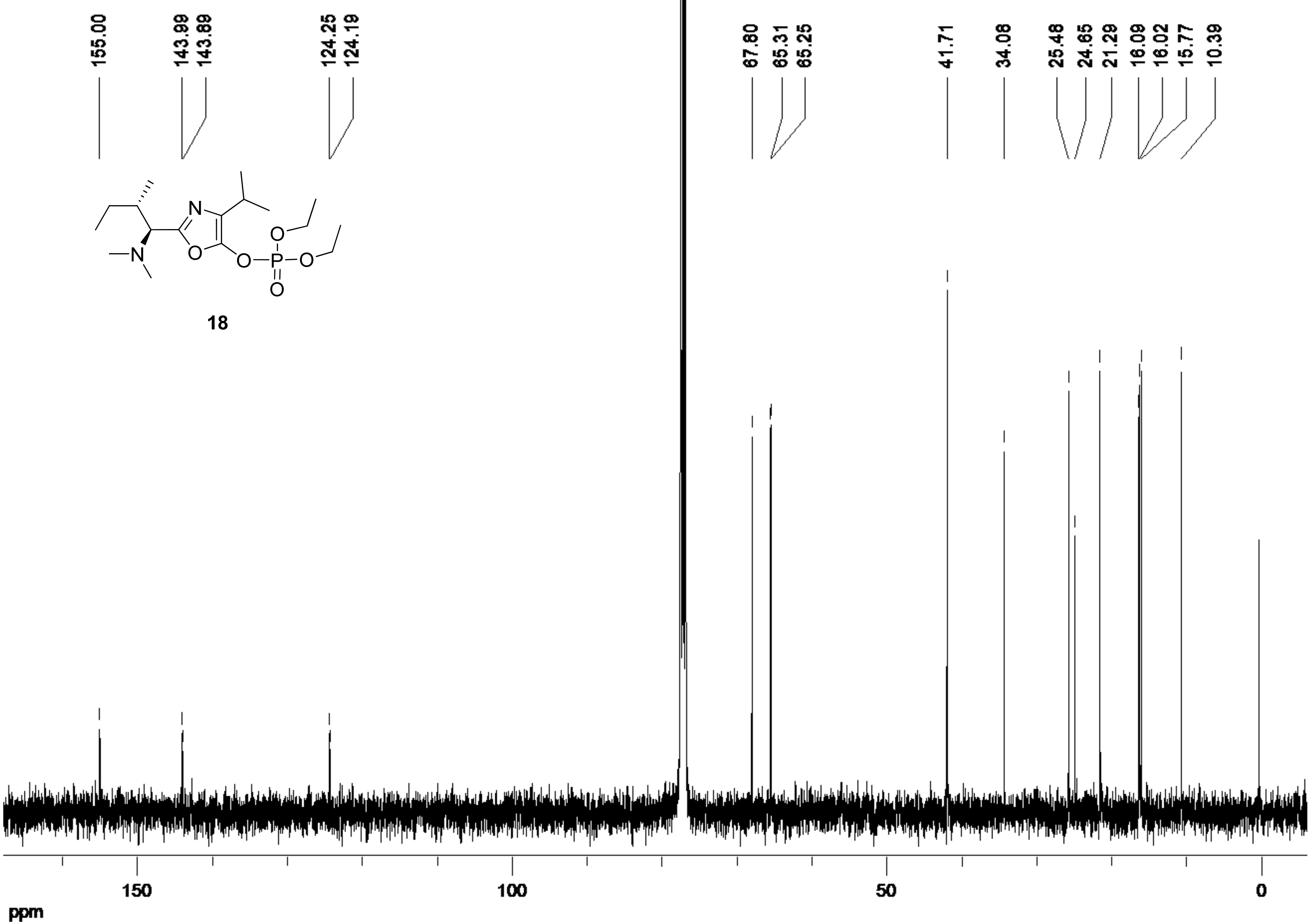

1-(4-(2,3-Dihydroxypropyl)phenyl)-2,2,2-trifluoroethanone oxime (20)

^{1}H NMR ($CDCl_3$, 400 MHz)

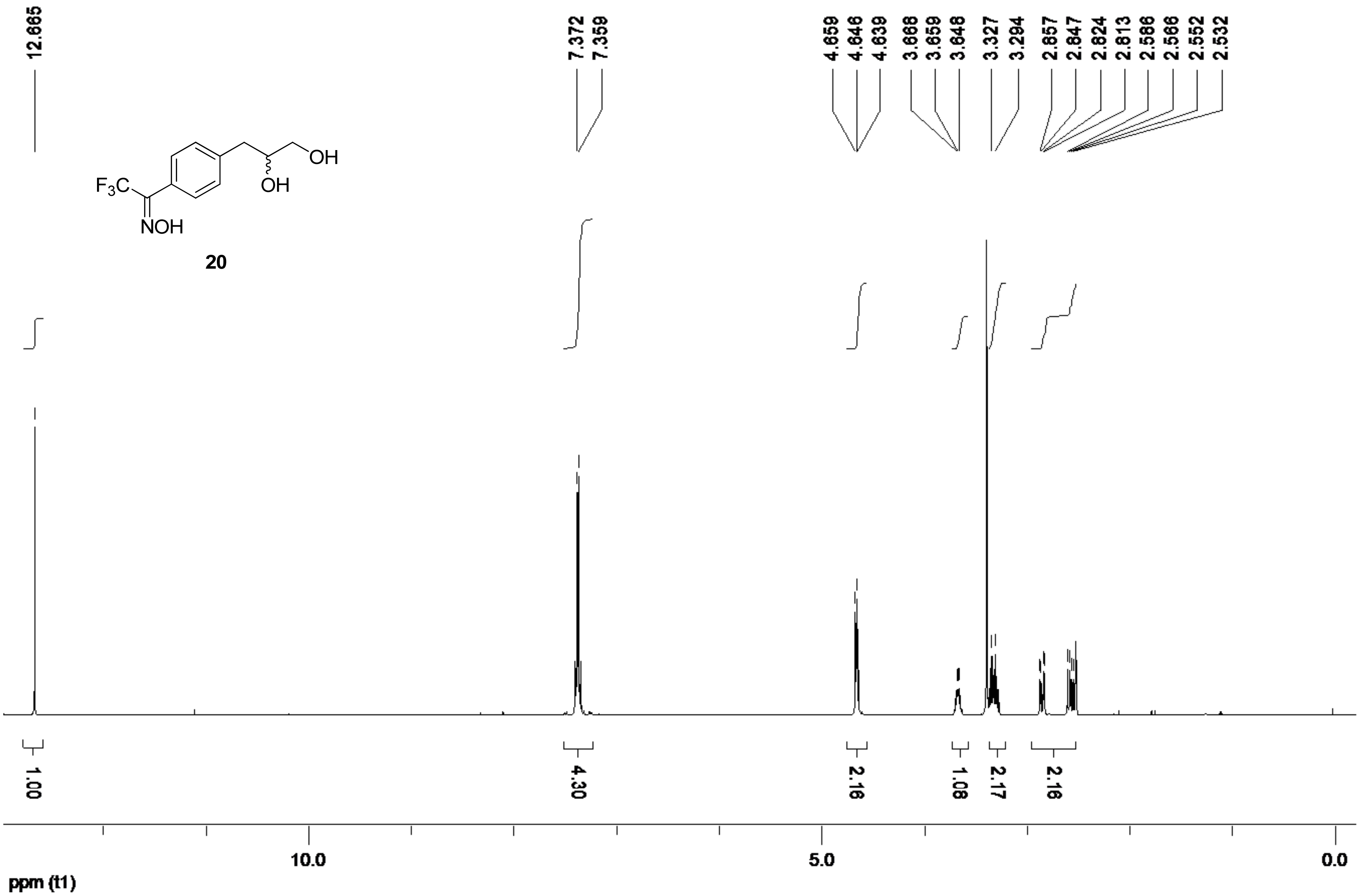

1-(4-(2,3-Dihydroxypropyl)phenyl)-2,2,2-trifluoroethanone oxime (20)

^{13}C NMR ($CDCl_3$, 100 MHz)

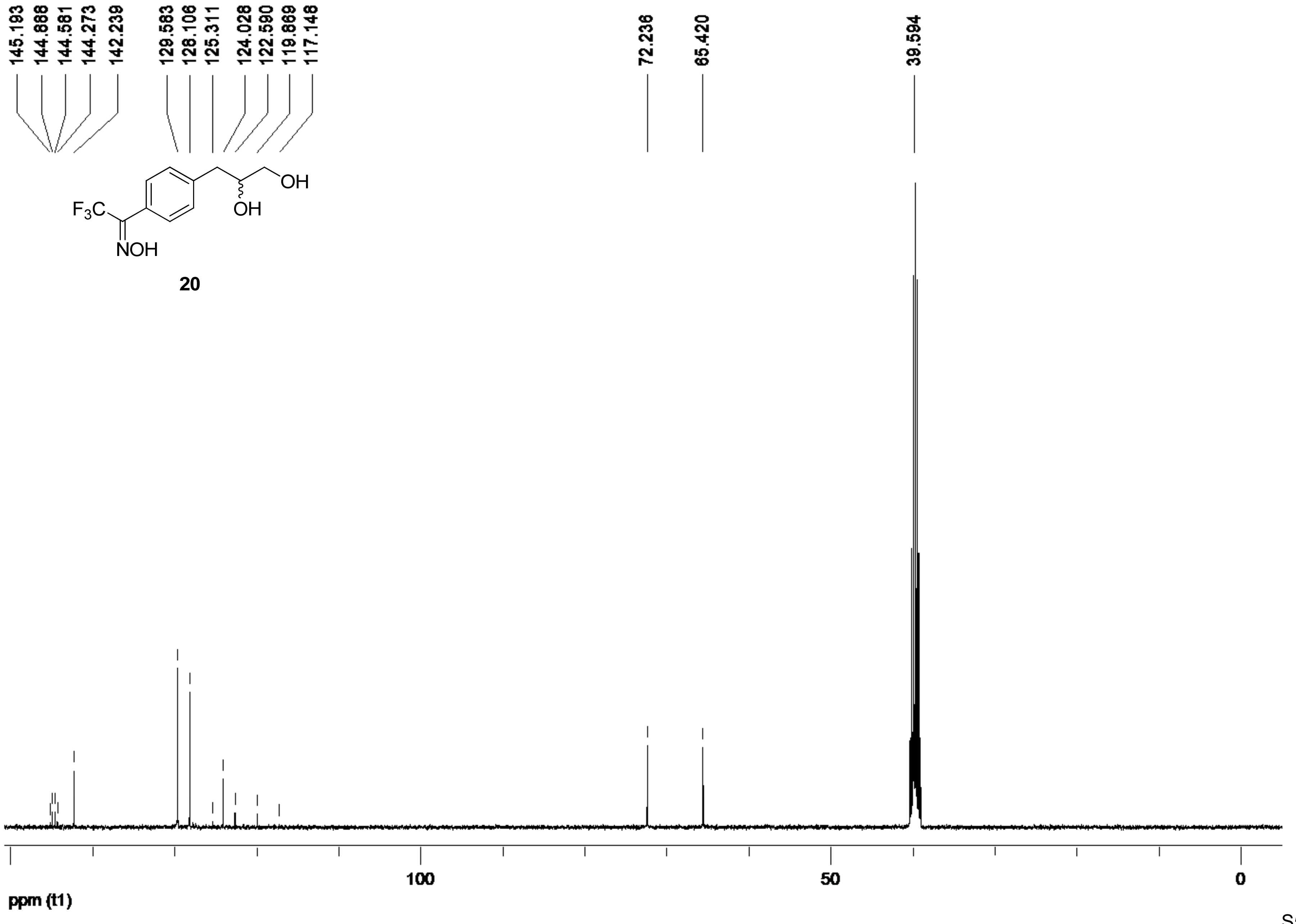

1-(4-((2,2-Dimethyl-1,3-dioxolan-4-yl)methyl)phenyl)-2,2,2-trifluoroethanone oxime (21), ^{1}H NMR ($CDCl_3$, 400 MHz)

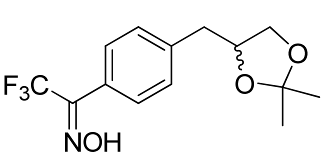

21

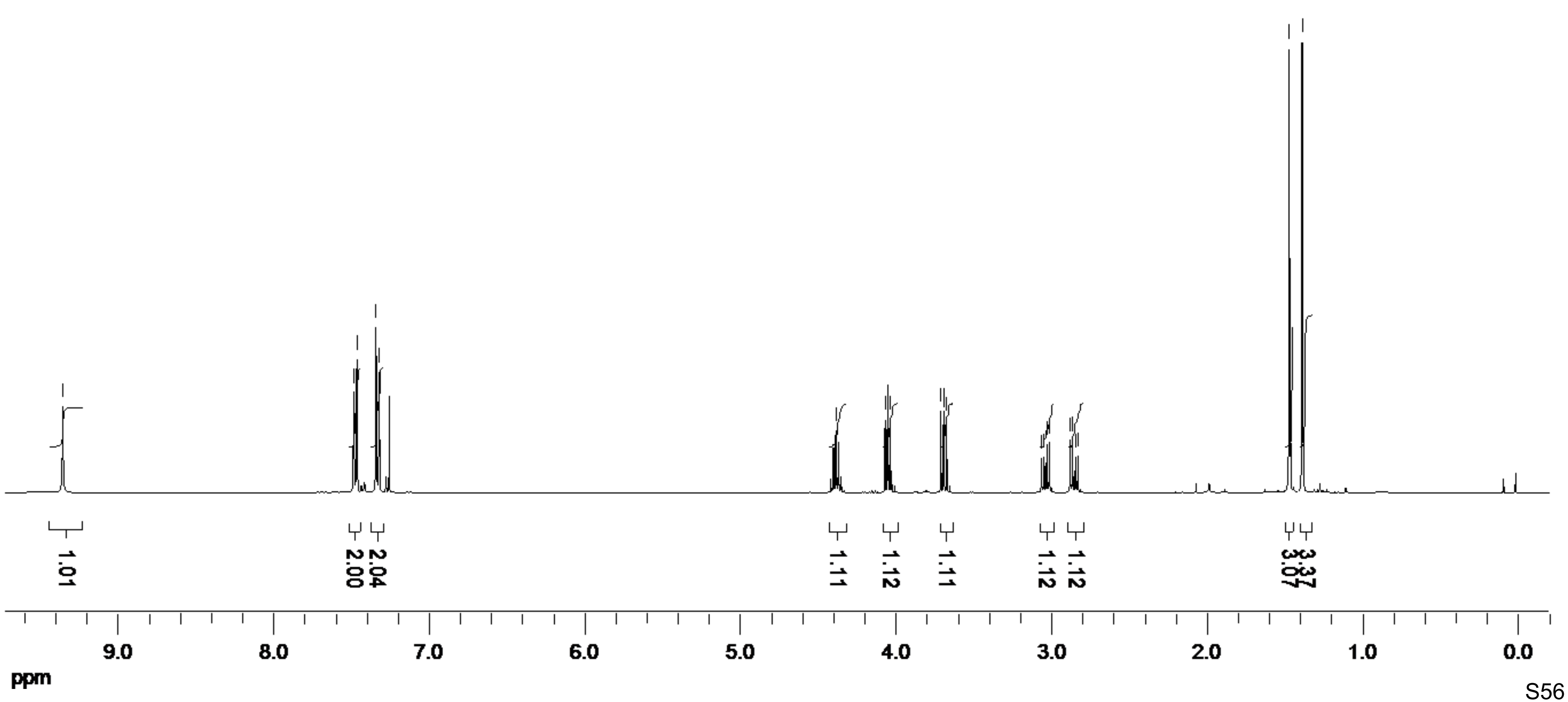

1-(4-((2,2-Dimethyl-1,3-dioxolan-4-yl)methyl)phenyl)-2,2,2-trifluoroethanone oxime (21), ^{13}C NMR ($CDCl_3$, 100 MHz)

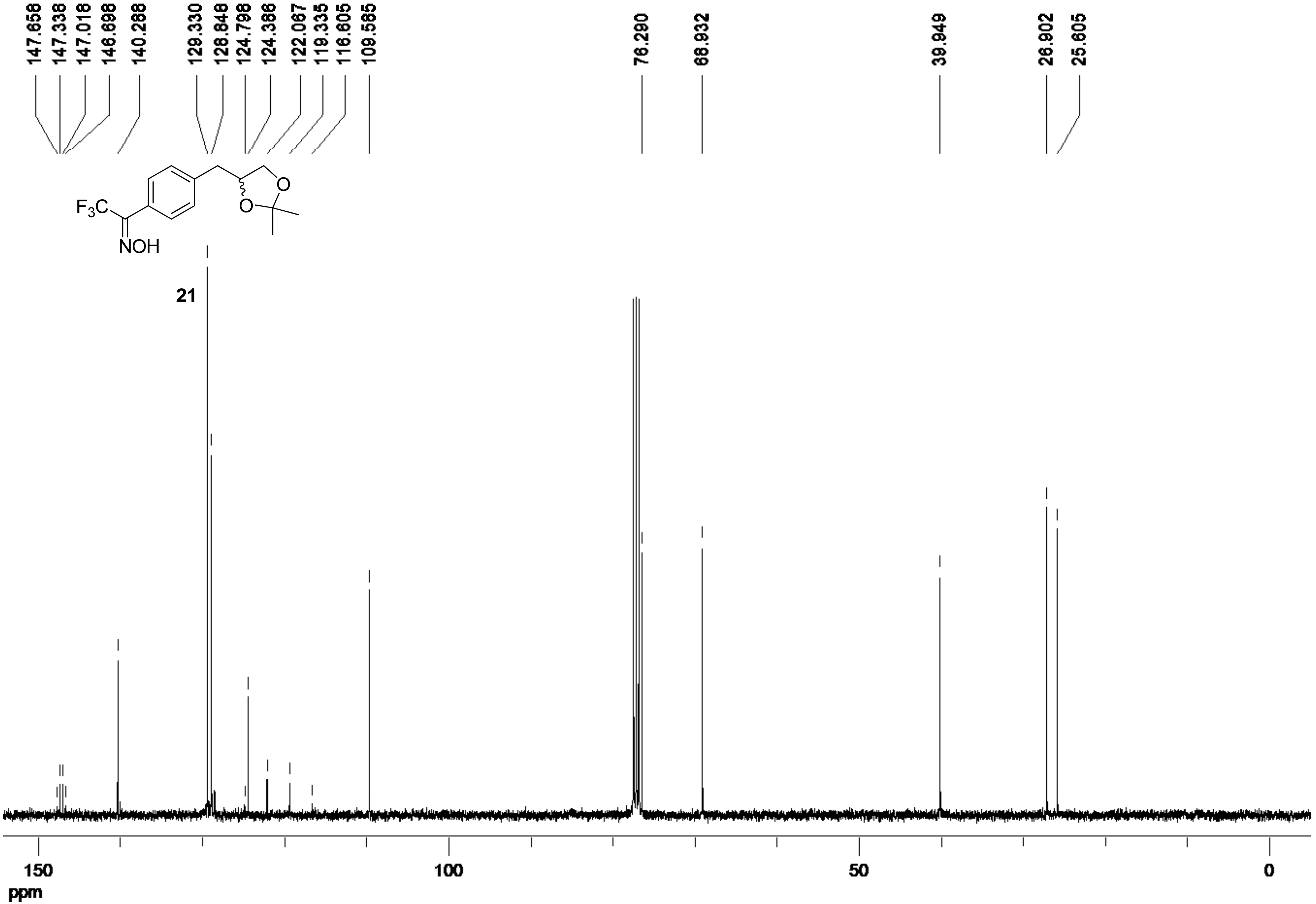

(*E*)-1-(4-((2,2-Dimethyl-1,3-dioxolan-4-yl)methyl)phenyl)-2,2,2-trifluoroethanone O-tosyl oxime (*E*)-(22), ^{1}H NMR ($CDCl_3$, 400 MHz)

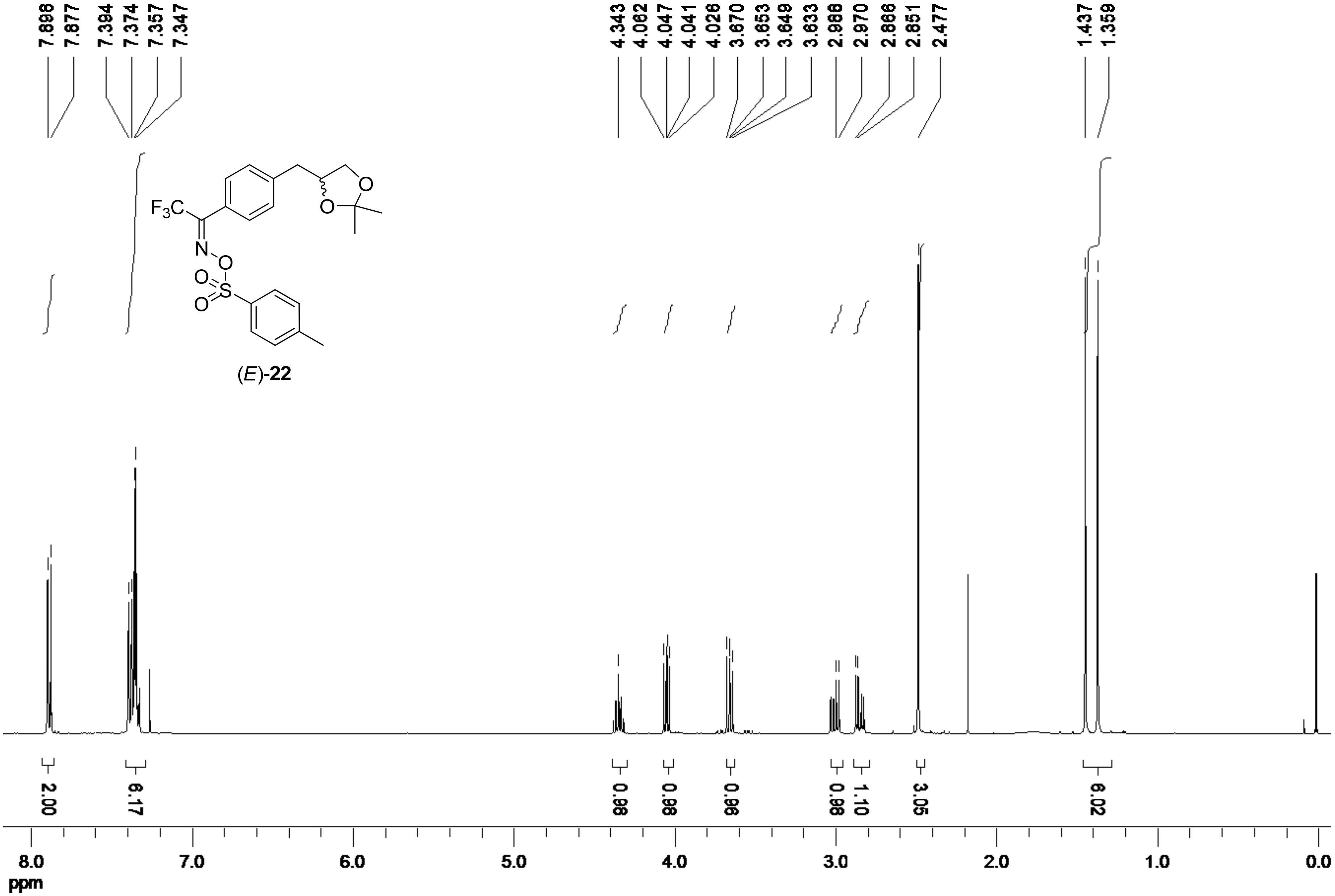

(*E*)-1-(4-((2,2-Dimethyl-1,3-dioxolan-4-yl)methyl)phenyl)-2,2,2-trifluoroethanone O-tosyl oxime (*E*)-(22), ^{13}C NMR ($CDCl_3$, 100 MHz)

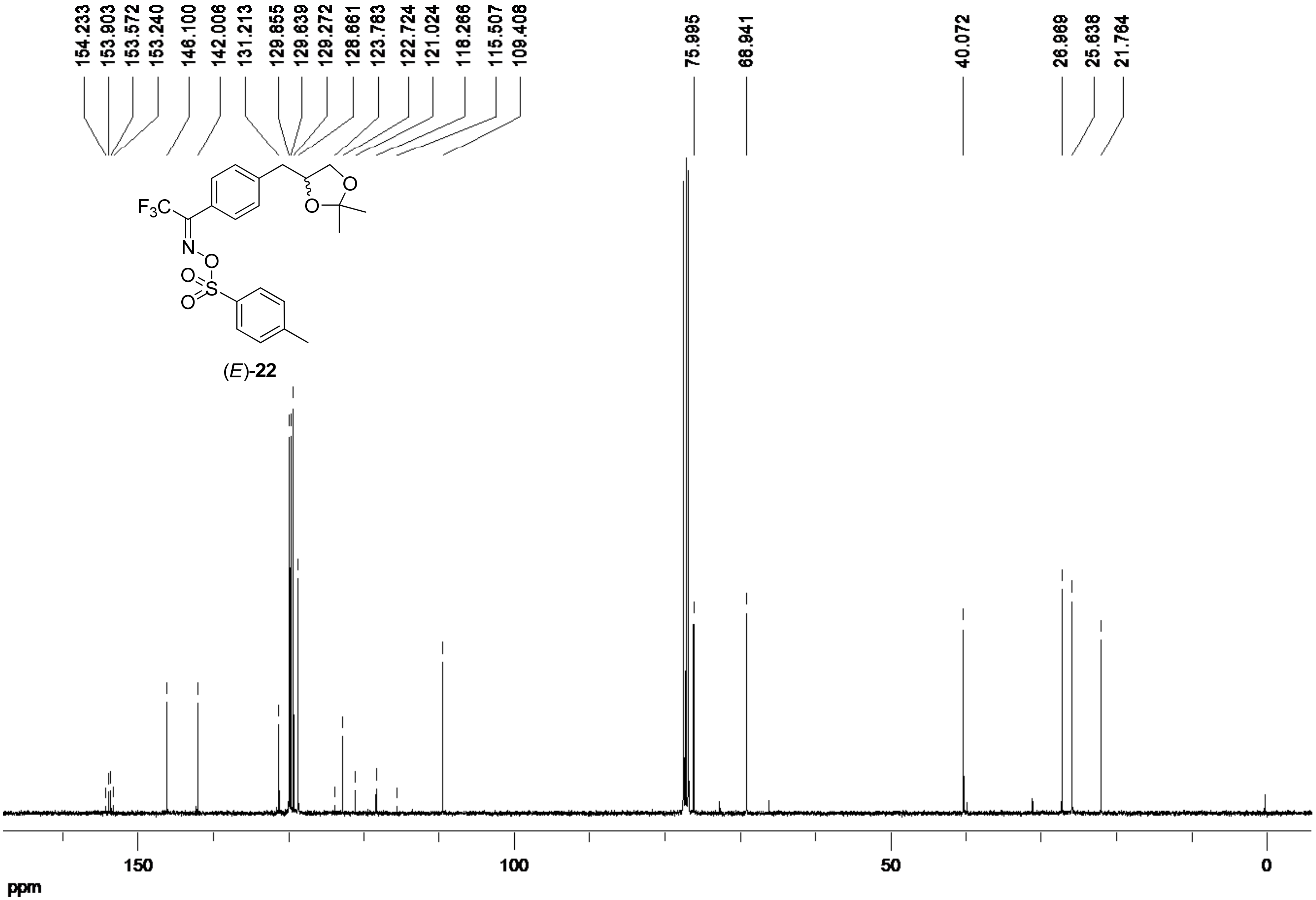

(*Z*)-1-(4-((2,2-Dimethyl-1,3-dioxolan-4-yl)methyl)phenyl)-2,2,2-trifluoroethanone O-tosyl oxime (*Z*)-(22), ^{1}H NMR ($CDCl_3$, 400 MHz)

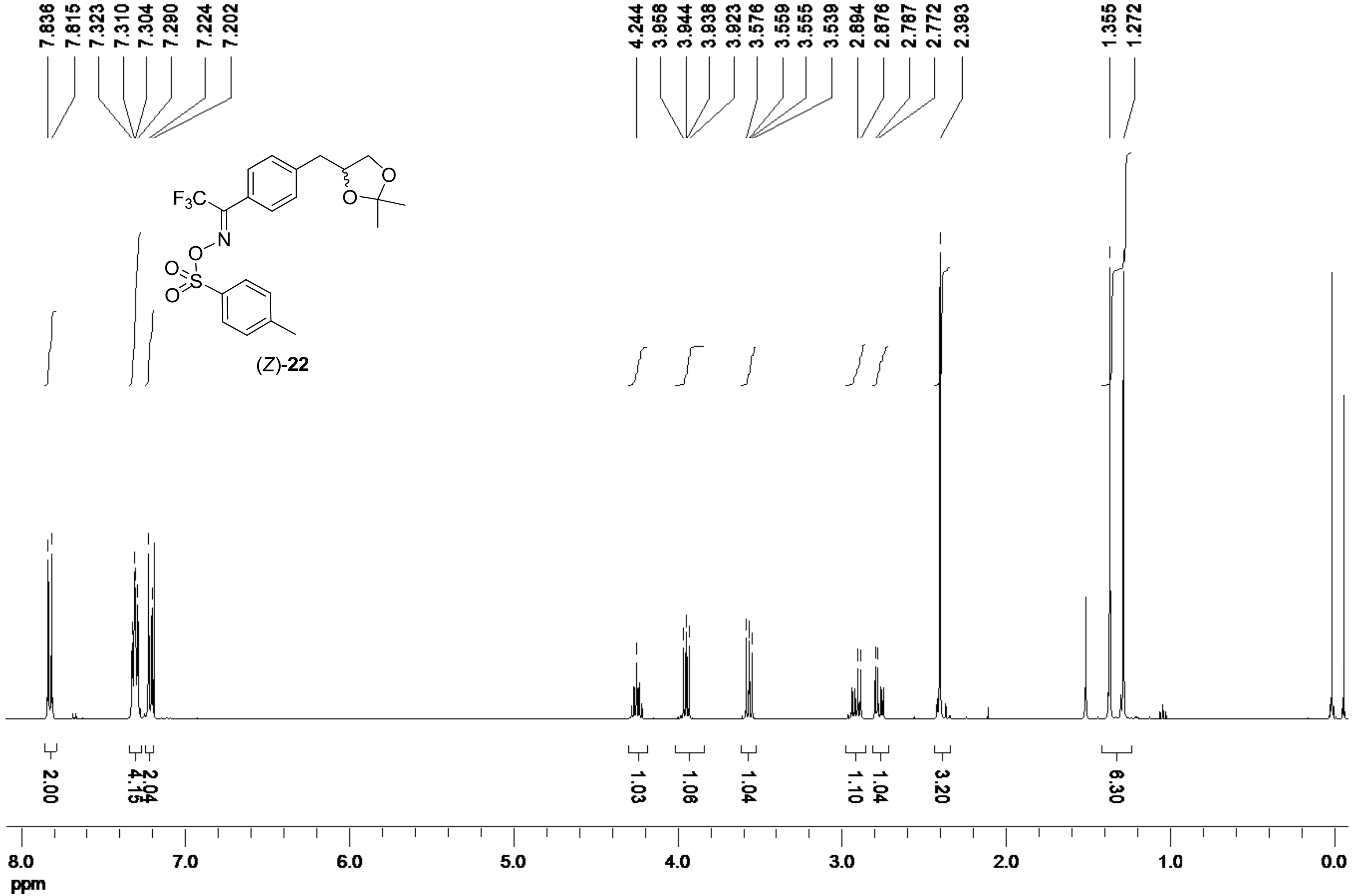

(*Z*)-1-(4-((2,2-Dimethyl-1,3-dioxolan-4-yl)methyl)phenyl)-2,2,2-trifluoroethanone O-tosyl oxime (*Z*)-(22), ^{13}C NMR ($CDCl_3$, 100 MHz)

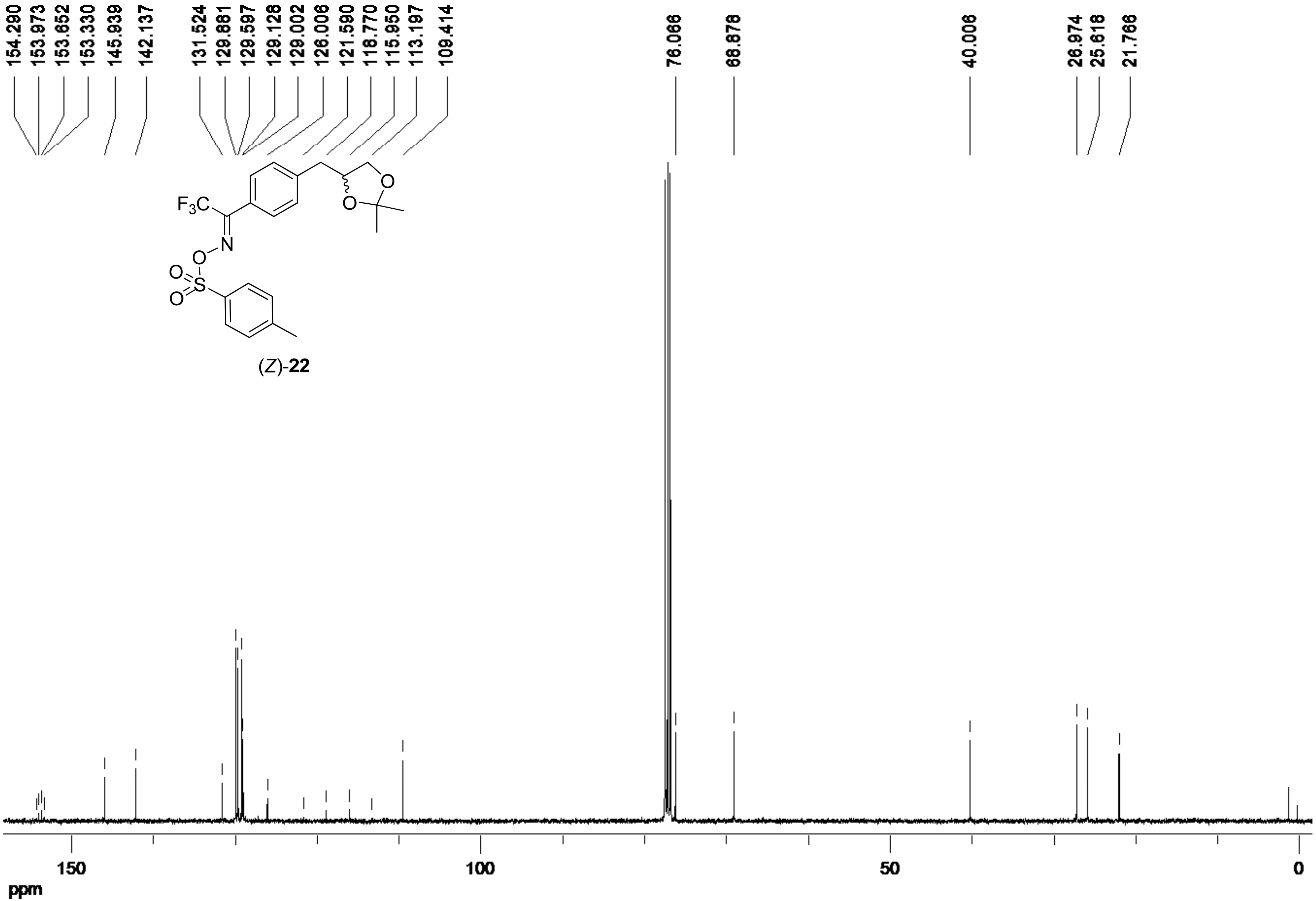

3-(4-((2,2-Dimethyl-1,3-dioxolan-4-yl)methyl)phenyl)-3-(trifluoromethyl)diaziridine (23)

^{1}H NMR ($CDCl_3$, 400 MHz)

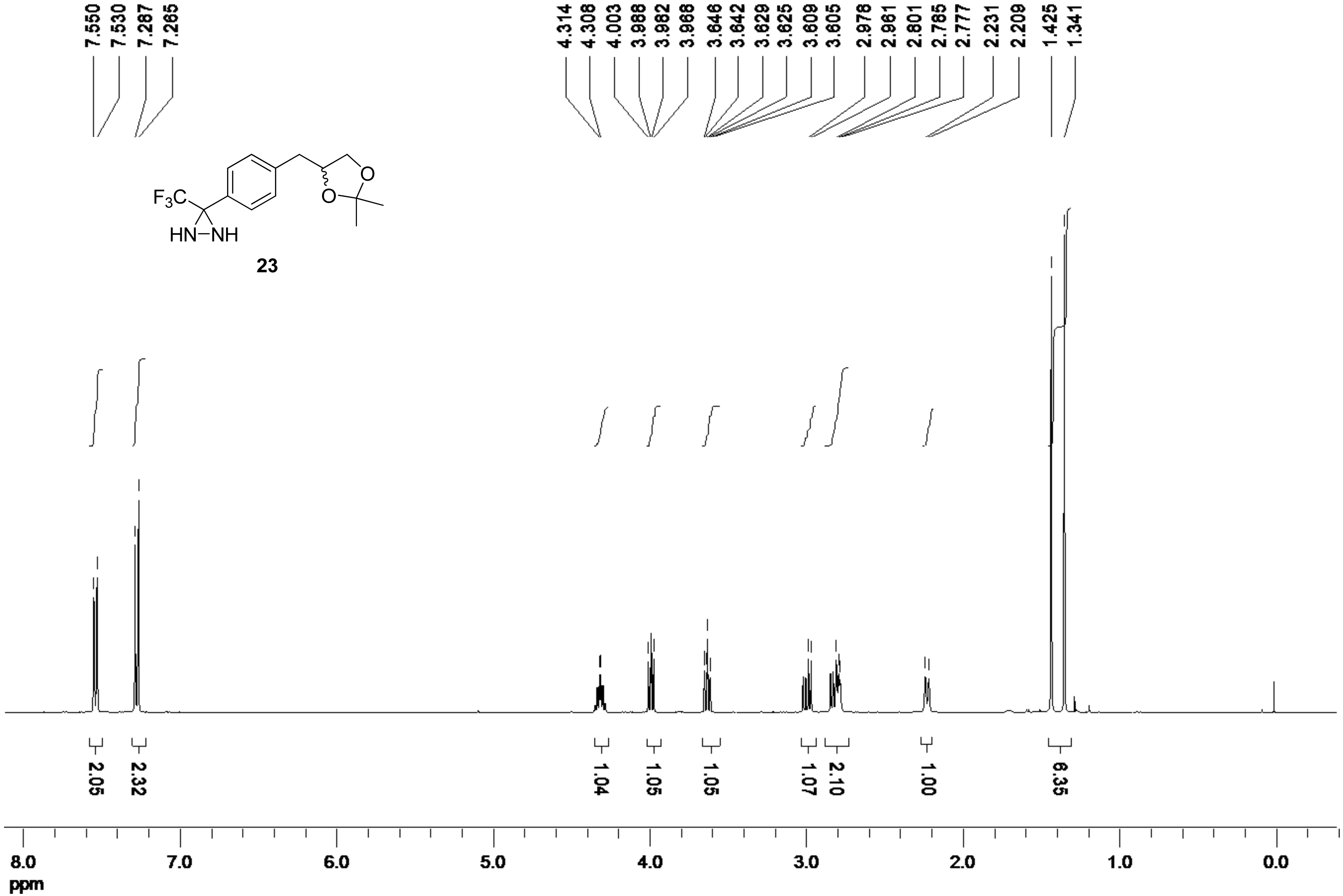

3-(4-((2,2-Dimethyl-1,3-dioxolan-4-yl)methyl)phenyl)-3-(trifluoromethyl)diaziridine (23)

^{13}C NMR ($CDCl_3$, 100 MHz)

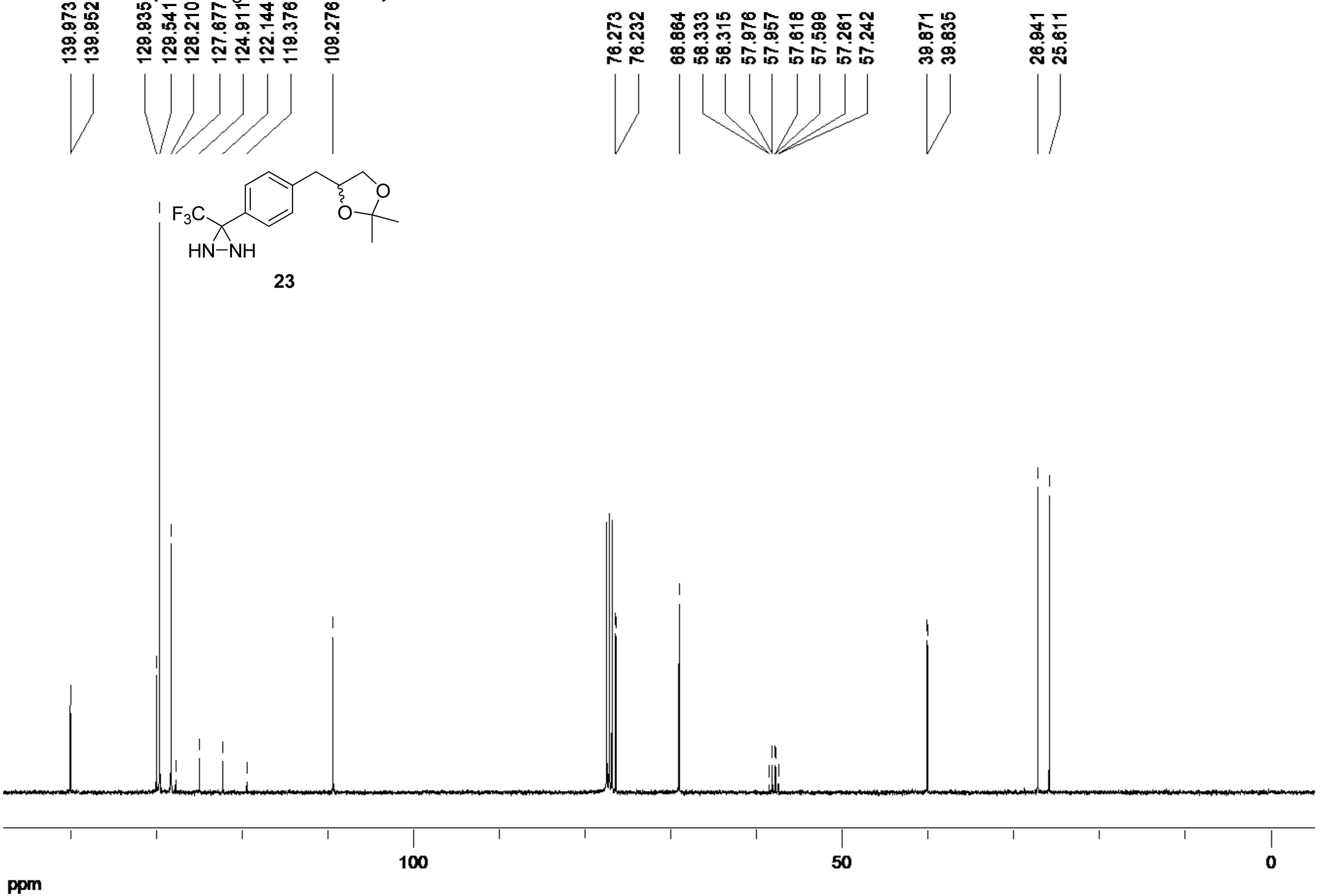

3-(4-((2,2-Dimethyl-1,3-dioxolan-4-yl)methyl)phenyl)-3-(trifluoromethyl)-3*H*-diazirine (24), ^{1}H NMR ($CDCl_3$, 400 MHz)

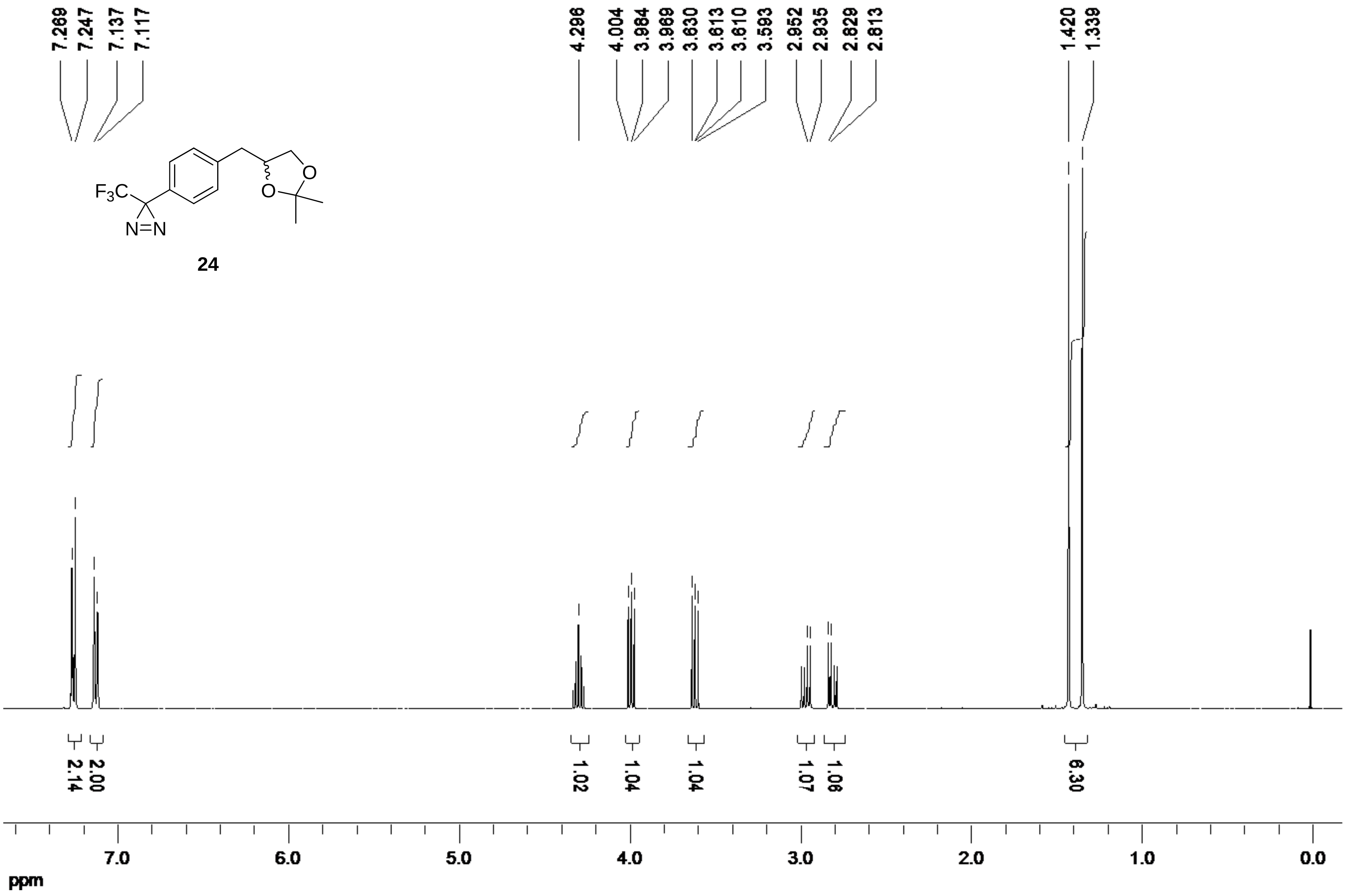

3-(4-((2,2-Dimethyl-1,3-dioxolan-4-yl)methyl)phenyl)-3-(trifluoromethyl)-3*H*-diazirine (24), ^{13}C NMR ($CDCl_3$, 100 MHz)

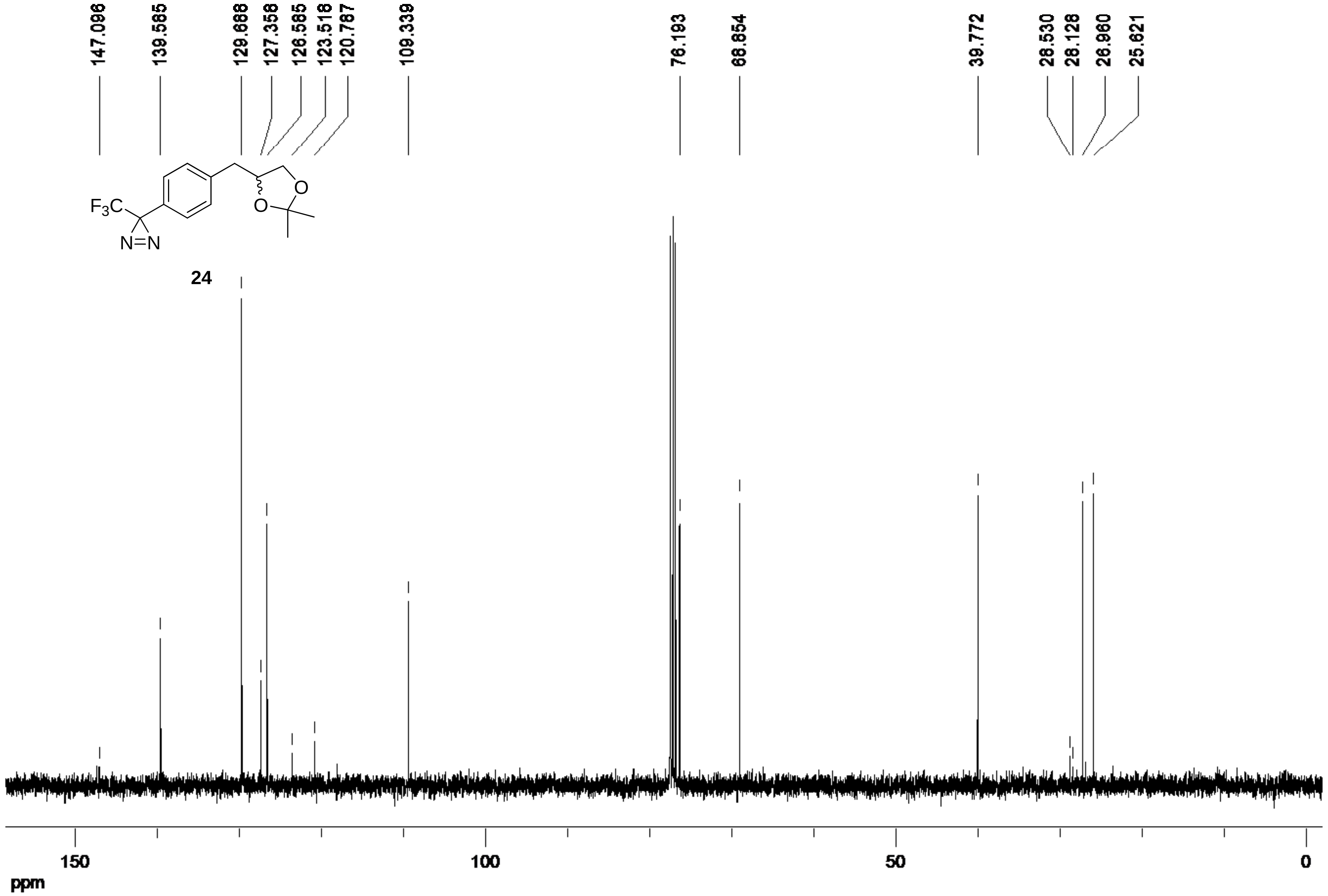

3-(4-(3-(Trifluoromethyl)-3*H*-diazirin-3-yl)phenyl)propane-1,2-diol (25)

^{1}H NMR ($CDCl_3$, 400 MHz)

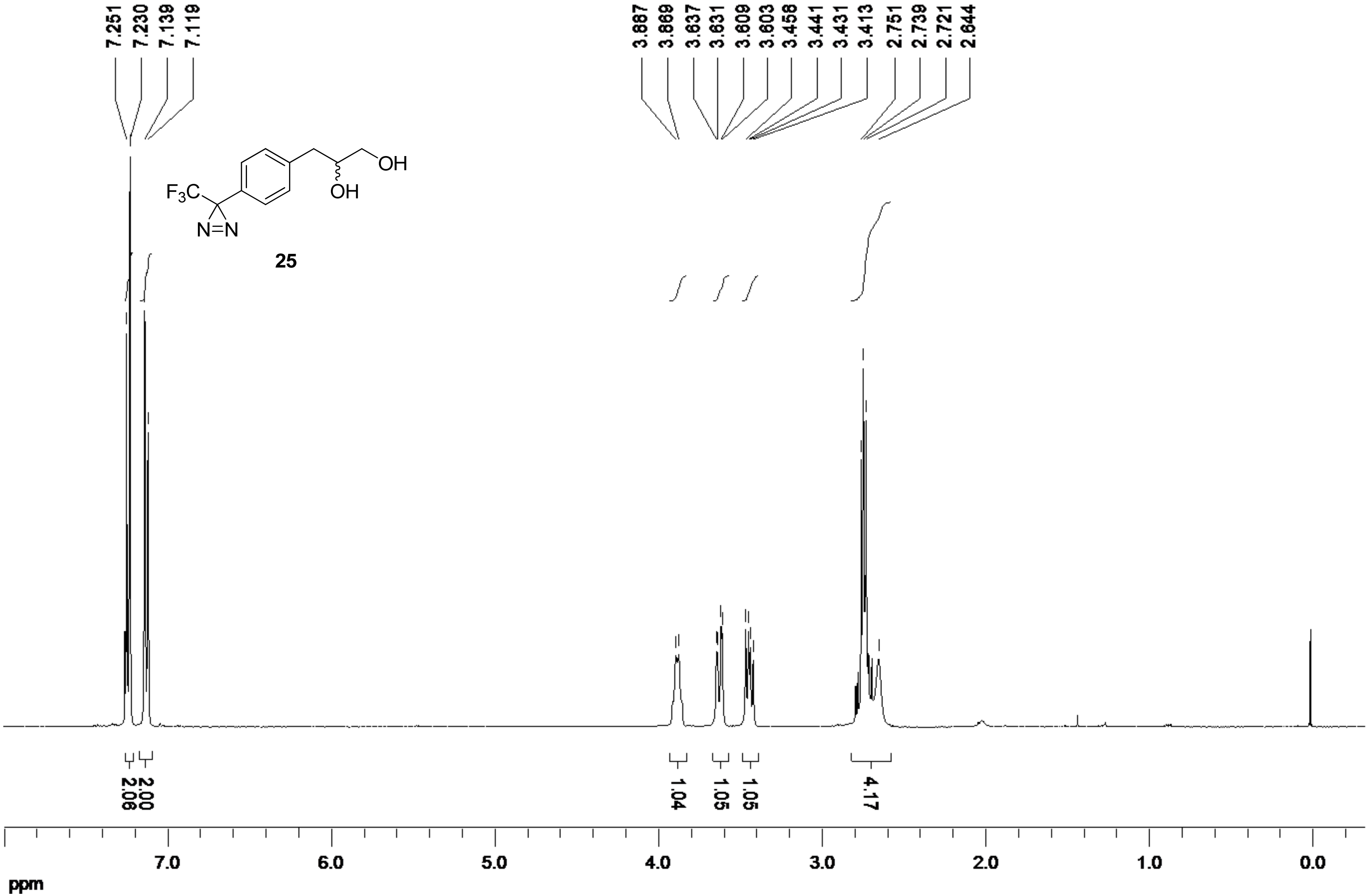

3-(4-(3-(Trifluoromethyl)-3*H*-diazirin-3-yl)phenyl)propane-1,2-diol (25)

^{13}C NMR ($CDCl_3$, 100 MHz)

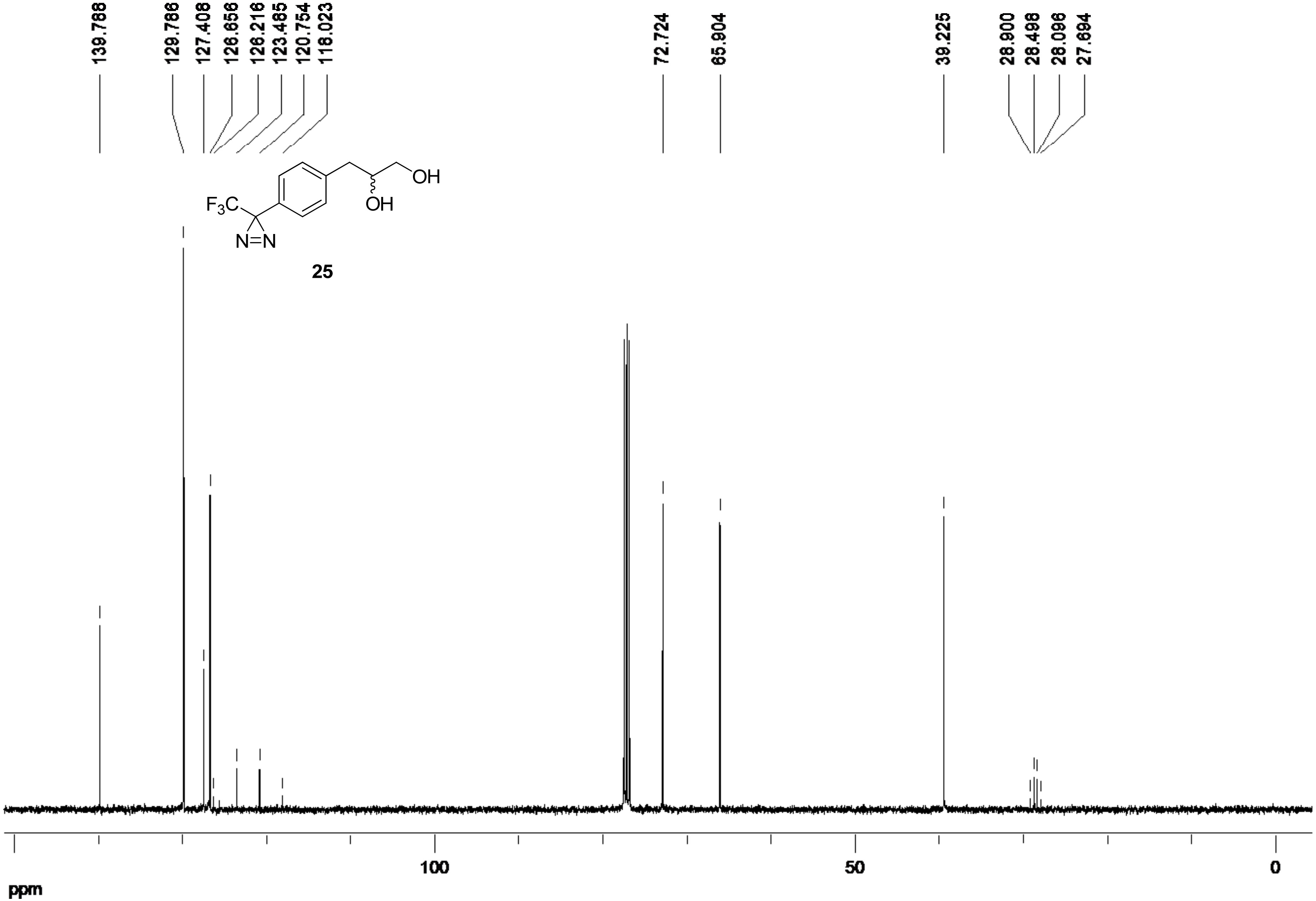

1-TBDPS-oxy-3-(4-(3-(trifluoromethyl)-3*H*-diazirin-3-yl)phenyl)propan-2-ol (26)

^{1}H NMR ($CDCl_3$, 400 MHz)

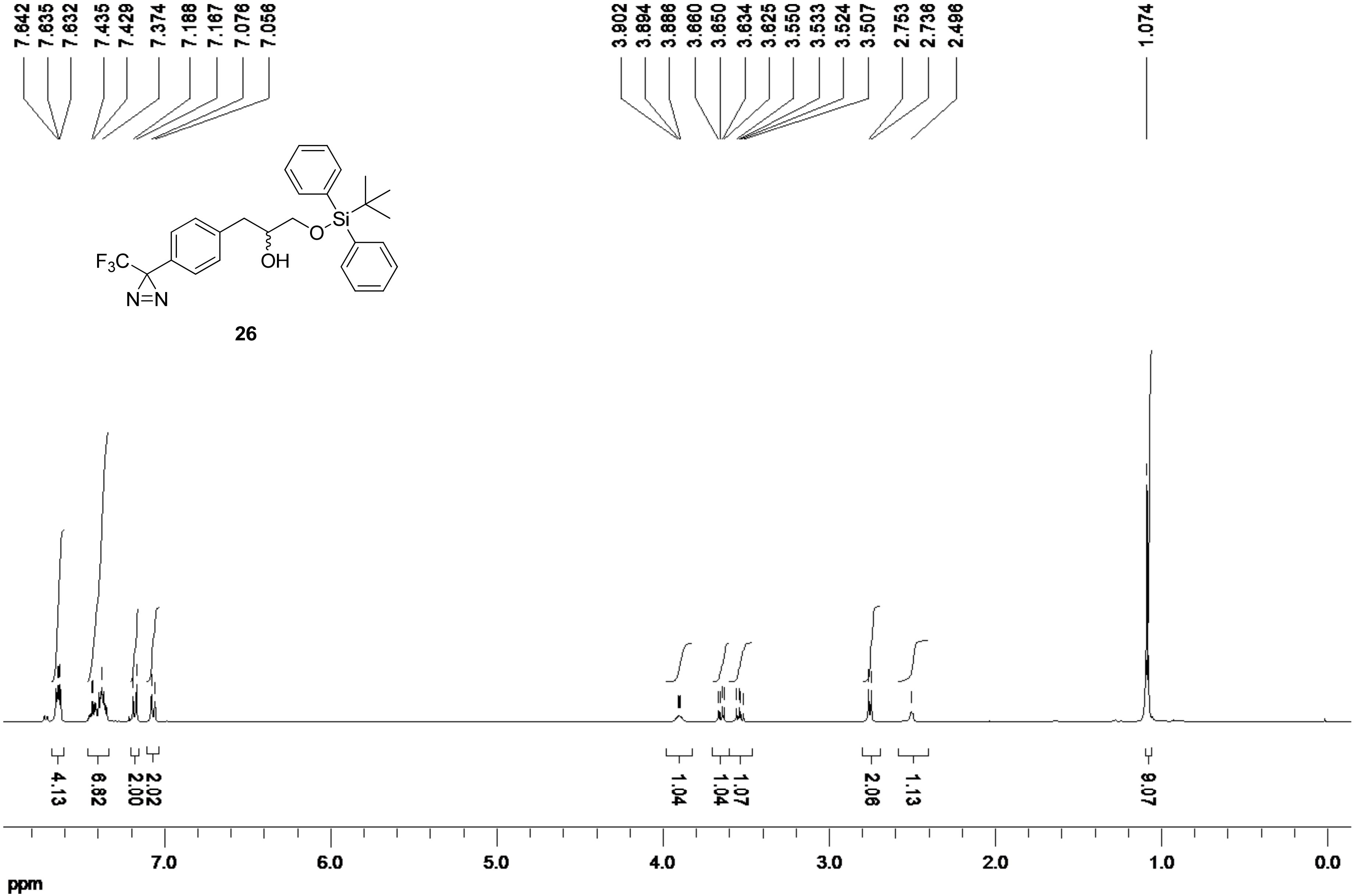

1-TBDPS-oxy-3-(4-(3-(trifluoromethyl)-3*H*-diazirin-3-yl)phenyl)propan-2-ol (26)

^{13}C NMR ($CDCl_3$, 100 MHz)

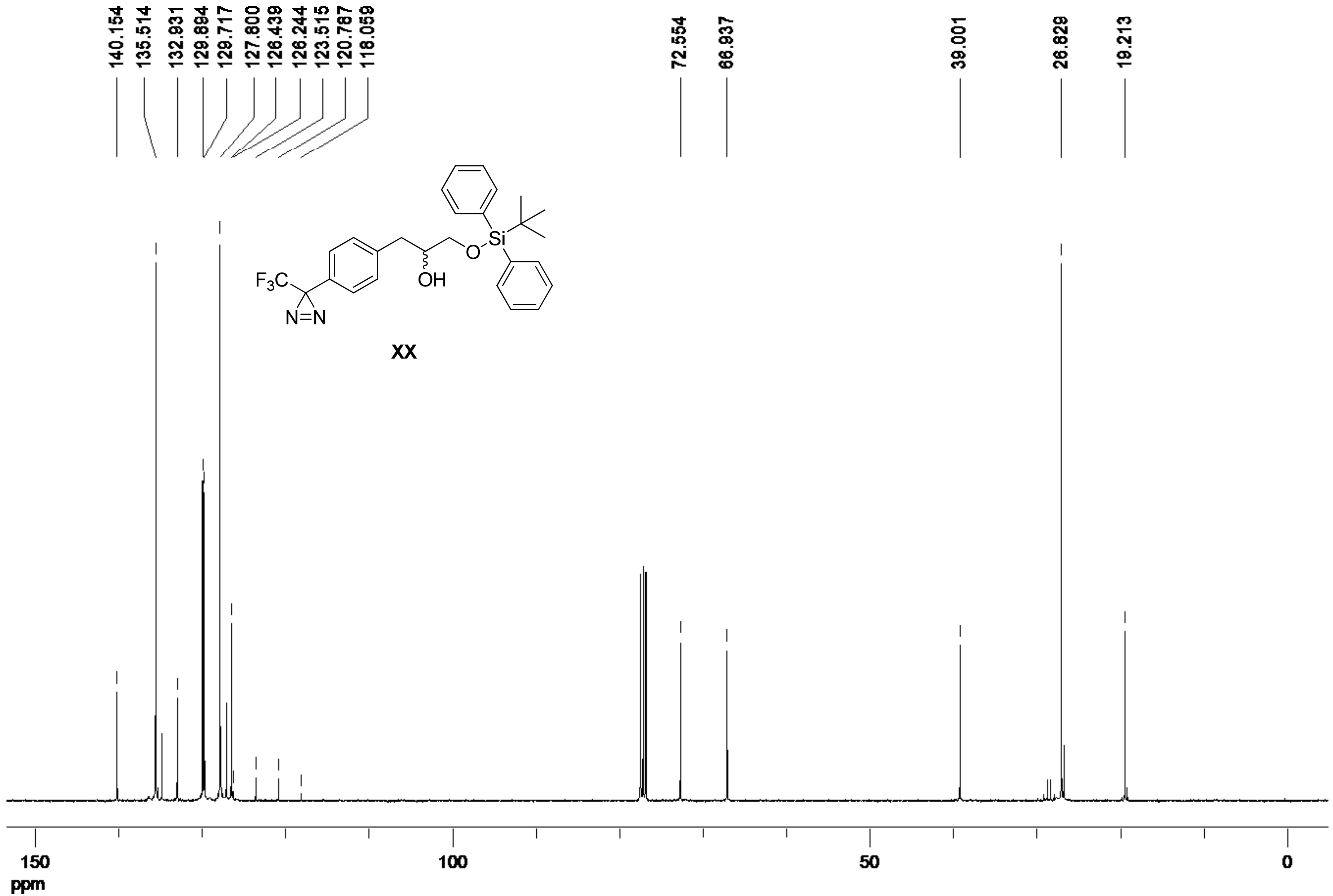

Boc-Dap-*rac*-O[1]TBDPS-Photo-PPD (28)

^{1}H NMR ($CDCl_3$, 400 MHz)

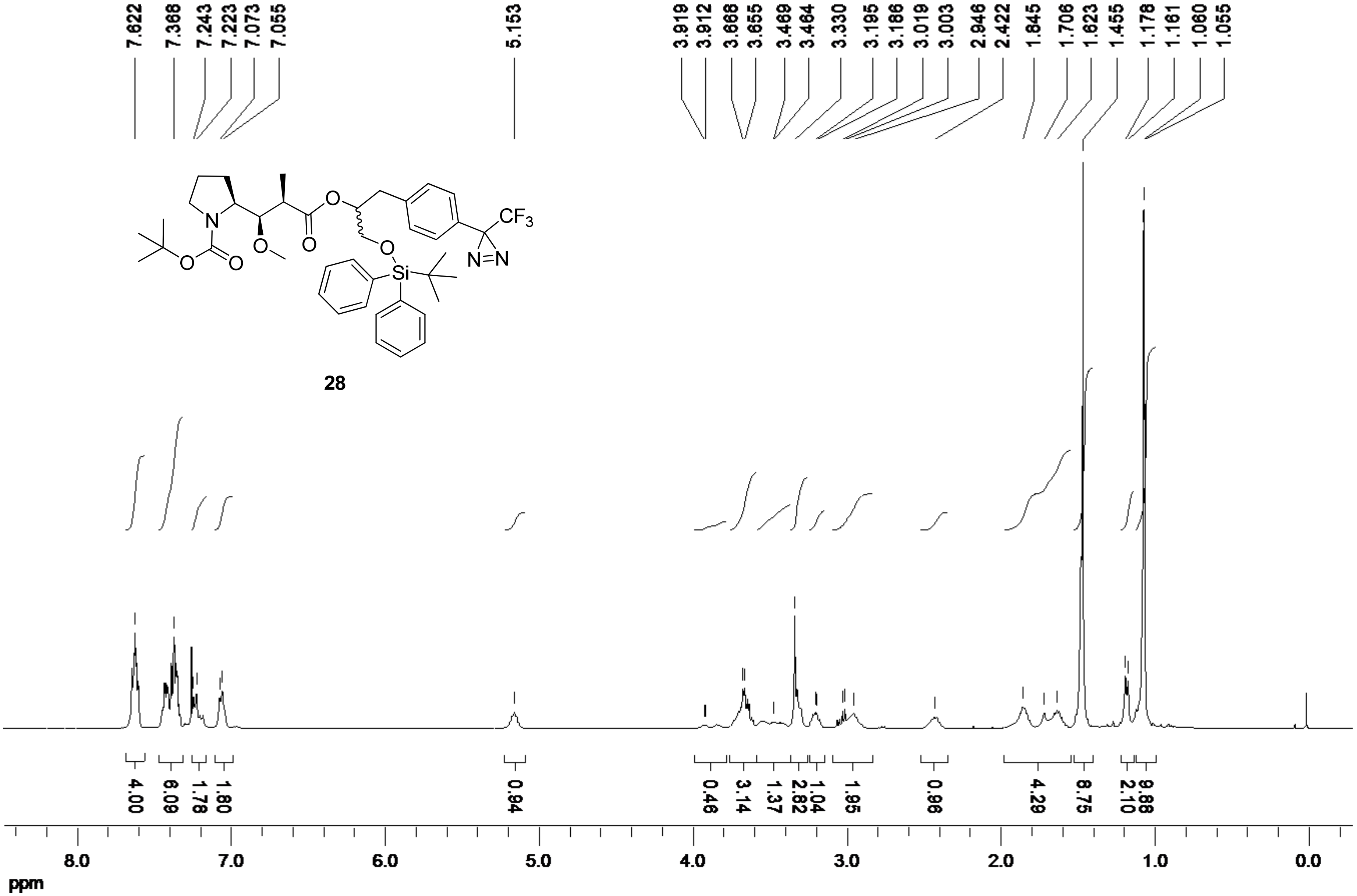

Boc-Dap-*rac*-O¹TBDPS-Photo-PPD (28)

^{13}C NMR ($CDCl_3$, 100 MHz)

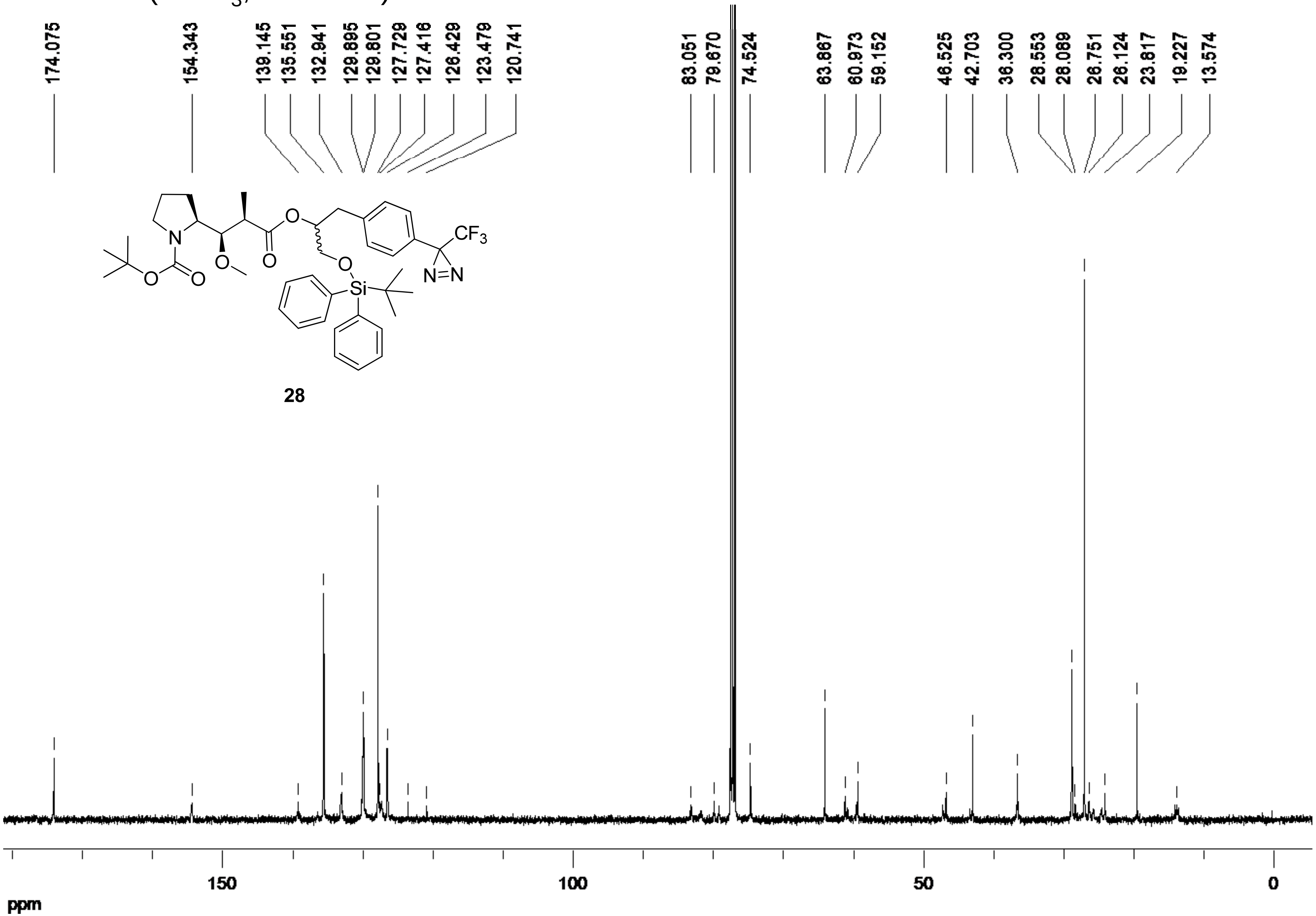

TBDPS-Photo malevamide D (29)

^1H NMR ($CDCl_3$, 600 MHz)

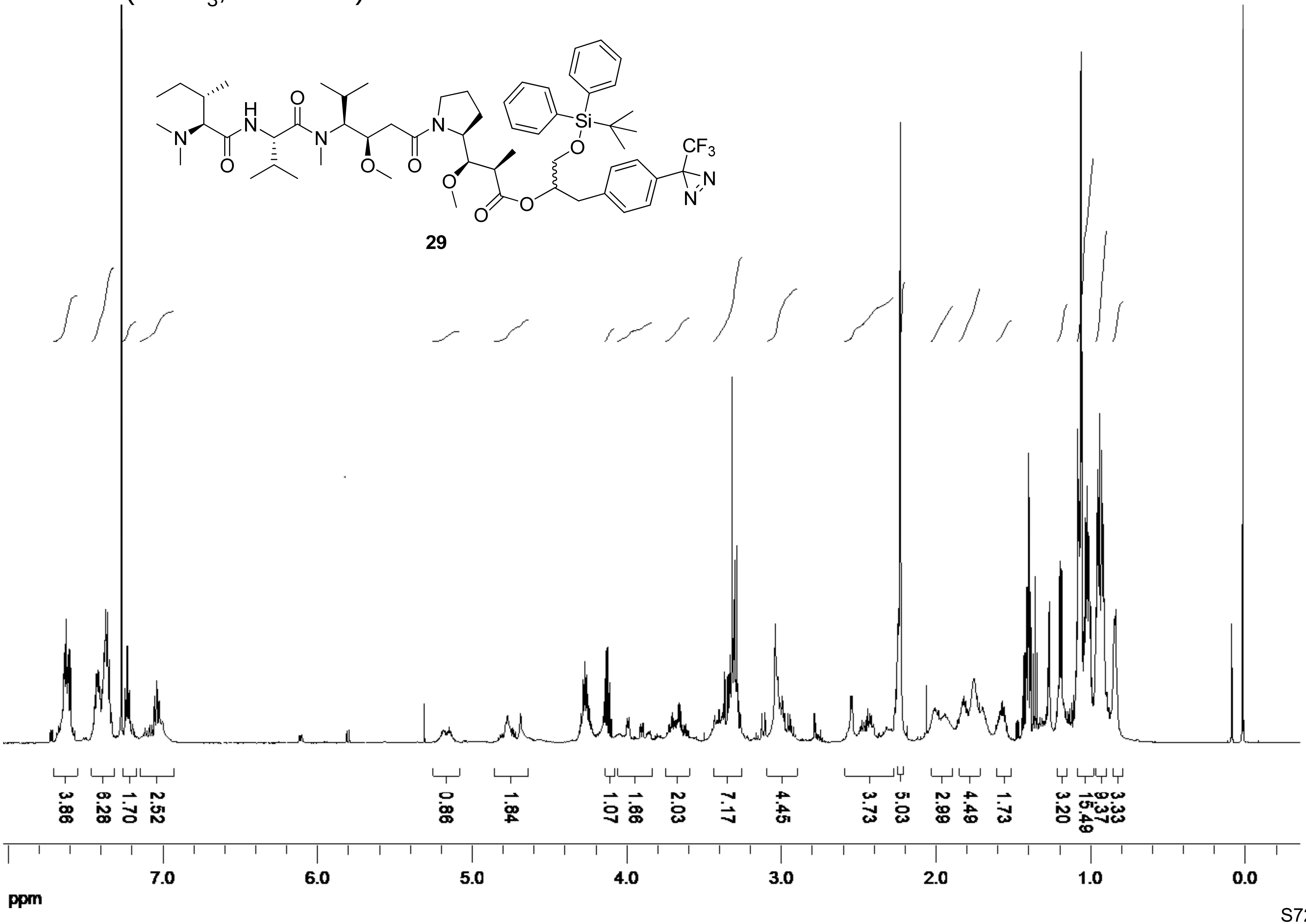

TBDPS-Photo malevamide D (29)

^{13}C NMR ($CDCl_3$, 150 MHz)

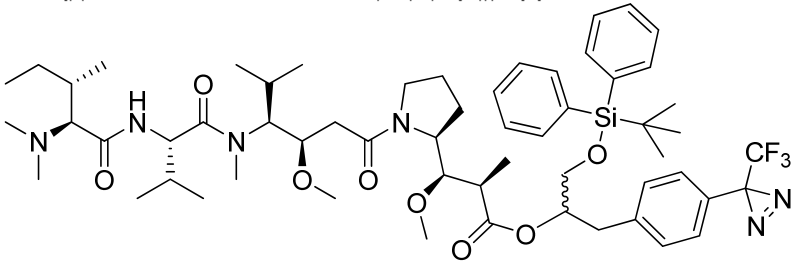

29

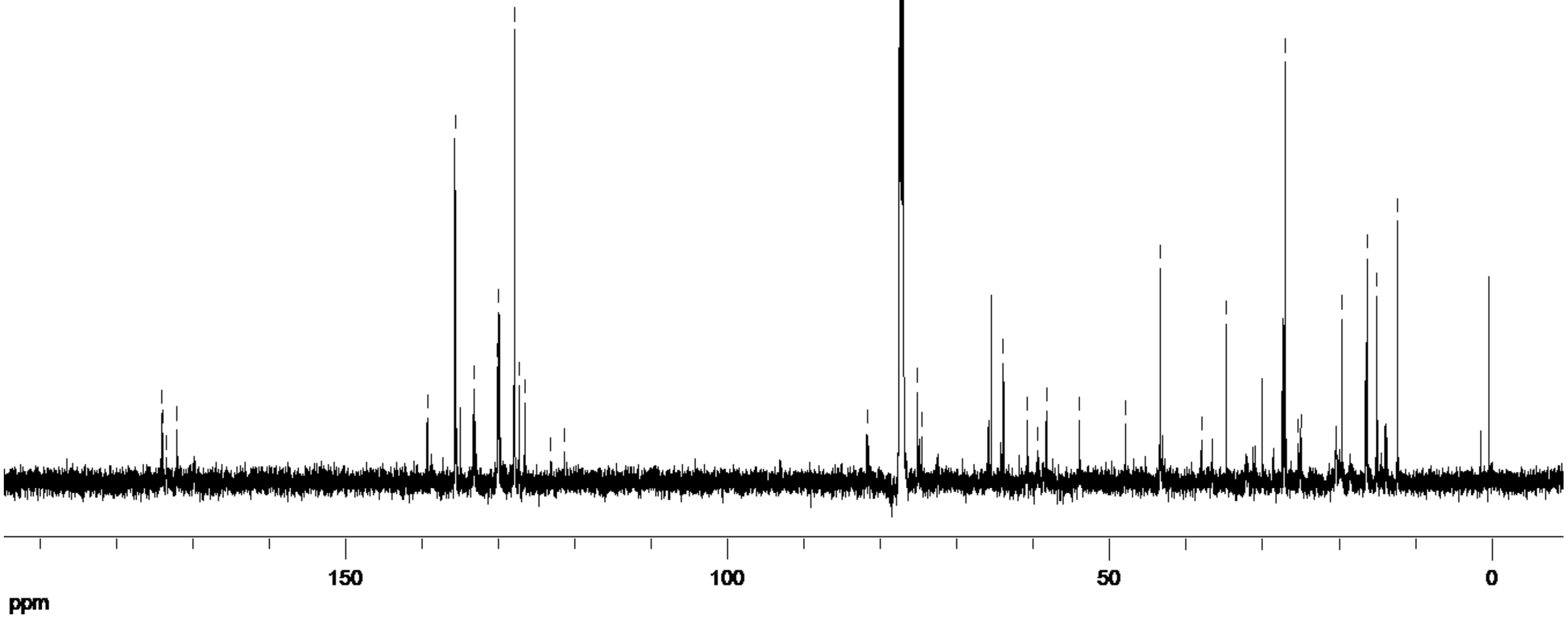

Photo malevamide D (30)

^{1}H NMR ($CDCl_3$, 600 MHz)

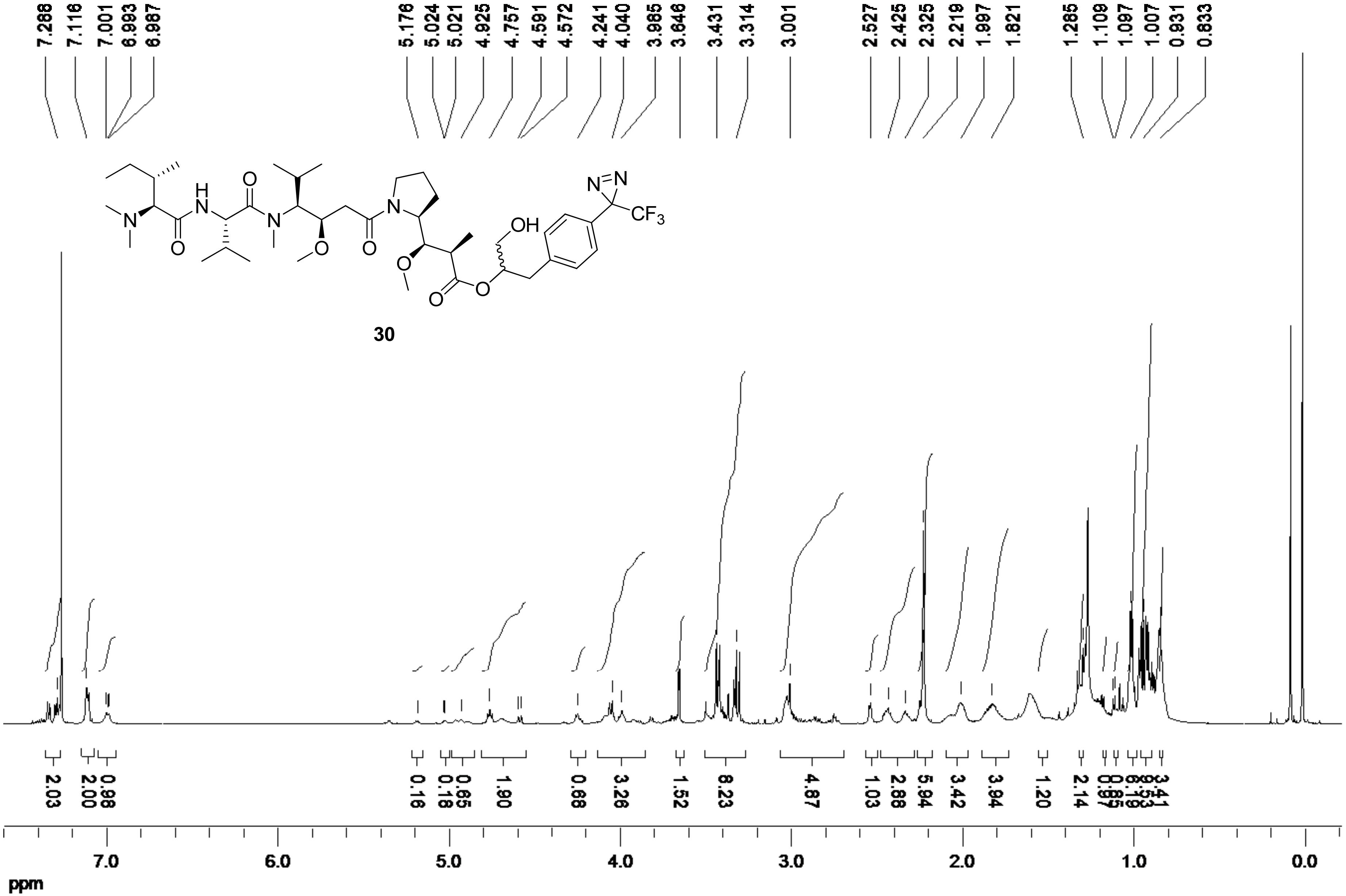

Photo malevamide D (30)

6

^{13}C NMR ($CDCl_3$, 150 MHz)

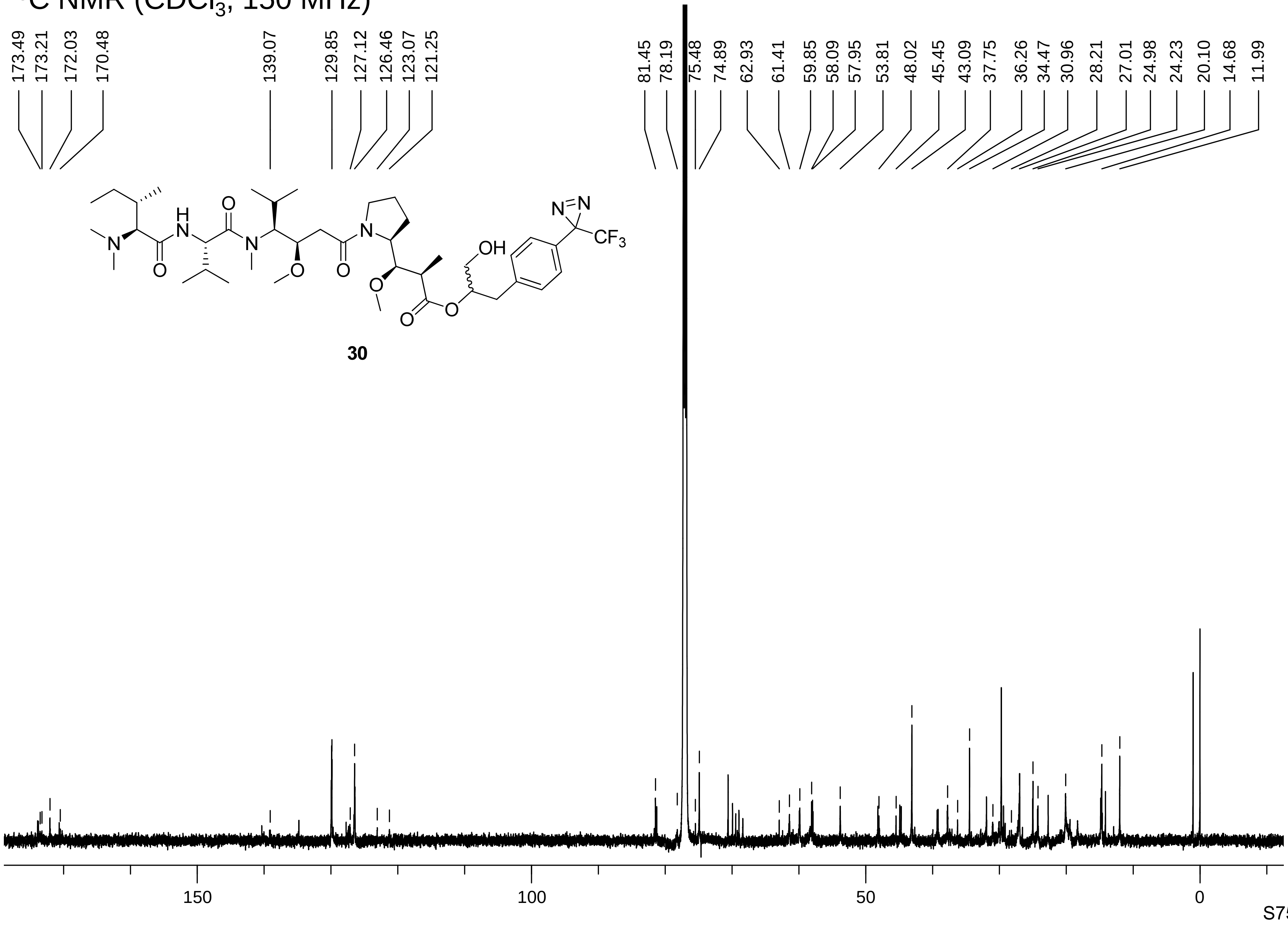

Photo PPD (25)

DSC diagram 5 degrees/min

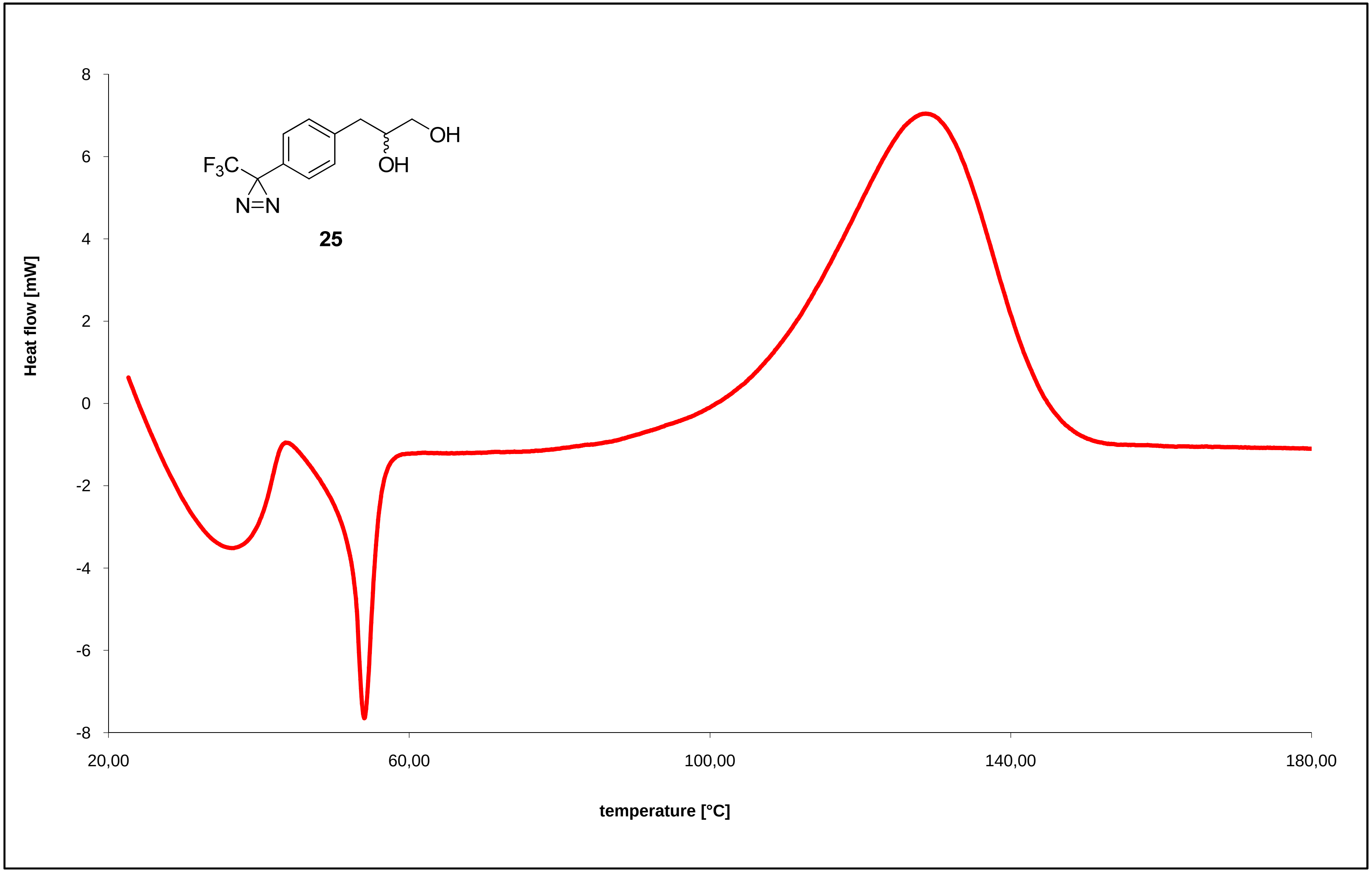

- **X-ray analysis (*E*)-tosyloxime 22**

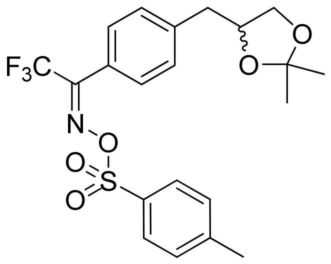

(*E*)-**22**

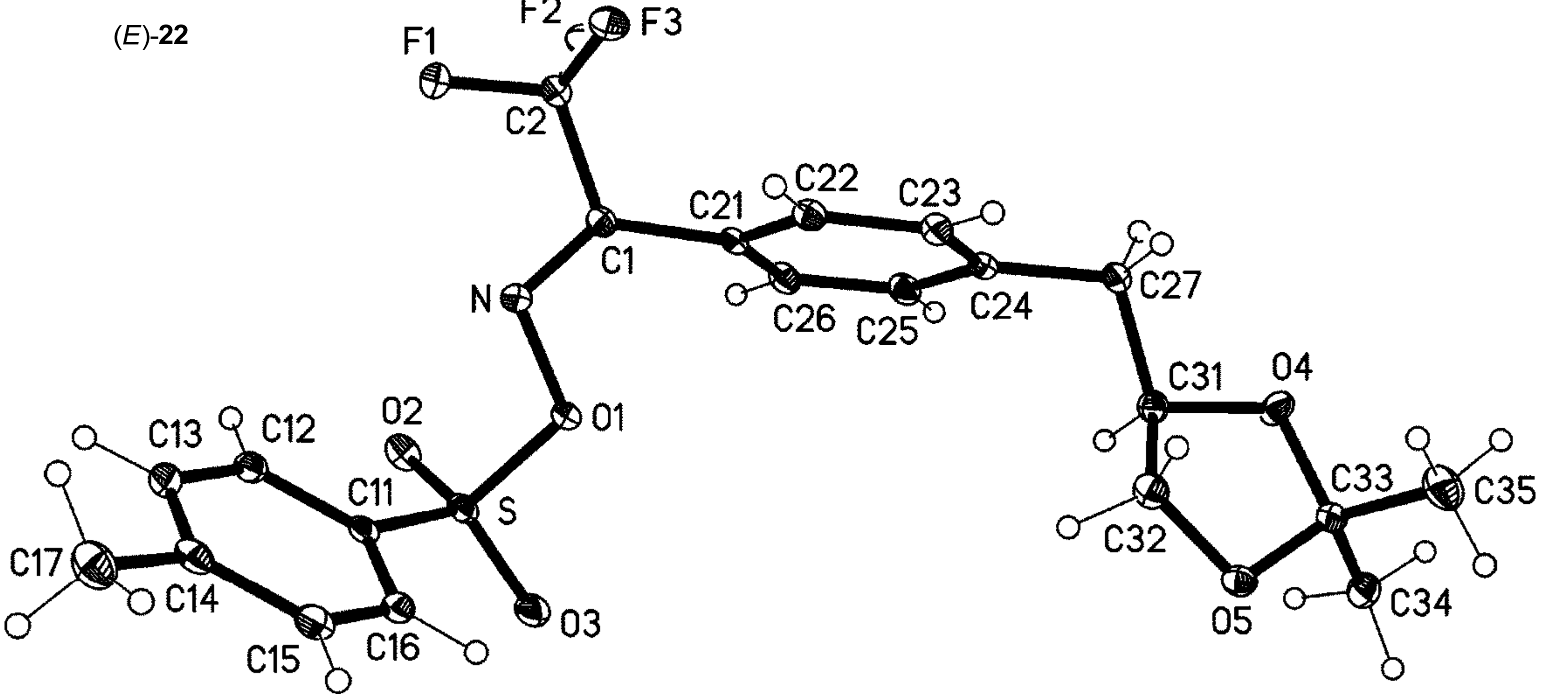

Supplement: File 1 — Procedures of synthesis and biotest, X-ray data and 1H, 13C NMR spectra of selected compounds. [file Beilstein_J_Org_Chem-10-316-s001.pdf]
